# Cationic Heterobimetallic Mg(Zn)/Al(Ga) Combinations for Cooperative C–F Bond Cleavage

**DOI:** 10.1002/anie.202103250

**Published:** 2021-06-19

**Authors:** Alexander Friedrich, Jonathan Eyselein, Jens Langer, Christian Färber, Sjoerd Harder

**Affiliations:** ^1^ Inorganic and Organometallic Chemistry Universität Erlangen-Nürnberg Egerlandstrasse 1 91058 Erlangen Germany

**Keywords:** aluminum, C–F bond activation, gallium, magnesium, metal–metal bonding, zinc

## Abstract

Low‐valent (^Me^BDI)Al and (^Me^BDI)Ga and highly Lewis acidic cations in [(^tBu^BDI)M^+^⋅C_6_H_6_][(B(C_6_F_5_)_4_^−^] (M=Mg or Zn, ^Me^BDI=HC[C(Me)N‐DIPP]_2_, ^tBu^BDI=HC[C(*t*Bu)N‐DIPP]_2_, DIPP=2,6‐diisopropylphenyl) react to heterobimetallic cations [(^tBu^BDI)Mg–Al(^Me^BDI)^+^], [(^tBu^BDI)Mg–Ga(^Me^BDI)^+^] and [(^tBu^BDI)Zn–Ga(^Me^BDI)^+^]. These cations feature long Mg–Al (or Ga) bonds while the Zn–Ga bond is short. The [(^tBu^BDI)Zn–Al(^Me^BDI)^+^] cation was not formed. Combined AIM and charge calculations suggest that the metal–metal bonds to Zn are considerably more covalent, whereas those to Mg should be described as weak Al^I^(or Ga^I^)→Mg^2+^ donor bonds. Failure to isolate the Zn–Al combination originates from cleavage of the C−F bond in the solvent fluorobenzene to give (^tBu^BDI)ZnPh and (^Me^BDI)AlF^+^ which is extremely Lewis acidic and was not observed, but (^Me^BDI)Al(F)‐(μ‐F)‐(F)Al(^Me^BDI)^+^ was verified by X‐ray diffraction. DFT calculations show that the remarkably facile C–F bond cleavage follows a dearomatization/rearomatization route.

## Graphical abstract

Together strong: Electron‐rich low‐valent Al^I^ and electron‐poor Zn^2+^ centers cooperatively cleave the unactivated, strong C−F bond in fluorobenzene via a dearomatization–rearomatization mechanism.

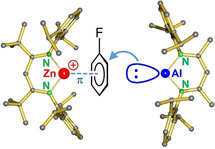

## Introduction

Mixing complexes of different metals creates reactivities that are greater than the sum of its parts. A prime example is the synthesis of powerful Lochmann‐Schlosser *superbases* by addition of MO*t*Bu (M=Na, K) to *n*BuLi, a method based on earlier observations by Morton and Claff.[^1^, ^2^, ^3^] Although identification of the structures of alkali metal cocktails has been challenging,[^4^, ^5^] continuous improvements of analytical equipment enabled comprehensive structural insights.[^6^, ^7^, ^8^] Apart from cooperating with each other, synergistic effects between the alkali metals and nearly all metals in the periodic table have now been established.^[9]^ In particular, mixtures of alkali metals with Mg, Zn, Al or Ga have shown unique reactivities and selectivities^[10]^ that are currently increasingly exploited in heterobimetallic catalysis.[^11^, ^12^, ^13^, ^14^, ^15^, ^16^, ^17^, ^18^]

The creation of new reactivities by metal mixing also entered the field of metal–metal bonding.[^19^, ^20^, ^21^, ^22^] The unique reactivity of homometallic, low‐valent main group metal complexes can be further enriched by heterobimetallic metal–metal bound complexes. A most recent example represents the introduction of R_2_Al‐K reagents[^23^, ^24^, ^25^, ^26^, ^27^, ^28^, ^29^] which based on the metal's electronegativity differences should be classified as R_2_Al^−^K^+^ reagents with nucleophilic aluminyl units (e.g. **I**). The K^+^ cation in these and related systems has been shown to play an eminent role in their reactivity and selectivity,^[30]^ for example, in the *para*‐selective dialumination of benzene via transition state **II**.^[29]^ Most recently, we reported similar RMgNa complexes (**III**) which based on metal electronegativities and reactivity should be seen as RMg^−^Na^+^ reagents with nucleophilic magnesyl units.^[31]^ These electron‐rich reagents have considerable potential in bond activation by oxidative addition or in creation of Mg–metal bonds. The advantages of mixing metals is not restricted to main group metals but is also actively pursued in transition metal catalysis.[^32^, ^33^, ^34^, ^35^]


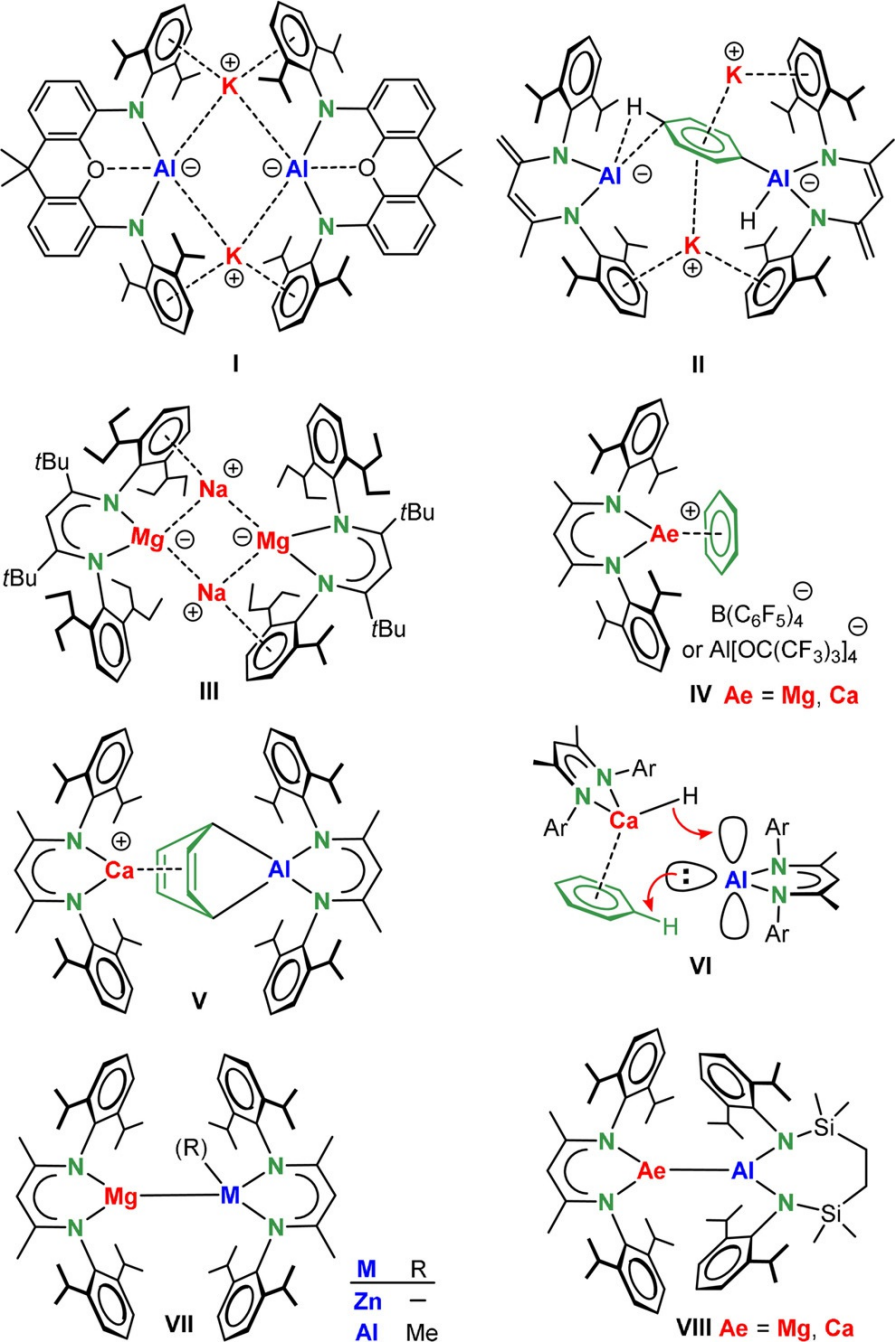


We and others recently reported a series of cationic β‐diketiminate alkaline earth (Ae) metal complexes (**IV**)[^36^, ^37^] which, being free of additional Lewis bases, display high Lewis acidity.[^38^, ^39^, ^40^] It was shown that a combination of the Lewis acidic cation (^Me^BDI)Ca^+^ and Roesky's low‐valent β‐diketiminate Al^I^ complex (^Me^BDI)Al^[41]^ is able to reduce benzene (**V**).^[42]^ Mixing (^Me^BDI)Al with [(^Me^BDI)Ca(μ‐H)]_2_ led to C−H activation of benzene (**VI**) to give (^Me^BDI)Al(H)Ph.^[43]^ The latter oxidative addition of (^Me^BDI)Al at a benzene C−H bond is catalytic in the calcium hydride reagent. Crimmin and co‐workers showed that low‐valent (^Me^BDI)Mg–Mg(^Me^BDI) complexes are able to cleave Ar‐F bonds by oxidative addition, provided the Ar rest is electron‐poor and carries at least four F‐substituents.^[44]^ It was attempted to raise the reactivity of the low‐valent complex by using mixed‐metal complexes with polarized metal–metal bonds (e.g. **VII**), however, these mixed‐metal complexes were found to be less reactive than homometallic (^Me^BDI)Mg‐Mg(^Me^BDI).^[45]^ Most recently, the Hill group introduced low‐valent mixed‐metal Ae‐Al complexes **VIII** (Ae=Mg, Ca) with polarized Ae^δ+^–Al^δ−^ bonds.^[26]^ Although the Ca–Al complex showed a higher reactivity than the Mg–Al complex, both are stable in benzene. This contrasts strongly with the high reactivity of the (^Me^BDI)Ca^+^/(^Me^BDI)Al^I^ combination which instantaneously reduces benzene at room temperature (**V**).^[42]^ The cationic nature of this mixed‐metal reagent could be the key to its high reactivity. Increased reactivity of cationic vs. neutral complexes was also observed by Okuda and has been attributed to higher metal Lewis acidity imparted by the positive charge on the complex. Thus, cationic Ca hydride complexes showed increased reactivity^[46]^ and activity in alkene hydrogenation catalysis.^[47]^ This motivated our exploration of cationic heterobimetallic complexes with reactive metal–metal bonds. Here we report a series of cationic heterobimetallic complexes that have been obtained by combining (BDI)Mg^+^ or (BDI)Zn^+^ cations with low‐valent (BDI)Al^I^ or (BDI)Ga^I^ complexes. We demonstrate that metal choice strongly influences structure, bonding and reactivity and report facile cleavage of the non‐activated C−F bond in fluorobenzene by the Zn‐Al combination. DFT calculations suggest a novel dearomatization–rearomatization mechanism for C–F bond cleavage.

## Results and Discussion

**Scheme 1:**
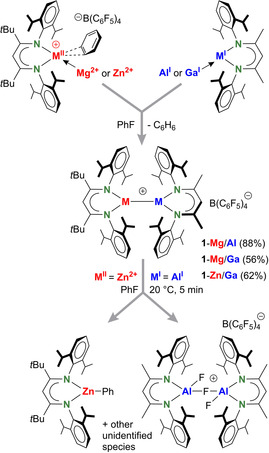
Synthesis of cationic Mg(or Zn)/Al(or Ga) complexes and decomposition of **1**‐Zn/Al by C–F bond activation in fluorobenzene.

For the cationic fragment we chose the recently reported (^tBu^BDI)Mg^+^ or (^tBu^BDI)Zn^+^ entities stabilized by a large ^tBu^BDI ligand with *t*Bu groups in the backbone.[^48^, ^49^] This bulky ligand prevents contact between the cation and the B(C_6_F_5_)_4_^−^ anion, leaving the metal free for solvent interaction^[50]^ or molecule activation (Scheme 1). Reaction of their benzene adducts with the low‐valent species (^Me^BDI)Al or (^Me^BDI)Ga^[51]^ in fluorobenzene gave crystals of the heterobimetallic complexes **1**‐Mg/Al, **1**‐Mg/Ga and **1**‐Zn/Ga in good to excellent yields (56–88 %). These complexes can also be obtained by in situ generation of the cationic fragments followed by introduction of the low‐valent Al^I^ or Ga^I^ reagent (see ESI).

Attempts to obtain the heterobimetallic complex **1**‐Zn/Al failed. A solution of [(^tBu^BDI)Zn^+^⋅C_6_H_6_][B(C_6_F_5_)_4_^−^] and (^Me^BDI)Al in fluorobenzene led to rapid solvent decomposition (Scheme 1). The Zn/Al pair cleaved the C−F bond in fluorobenzene instantaneously at room temperature and (^tBu^BDI)ZnPh was isolated in 75 % yield (crystal structure: Figure S56). The recently reported cation (^tBu^BDI)Zn^+^⋅(π‐C_6_H_5_F), in which fluorobenzene binds Zn via its π‐system,^[49]^ showed identical reactivity with (^Me^BDI)Al, indicating that the Ph ring in (^tBu^BDI)ZnPh originates from fluorobenzene and not from benzene. The onward reaction of (^tBu^BDI)ZnPh with I_2_ is clean and gave (^tBu^BDI)ZnI and PhI (Figure S33–S38). The other product, (^Me^BDI)AlF^+^, could not be isolated nor observed which is likely due to its very high reactivity. The Al center in the cation (^Me^BDI)AlF^+^ would be even more Lewis acidic and reactive than that in hypothetical alumoxane (^Me^BDI)AlO^[52]^ and further fluoride abstraction or bond activation reactivity could be anticipated. Indeed, in one case crystals containing the cation (^Me^BDI)Al(F)‐(μ‐F)‐(F)Al(^Me^BDI)^+^ were isolated (Figure S52). Attempts to prevent decomposition of the Zn/Al complex by maintaining the fluorobenzene solution at −30 °C led to crystallization of educt [(^tBu^BDI)Zn^+^⋅(π‐C_6_H_5_F)][B(C_6_F_5_)_4_^−^].

**Figure 1:**
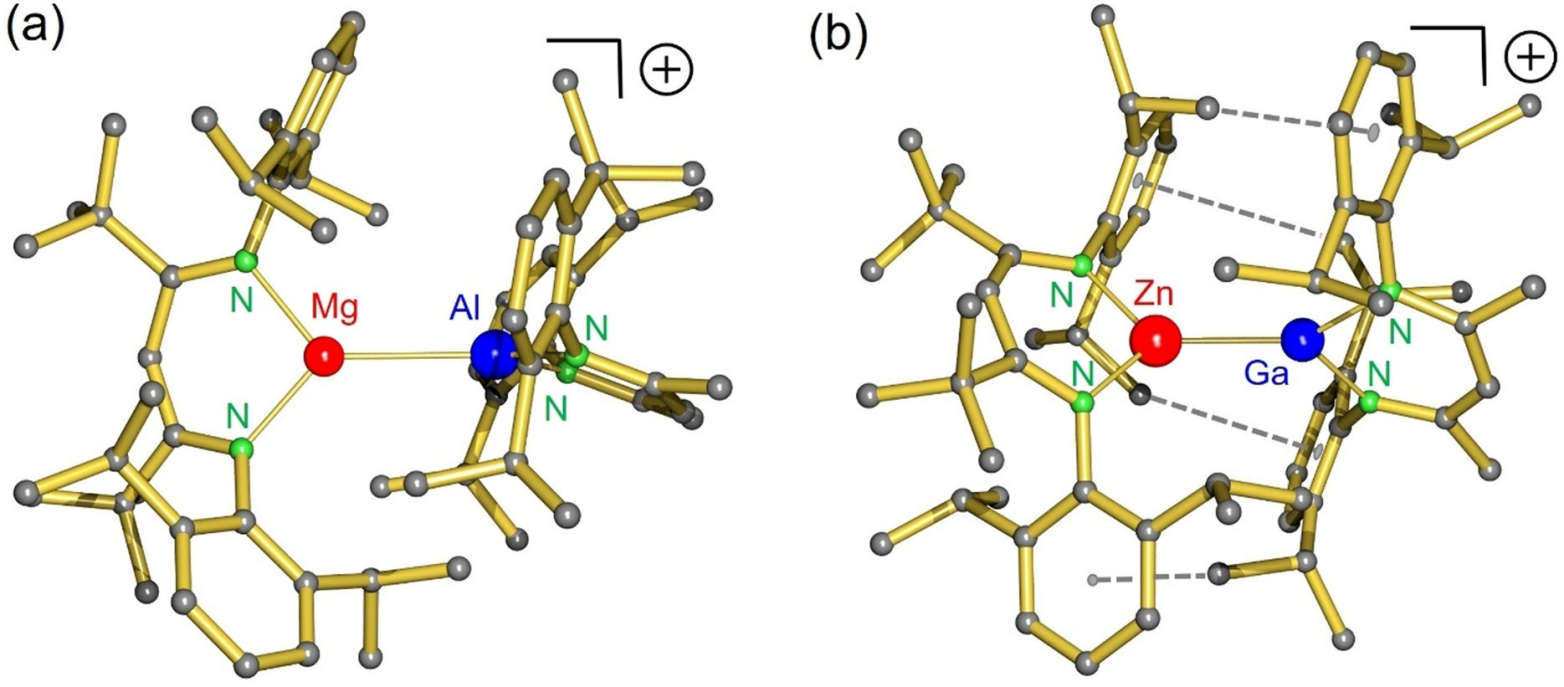
a) Cation in the crystal structure of **1**‐Mg/Al; the similar structure of **1**‐Mg/Ga is shown in Figure S54. b) Cation in the crystal structure of **1**‐Zn/Ga. Striped lines indicate short C−H⋅⋅⋅π interactions between *i*Pr Me‐substituents and aromatic rings.

**Scheme 2:**
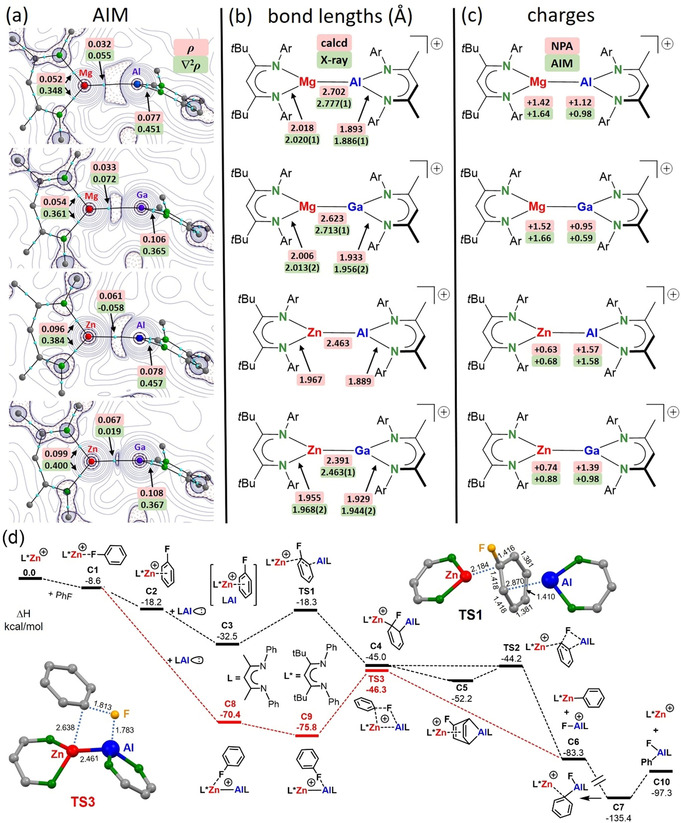
a) AIM contour plots showing the Laplacian distribution of the electron density in the calculated cations (^tBu^BDI)M–M′(^Me^BDI)^+^. Blue dots are bond‐critical‐points. b) Comparison of calculated and experimental bond lengths. c) Calculated NPA and AIM charges. d) Energy profile for C–F bond activation in fluorobenzene by a combination of L*Zn^+^ and LAl^I^; ωB97XD/6–311+G**//ωB97XD/6–31+G** with solvent correction (PCM=fluorobenzene); Δ*H* in kcal mol^−1^.

The three complexes **1**‐Mg/Al, **1**‐Mg/Ga and **1**‐Zn/Ga crystallize isomorphous showing structures in which the heterobimetallic cation is fully separated from the B(C_6_F_5_)_4_^−^ anion (Figure 1). The metals are not disordered, implying there is no metal–metal exchange between ^Me^BDI and ^tBu^BDI ligands. Metal–metal bond lengths (Scheme 2 b) are close to the sum of their covalent radii (Table 1). The Mg–Al bond of 2.7767(6) Å in **1**‐Mg/Al is significantly longer than the Mg–Ga or Zn–Ga bonds but falls in the range of reported Mg–Al bonds (2.696(1)–2.7980(6) Å, average: 2.743 Å).[^23^, ^26^, ^43^, ^45^, ^53^] Also the Mg–Ga bond of 2.7125(9) Å compares well to previously reported values (2.717(2)–2.7470(7) Å, average: 2.730 Å).^[54]^ The shortest metal–metal bond in the series, Zn–Ga (2.4634(6) Å), is in the range of other Zn‐Ga bonds (2.3230(7)–2.585(1) Å, average: 2.412 Å).[^55^, ^56^, ^57^, ^58^, ^59^, ^60^] This close Zn–Ga contact provokes various short C−H⋅⋅⋅π interactions between *i*Pr groups of one ligand with aryl rings of the other ligand (Figure 1 b). A similar embrace of BDI ligands was found in crystal structures of the homoleptic complexes (^Me^BDI)_2_Ae (Ae=Mg, Ca, Sr, Ba).^[61]^ Other distinct features are the rather small Al–N (1.883(1)–1.889(1) Å) and Ga–N (1.944(2)–1.956(2) Å) bonds which are considerably contracted compared to metal−N bonds in (^Me^BDI)Al (1.957(2) Å)^[41]^ or (^Me^BDI)Ga (2.055(1) Å).^[51]^ In contrast, Mg–N and Zn–N bonds are elongated by circa 0.02–0.05 Å in comparison to those in the cationic precursors. This points to significant polarization of the electron pair on Al and Ga to Mg and Zn. The C−C and C−N bond distances in the BDI ligands are not notably different from those in the precursors.

**Table 1:** Electronegativities (*χ*) and radii for the metals Mg, Zn, Al, and Ga.

	Mg	Zn	Al	Ga
*χ*(Pauling)^[66]^	1.31	1.66	1.61	1.81
*χ*(Allred‐Rochow)^[67]^	1.23	1.66	1.47	1.82
*χ*(configuration energy)^[68]^	1.29	1.66	1.61	1.76
Ionic radius [Å]^[69]^	0.72	0.74	0.54	0.62
Covalent radius [Å]^[70]^	1.39	1.18	1.26	1.24

While **1**‐Zn/Ga is reasonably soluble in C_6_D_5_Br, complexes **1**‐Mg/Al and **1**‐Mg/Ga could only be analyzed by NMR spectroscopy in the more polar solvent C_6_D_5_F. **1**‐Mg/Ga shows two sets of broadened ^1^H NMR signals which are shifted in respect to signals for the reactants. The chemical shifts are temperature sensitive and at +80 °C only sharp signals for the educts are observed (Figure S16), indicating an asssociation‐dissociation equilibrium. Complex **1**‐Mg/Al also shows broad ^1^H NMR signals with a strong temperature dependency but even at +80 °C full dissociation cannot be observed (Figure S8), indicating that the Mg–Al bond is stronger than the Mg–Ga bond. Complex **1**‐Zn/Ga in C_6_D_5_Br (or C_6_D_5_F) gave for each *i*Pr group unique ^1^H NMR signals, that is, 8 methine resonances and 16 methyl resonances (Figure S17), which is in agreement with the tight embrace of BDI ligands as observed in the crystal structure (Figure 1 b). In contrast to similar BDI⋅⋅⋅BDI interactions in (^Me^BDI)_2_Ae complexes,^[61]^ heating a solution of **1**‐Zn/Ga in C_6_D_5_Br to +80 °C did not result in coalescence of ^1^H NMR signals (Figure S25), supporting a tightly bound complex. From these dynamic NMR studies in fluorobenzene it can be deduced that the metal–metal bond strength increases along the series: Mg–Ga < Mg–Al < Zn–Ga.

All four heterobimetallic cations, including the one in hypothetical **1**‐Zn/Al, have been analyzed by DFT methods (ωB97XD/6–311+G**//ωB97XD/6–31+G**), Atoms‐In‐Molecules (AIM) and Natural‐Bond‐Orbital (NBO) analysis (Scheme 2). The calculated geometries fit quite well with the crystal structures (Scheme 2 b) apart for the metal–metal bonds which are calculated systematically 0.07–0.08 Å too short. This discrepancy was previously also noted for Mg–Mg complexes.[^67^, ^68^] Although Zn^2+^ and Mg^2+^ have similar ionic radii (Table 1), metal–metal bonds to Mg are systematically 0.23–0.24 Å longer than those to Zn. This is a first indication that metal bonds to Mg and Zn differ in nature. The shortest metal–metal bond of 2.391 Å is calculated for the cation in **1**‐Zn/Ga. Since the calculated Zn‐Al bond of 2.463 Å for the cation in **1**‐Zn/Al is slightly longer, failure to isolate **1**‐Zn/Al is not related to steric problems. The considerably shorter bonds to Zn are likely related to a higher degree of covalency:^[49]^ Zn, Al and Ga have similar electronegativities whereas Mg is significantly less electronegative (Table 1).

Bonding metal–metal interactions in the four heterobimetallic cations are indicated by bond‐critical‐points (*bcp*’s) in the AIM analysis (Scheme 2 a). Contour plots of the negative Laplacian, −∇^2^ *ρ*(*r*), show the differences in electron distribution along the metal–metal axes. The lone‐pair of electrons on Al^I^ is much more pronounced than that on Ga^I^. It is strongly polarized towards Mg in **1**‐Mg/Al but, unlike the more symmetrically arranged electron distribution in a Mg–Mg bond,[^69^, ^70^, ^71^] it should not be described as a Non‐Nuclear‐Attractor (NNA). The low electron density and small positive value for ∇^2^ *ρ* in the bond‐critical‐point (*bcp*) indicate a weak electrostatic closed‐shell interaction best described as (^Me^BDI)Al coordination to the (^tBu^BDI)Mg^+^ cation. The considerably higher degree of covalency in **1**‐Zn/Al is demonstrated by a higher electron density *ρ*(*r*) in the *bcp* and a small negative value for ∇^2^ *ρ*(*r*), typically found for covalent bonds. Also the Zn–Ga bond is characterized by higher electron density in the *bcp* and although ∇^2^ *ρ*(*r*) is not negative, it is close to zero. This is corroborated by the total energy density ratio in the *bcp* which is defined as H(*r*)/*ρ*(*r*) and is close to zero for ionic bonds but becomes more negative for covalent bonds. The calculated values (in a.u.) clearly demonstrate that bonds to Zn are more covalent: Mg–Al −0.170, Mg–Ga −0.136, Zn–Al −0.423 and Zn–Ga −0.402.

The NPA charges on Mg in the Mg–Al (+1.42) and Mg–Ga (+1.52) complexes are only slightly lower than that in the free cation (^tBu^BDI)Mg^+^ (+1.82); Scheme 2 c. In contrast, the NPA charges on Zn in Zn‐Al (+0.63) and Zn‐Ga (+0.74) complexes are considerably lower than that in the free cation (^tBu^BDI)Zn^+^ (+1.43). Consistently, the Mg–Al and Mg–Ga complexes show relatively low positive charges on Al (+1.12) and Ga (+0.95). These charges are close to those in (^Me^BDI)Al (+0.82) and (^Me^BDI)Ga (+0.79). On the other hand, the Zn–Al and Zn–Ga complexes show much higher charges on Al (+1.57) and Ga (+1.39). These charge distributions are consistent with the view that the most electropositive metal Mg forms electrostatic bonds with electron‐rich Al^I^ and Ga^I^ “ligands” in which the electron pair is mainly located on the *p*‐block metal. Bonds between the more electronegative Zn and Al or Ga are more covalent in character and there is considerable charge transfer from the *p*‐block metal to Zn. AIM analyses (vide supra) support this view. The HOMO–LUMO presentations of all cationic heterobimetallic complexes (Figures S61–64) consistently show HOMO's mainly located on the BDI ligand at Mg or Zn while the HOMO−1 either has the character of a lone‐pair at Al or Ga (for Mg–Al and Mg–Ga) or indicates more covalent metal–metal bonding (for Zn–Al and Zn–Ga). The LUMO's are in all cases mainly concentrated on the BDI ligand at Al or Ga while the LUMO+1 has metal–metal π‐bond character.

The instantaneous cleavage of the C–F bond in an unactivated substrate like C_6_H_5_F by a mixture of [(^tBu^BDI)Zn^+^⋅C_6_H_6_][B(C_6_F_5_)_4_^−^] and (^Me^BDI)Al at room temperature is remarkable. Dinuclear Mg^I^ complexes of type (BDI)MgMg(BDI) only cleave activated C–F bonds in polyfluorinated aromatics. For thermodynamic as well as kinetic reasons, at least four F‐substituents are needed and the presence of an *ortho*‐F atom has been found highly beneficial.^[45]^ Also (^Me^BDI)Al only reacts with activated polyfluorated aromatics (at least three F‐substituents are needed).^[72]^ There are very few main group metal systems that are able to cleave the C−F bond in C_6_H_5_F. We recently reported C−F bond cleavage in C_6_H_5_F with a highly reactive dinuclear Mg complex with a bridging C_6_H_6_^2−^ anion but conditions were harsh (5 days, 100 °C).^[67]^ More recently it was shown that C_6_H_5_F reacts at room temperature with photoactivated (^Me^BDI)MgMg(^Me^BDI) in a radical process resulting in [(^Me^BDI)MgF]_2_ and biphenyl.^[73]^ Hill reported that the highly reactive Ca hydride complex [(^Me^BDI)Ca(μ‐H]_2_ slowly reacts with C_6_H_5_F to give an intractable mixture of products,^[74]^ while a cationic Ca hydride complex from the Okuda group reacted with C_6_H_5_F to a Ca fluoride complex (60 °C, 24 h), however, the latter is likely formed by direct nucleophilic substitution and not by oxidative addition. Crimmin and co‐workers reported a Pd^0^ catalyzed oxidative addition of (^Me^BDI)Al to Ph‐F which is instanteneous at room temperature.^[75]^ The mildest conditions (−30 °C) reported for oxidative addition of Al^I^ to Ph‐F need support from Rh^I^.^[76]^

Inspired by the facile C−F bond cleavage in C_6_H_5_F, we probed whether even more electron‐rich fluoroarenes could be converted. The combination of [(^tBu^BDI)Zn^+^⋅(C_6_H_6_)][B(C_6_F_5_)_4_^−^] and (^Me^BDI)Al reacted instantaneously when dissolved in *p*‐fluorotoluene, indicated by a rapid color change from light‐yellow to orange. Extensive NMR investigation of the hexane‐soluble fraction, using two‐dimensional methods and DOSY, show formation of (^tBu^BDI)Zn(*p*‐tolyl), a product which was also confirmed by X‐ray diffraction (Figure S57). NMR data for the second product (Figures S40–51) have similarities with NMR data earlier reported for (^Me^BDI)Al(Ph)F which was formed by Pd‐catalyzed oxidative addition of (^Me^BDI)Al to Ph‐F.^[75]^ Our comprehensive NMR study indicates formation of (^Me^BDI)Al(*p*‐tolyl)F but poor crystallization did not allow for confirmation by X‐ray diffraction. The simultaneous formation of Zn and Al *p*‐tolyl species is likely due to ligand scrambling that is controlled by thermodynamics and product solubilities (vide infra).

The herein observed facile reductive C–F bond cleavage by oxidative addition to a Zn/Al combination is due to a synergistic effect: the cationic Zn fragment (^tBu^BDI)Zn^+^ and (^Me^BDI)Al alone do not react with C_6_H_5_F, but Zn‐Al cooperation cleaves the C−F bond readily. This high reactivity is likely due to its cationic nature but also to the choice of metals. Other cationic Mg/Al, Mg/Ga or Zn/Ga complexes, but also the Ca/Al pair (**V**),^[42]^ do not react with fluorobenzene. It is noteworthy that while the C–F bond in the fluorobenzene solvent is rapidly cleaved by the cationic Zn/Al combination, there is no indication for C–F bond activation in the anion B(C_6_F_5_)_4_^−^. This may be related to the bulky ^tBu^BDI ligand which prevents formation of a (^tBu^BDI)Zn^+^⋅⋅⋅B(C_6_F_5_)_4_^−^ contact ion‐pair but allows for (^tBu^BDI)Zn^+^⋅(π‐C_6_H_5_F) formation.^[49]^ In contrast to (^tBu^BDI)Mg^+^, which binds C_6_H_5_F by Mg⋅⋅⋅F interaction,^[50]^ the (^tBu^BDI)Zn^+^ binds C_6_H_5_F as a π‐complex that is accessible for nucleophilic attack at the Ph ring.

DFT calculations on a model system in which DIPP‐substituents were replaced for Ph groups and B(C_6_F_5_)_4_^−^ was neglected give insights in the possible mechanism for synergistic C–F bond activation (Scheme 2 d). Starting with the “naked” L*Zn^+^ cation (L*=HC[C(*t*Bu)NPh]_2_), PhF has strong preference for π‐bonding (**C1** vs. **C2**). This strongly contrasts with (^tBu^BDI)Mg^+^⋅⋅⋅F‐Ph bonding, with a clear preference for Mg⋅⋅⋅F interaction,^[50]^ but is in agreement with isolation of a π‐complex similar to **C2** which was structurally characterized by X‐ray diffraction.^[49]^ Previously reported DFT calculations show a total charge of +0.15 on the PhF ligand which indicates some extent of charge transfer and partially covalent Zn⋅⋅⋅Ph bonding (*cf*. for (^tBu^BDI)Mg^+^⋅⋅⋅F‐Ph a charge of +0.02 on PhF was calculated, indicating a merely electrostatic interaction). While the HOMO in L*Zn^+^⋅(π‐PhF) is located on the BDI ligand, the LUMO shows major coefficients on Zn and PhF (Figure S60). The π‐complex **C2** has only limited space for interaction with LAl^I^ (L=HC[C(Me)NPh]_2_) and forms a loosely bound complex (**C3**) with multiple C−H⋅⋅⋅π interactions among the BDI ligands (L and L*) and PhF. However, the free coordination site in **C1** allows for formation of a Zn‐Al bond (**C8**). Complex **C9**, with an Al⋅⋅⋅FPh interaction, is slightly more stable. The transition state for frontal attack is too high for a facile room temperature process (**C9**→**TS3**, +29.5 kcal mol^−1^). Although **TS3** is the typical σ‐bond metathesis transition state for (hetero)bimetallic C–F bond activation,^[45]^ we found an alternative mechanism. Starting from the π‐bound complex **C2**, in which PhF is activated for nucleophilic attack by complexation with L*Zn^+^, we searched the potential energy suface for a transition state with rear‐side attack by LAl^I^ according to a S_N_Ar mechanism. However, all efforts culminated in the identification of **TS1**, a transition state which is in line with nucleophilic 1,2‐addition to an aromatic C=C double bond. Despite dearomatization of the Ph ring, indicated by a non‐planar ring with localized C=C bonds, the barrier of +14.2 kcal mol^−1^ is in line with smooth C–F bond activation, as observed experimentally. An alternative minimum for 1,4‐addition was found (**C5**). The latter is reminiscent of complex **V**, which previously has been verified experimentally.^[42]^ Such cooperation of electron‐poor and electron‐rich metals could also be described as bond activation by a Frustrated‐Lewis‐Pair (FLP).^[77]^ The herein proposed cooperation between the Zn and Al metal centers has been verified by calculating the mechanism for the direct oxidative addition of LAl^I^ to the Ph‐F bond (Figure S59). In agreement with previous calculations,[^78^, ^79^] the high activation enthalpy of +27.8 kcal mol^−1^ for this reaction is nearly double that calculated for the conversion: **C3**→**TS1**→**C4**. Note that the **C3**→**C4** conversion is endothermic (due to loss of aromaticity) and that the C−F bond in **C4** (or **C5**) is still intact. Subsequent cleavage of the strong C–F bond by elimination of LAl‐F^+^ from **C4** (or **C5**) is essentially without barrier (**TS2**) and exothermic (**C4**→**C6**, −38.3 kcal mol^−1^). The latter step is strongly facilitated by rearomatization of the π‐system which supplies the energy needed for C–F bond cleavage. Nature uses similar dearomatization/rearomatization protocols for challenging transformations (e.g. NADH/NAD^+^). The same principle is also increasingly applied in the development of contemporary catalysts with ligand‐metal cooperation (e.g. of type Noyori, Shvo or Huang).^[80]^ The enormous Lewis acidity of LAl‐F^+^ is demonstrated by its ability to substract the Ph group from the product L*ZnPh, a process which in the absence of further F‐sources is highly exothermic (**C6**→**C7**, −52.1 kcal mol^−1^). Complex **C7** could further eliminate LAl(F)Ph to give L*Zn^+^ (**C10**), the starting point of the energy profile. This shows that the oxidative addition of LAl^I^ to the Ph‐F bond is overall an exothermic process and suggests that the reaction may be catalytic in L*Zn^+^. All attempts to run this C–F bond activation in a catalytic protocol failed. This is likely due to ligand scrambling equilibria that are controlled by thermodynamics and product solubilities. In the present case we observed L*ZnPh as the major product, explaining why the reaction is not catalytic in Zn.

As the herein presented energy profile does not include the influence of the weakly coordinating B(C_6_F_5_)_4_^−^ anion, it should be treated with care. According to our calculations, the complexes **C8** and **C9** should be quite stable and could be a thermodynamic sink, impeding the C–F bond activation. However, experimentally there is no indication for Zn‐Al bond formation: a solution of [(^tBu^BDI)Zn^+^][B(C_6_F_5_)_4_^−^] and (^Me^BDI)Al in PhF gave at low temperature crystallization of [(^tBu^BDI)Zn^+^⋅(π‐C_6_H_5_F)][B(C_6_F_5_)_4_^−^] which is the starting point for the low energy route **C3**→**TS1**→**C4**.

These data suggest that the facile C–F bond activation in fluorobenzene does not proceed through a previously formed cationic heterobimetallic complex but is the result of cooperating L*Zn^+^ and LAl^I^ species. Failure to locate a S_N_Ar pathway is likely due to unfavorable formation of L*Zn−F, a soft‐hard combination and the unusual stability of the π‐PhF complex **C2** vs. a L*Zn⋅⋅⋅FPh complex (**C1**) which would be the first step in a S_N_Ar mechanism. The here presented pathway results in LAl−F^+^, a hard‐hard combination, and L*ZnPh, which is in accordance with experimental observation.

## Conclusion

Combining the electron‐rich, low‐valent β‐diketiminate complexes (^Me^BDI)Al or (^Me^BDI)Ga with “naked” (Lewis base‐free) cations like (^tBu^BDI)Mg^+^ or (^tBu^BDI)Zn^+^ gave heterobimetallic cations with Mg−Al, Mg−Ga or Zn−Ga bonds. The Zn‐Al combination could not be obtained due to fast decomposition by reaction with fluorobenzene. Crystal structures of the borate salts of [(^tBu^BDI)Mg–Al(^Me^BDI)^+^], [(^tBu^BDI)Mg–Ga(^Me^BDI)^+^] and [(^tBu^BDI)Zn–Ga(^Me^BDI)^+^] reveal that B(C_6_F_5_)_4_^−^ is a truly non‐coordinating anion. Although Mg^2+^ and Zn^2+^ have comparable ionic radii, metal–metal bonds to Mg are considerably longer and weaker than those to Zn. This is supported by DFT calculations and bond analyses by AIM. Based on NPA charge calculations, the metal–metal bonds in the Mg complexes should be considered as Al^I^(or Ga^I^)→Mg^2+^ donor bonds while bonding in the Zn complexes is more covalent. Dynamic NMR studies in fluorobenzene indicate that the metal–metal bond strength increases along the series: Mg–Ga < Mg–Al < Zn–Ga. This is conform Hard‐Soft‐Acid‐Base (HSAB) theory according to which hard metal (Mg and Al) and soft metal (Zn and Ga) combinations form the strongest bonds.

The cationic Zn–Al complex, which according to HSAB is a soft‐hard mismatch, could not be obtained. While the metal–metal bound complex is not formed at lower temperatures, at room temperature fast cleavage of the C−F bond in the fluorobenzene solvent was observed. The products (^tBu^BDI)ZnPh and [(^Me^BDI)Al(F)‐(μ‐F)‐(F)Al(^Me^BDI)^+^][B(C_6_F_5_)_4_^−^] have been identified. This is a rare example of transition metal‐free cleavage of an unactivated C−F bond. Interestingly, the generally much more reactive polyfluorinated rings in the borate anion are left intact. Since the sterically congested (^tBu^BDI)Zn^+^ cation does not interact with B(C_6_F_5_)_4_^−^ but prefers formation of the solvent adduct (^tBu^BDI)Zn^+^⋅(π‐PhF), this complex with a π‐bound fluorobenzene ligand could be the key to C–F bond activation. The fluorobenzene ligand in the latter complex is bound to Zn with its π‐system and is activated for nucleophilic attack (calculated NPA charge on PhF: +0.15). Indeed, DFT calculations suggest a process in which (^Me^BDI)Al reacts with (^tBu^BDI)Zn^+^⋅(π‐PhF) by rear‐side 1,2‐addition to an aromatic C=C bond which is followed by (^Me^BDI)AlF^+^ elimination. The activation energy for this reaction is, despite loss of aromaticity, low: +14.2 kcal mol^−1^. The barriers for a direct, concerted C–F bond cleavage by either the (BDI)Al complex (+27.8 kcal mol^−1^) or by the heterobimetallic Al–Zn complex (+29.5 kcal mol^−1^) are both considerably higher.

The most important conclusion of this work is that the right combination of electron‐rich and electron‐poor metal centers can cooperate to rapidly cleave the strong, unactivated, C–F bonds in fluorobenzene and fluorotoluene at room temperature. The mechanism for this FLP‐type process is proposed to proceed through an unusual intermediate in which the phenyl ring is first dearomatized. Subsequent rearomatization delivers the energy needed for C–F bond cleavage.

## Conflict of interest

The authors declare no conflict of interest.

## Supporting information

As a service to our authors and readers, this journal provides supporting information supplied by the authors. Such materials are peer reviewed and may be re‐organized for online delivery, but are not copy‐edited or typeset. Technical support issues arising from supporting information (other than missing files) should be addressed to the authors.

Supplementary
