# Supplementary material for: Cationic Heterobimetallic Mg(Zn)/Al(Ga) Combinations for Cooperative C–F Bond Cleavage
Source: Angew Chem Int Ed Engl. 2021 Jun 19;60(30):16492–9. doi: 10.1002/anie.202103250 (PMC8361950; doi:10.1002/anie.202103250)
Supplement: Supplementary file 1 — Supplementary [file ANIE-60-16492-s001.pdf]

Angewandte Chemie
Eine Zeitschrift der Gesellschaft Deutscher Chemiker

Supporting Information

Cationic Heterobimetallic Mg(Zn)/Al(Ga) Combinations for Cooperative C–F Bond Cleavage

*Alexander Friedrich, Jonathan Eyselein, Jens Langer, Christian Färber, and Sjoerd Harder**

anie_202103250_sm_miscellaneous_information.pdf

SUPPORTING INFORMATION

Contents

1. Syntheses S2

2. Selected NMR spectra S5

3. Reactivity studies S19

4. Single crystal X-ray diffraction S35

5. Computational details S42

6. References S93

1. Syntheses

1.1 General procedures

All experiments were conducted under an inert nitrogen atmosphere using standard Schlenk and glovebox techniques (MBraun, Labmaster SP). All solvents were degassed with nitrogen, dried over activated aluminium oxide (Solvent Purification System: Pure Solv 400−4−MD, Innovative Technology) and stored over 3 Å molecular sieves unless noted otherwise. Fluorobenzene, chlorobenzene, bromobenzene, iodobenzene, *p*-fluorotoluene and *o*-fluoroanisole were dried over calcium hydride, distilled under N_2 atmosphere and stored over molecular sieves 3 Å. C_6D_6, C_6D_5Br (99.6% D, Sigma Aldrich) and C_6D_5F (99.6% D, Sigma Aldrich) were dried over 3 Å molecular sieves. $[(^{tBu}BDI)Mg{\cdot}benzene]^+[B(C_6F_5)_4]^-$,[S1] $(^{tBu}BDI)K$,[S2] $[(^{tBu}BDI)Zn{\cdot}benzene]^+[B(C_6F_5)_4]^-$,[S3] $(^{tBu}BDI)Mg$*n*Bu,[S4] $[CPh_3^+][B(C_6F_5)_4^-]$,[S5] $(^{Me}BDI)Al$[S6] and $(^{Me}BDI)Ga$[S7] were synthesized according to literature procedure. NMR spectra were recorded with a Bruker Avance III HD 400 MHz or a Bruker Avance III HD 600 MHz spectrometer. The spectra were referenced to the respective residual signals of the deuterated solvents. Elemental analysis was performed with an Euro EA 3000 (Euro Vector) analyzer. GC/MS measurements were performed on a Thermo Scientic™ Trace™ 1310 gas chromatography system (carrier gas Helium) with detection by a Thermo Scientic™ ISQ™ LT Single Quadrupole mass spectrometer. A Phenomenex® ZebronTM ZB-5 column of the dimensions 0.25 mm x 30 m with a film thickness of 0.25 µm was used. The samples (1 µl) were injected with an Instant Connect-SSL Module in the split mode (Injector Temperature: 280 °C). Temperature programs were started at 40 °C followed by heating ramps, optimized for the separation problem, until 280° C. Baseline separation of each analyte was achieved by choosing the different temperature programs. The molecular identity was confirmed by comparison with entries in the NIST/EPA/NIH mass spectral library (version 2.2, built June 10 2014). All crystal structures have been measured on a SuperNova (Agilent) diffractometer with dual Cu and Mo microfocus sources and an Atlas S2 detector. Crystallographic data have been deposited with the Cambridge Crystallographic Data Centre as supplementary publication no. 2067857-2067860 and no. 2081483 (see Table S1). Copies of the data can be obtained free of charge on application to CCDC, 12 Union Road, Cambridge CB21EZ, UK (fax: (+44)1223-336-033; E-mail: deposit@ccdc.cam.ac.uk). Diffusion measurements were conducted on a Bruker AVANCE NMR spectrometer operating at 600.13 MHz for proton resonance equipped with a 5 mm PABDO BB/19F-1H/D probe with Z-GRD and actively shielded gradient coil with a maximum gradient strength of 5.3500094 G/mm (at 10 A). Parameter optimization was carried out empirically employing the pulse programme ledbpgp2s1D using stimulated echo and LED (D21 = 5 ms, longitudinal eddy current delay as a Z-filter) with bipolar gradient pulses (P30) and two spoiling gradients (P19 = 600 µs) leading to values for gradient pulse length (P30 = adjusted [µs], in case of bipolar gradients *little DELTA*0.5*) and

diffusion time (D20 = 60 ms, *big DELTA*). Delay for gradient recovery was set to 200 µs. The diffusion experiment was executed with variable gradients from 2% to 98% gradient strength with 32 increment values (difframp calculated with the AU-program *DOSY*). In this case the pulse program ledbpgp2s was applied for data aquiring of this pseudo-2D Experiment. Data processing was performed with the T1/T2 software package (SimFit) of TopSpin (version 3.2, Bruker Biospin) by fitting area data (integration of all peaks of interest of the same molecule) of diffusion decays. From these Stejskal-Tanner fitting curves calculated diffusion constants were obtained and assimilated statistically.

1.2 Synthesis of complexes

[(^{tBu}BDI)Mg-Al(^{Me}BDI)$^+$][B(C_6F_5)$_4^-$]:

Method 1: [(^{tBu}BDI)Mg·benzene]$^+$[B(C_6F_5)$_4$]$^-$ (22.1 mg, 17.2 µmol, 1 eq.) and (^{Me}BDI)Al (7.7 mg, 17.2 µmol, 1 eq.) were dissolved in fluorobenzene (2 mL). The reaction mixture was stirred at room temperature for 6 h and subsequently layered with hexane (2 mL). A white precipitate formed which was isolated by removing the mother liquor. Washing of the solid with hexane (3 x 1 mL) gave the product [(^{tBu}BDI)Mg-Al(^{Me}BDI)$^+$][B(C_6F_5)$_4^-$] as a white solid in 75% yield (21.4 mg).

One-pot method 2: (^{tBu}BDI)Mg*n*Bu (15.0 mg, 25.7 µmol, 1.05 eq.) was dissolved in fluorobenzene (1 mL) and [CPh_3^+][B(C_6F_5)$_4^-$] (22.6 mg, 24.5 µmol, 1 eq.) was added. After shaking for 2 min the color change from orange to pale yellow indicated the generation of the cation. (^{Me}BDI)Al (10.9 mg, 24.5 µmol, 1 eq.) was added to the *in situ* generated magnesium cation and layering of the reaction mixture with hexane (0.5 mL) resulted in crystallization of the product. Isolation and washing with hexane (3 x 2 mL) gave [(^{tBu}BDI)Mg-Al(^{Me}BDI)$^+$][B(C_6F_5)$_4^-$] as microcrystalline solid in 88% yield (35.5 mg). Crystals suitable for X−ray diffraction were grown from the reaction mixture layered with hexane. **^{1}H NMR** (C_6D_5F, 400 MHz, 298 K): δ 7.82 (t, $^3J_{HH}$ = 7.7 Hz, 2H, aryl−*H*), 7.59 (m, 4H, aryl−*H*), other aryl−*H* obscured by C_6D_5F, 6.37 (s, 1H, CC*H*C), 6.02 (s, 1H, CC*H*C), 3.30 (very broad signals, 8H, C*H*(CH_3)$_2$), 2.10 (s, 6H, ^{Me}BDI), 1.76 (br. m, 12H, CH(C*H*$_3$)$_2$), 1.64 (br. m, 12H, CH(C*H*$_3$)$_2$), 1.59 (s, 18H, ^{tBu}BDI), 1.12 (br. m, 24H, CH(C*H*$_3$)$_2$) ppm. **^{11}B NMR** (C_6D_5F, 193 MHz, 298 K): δ −15.5 (s, 1B, *B*(C_6F_5)$_4^-$) ppm. The very poor solubility of the compound precluded generation of sufficient ^{13}C NMR data. **^{19}F NMR** (C_6D_5F, 565 MHz, 298 K): δ −166.7 (t, 8F, *meta* aryl−*F*), −162.9 (t, 4F, *para* aryl−*F*), −131.6 (d, 8F, *ortho* aryl−*F*) ppm. **Elemental analysis** Found: C 64.10; H, 5.84; N, 3.28. Calc. for $C_{88}H_{94}AlBF_{20}MgN_4$ (M = 1649.81 g/mol): C, 64.07; H, 5.74; N, 3.40%.

[(^{tBu}BDI)Mg-Ga(^{Me}BDI)$^+$][B(C_6F_5)$_4^-$]:

$[(^{tBu}BDI)Mg{\cdot}benzene^+]$ $[B(C_6F_5)_4^-]$ (28.5 mg, 22.2 µmol, 1 eq.) was dissolved in fluorobenzene (0.5 mL). $(^{Me}BDI)Ga$ (10.8 mg, 22.2 µmol, 1 eq.) was added and the reaction mixture was stirred for 5 minutes. The off-white precipitate that formed was isolated by removing the mother liquor. Washing with hexane (3 x 2 mL) gave 21 mg of $[(^{tBu}BDI)Mg\text{-}Ga(^{Me}BDI)^+][B(C_6F_5)_4^-]$ (56% yield). **^{1}H NMR** (C_6D_5F, 600 MHz, 298 K): δ 7.76 ($t_{(superimposed)}$, 2H, aryl−*H*), 7.63 (d, $^3J_{HH}$ = 7.7 Hz, 4H, aryl−*H*), other aryl−*H* obscured by C_6D_5F, 6.43 (s, 1H, $^{tBu}CCHC$), 5.86 (s, 1H, $^{Me}CCHC$), 3.70 (sept, $^3J_{HH}$ = 6.9 Hz, 4H, $CH(CH_3)_2$), 3.62 (br. sept, 4H, $CH(CH_3)_2$), 2.36 (s, 6H, ^{Me}BDI), 1.86 (d, $^3J_{HH}$ = 6.9 Hz, 12H, $CH(CH_3)_2$), 1.77 (2x $d_{(superimposed)}$, 24H, $CH(CH_3)_2$), 1.74 (s, 18H, ^{tBu}BDI), 1.48 (d, $^3J_{HH}$ = 6.8 Hz, 12H, $CH(CH_3)_2$). **^{11}B NMR** (C_6D_5F, 193 MHz, 298 K): δ −15.4 (s, 1B, $B(C_6F_5)_4^-$) ppm. **^{13}C NMR** (C_6D_5F, 151 MHz, 298 K): δ 182.1 (s, $^{tBu}CCHC$), 164.0 (s, $^{Me}CCHC$), 144.4 (s, aryl−*C*), 144.0 (s, aryl−*C*), 143.3 (s, aryl−*C*), 142.2 (s, aryl−*C*), 126.9 (s, aryl−*C*), 126.7 (s, aryl−*C*), 125.0 (s, aryl−*C*), 124.1 (s, aryl−*C*), 99.8 (s, $^{Me}CCHC$), 97.0 (s, $^{tBu}CCHC$), 45.3 (s, ^{tBu}BDI), 32.6 (s, ^{tBu}BDI), 28.8 (s, $CH(CH_3)_2$), 28.5 (s, $CH(CH_3)_2$), 26.0 (s, $CH(CH_3)_2$), 25.5 (s, $CH(CH_3)_2$), 23.8 (s, ^{Me}BDI), 23.6 (s, $CH(CH_3)_2$), 23.4 (s, $CH(CH_3)_2$) ppm. Signals for the $B(C_6F_5)_4$ were not detected due to very poor solubility of the compound; assignment was done by 2D NMR techniques. **^{19}F NMR** (C_6D_5F, 565 MHz, 298 K): δ −166.5 (t, 8F, *meta* aryl−*F*), −162.7 (t, 4F, *para* aryl−*F*), −131.5 (d, 8F, *ortho* aryl−*F*) ppm. **Elemental analysis** Found: C 63.05; H, 5.72; N, 3.31. Calc. for $C_{88}H_{94}BF_{20}GaN_4Zn$ (M = 1692.55 g/mol) one PhF cocrystallized: C, 63.12; H, 5.58; N, 3.13%.

$[(^{tBu}BDI)Zn\text{-}Ga(^{Me}BDI)^+][B(C_6F_5)_4^-]$:

$[(^{tBu}BDI)Zn{\cdot}benzene^+][B(C_6F_5)_4^-]$ (38.3 mg, 28.9 µmol, 1 eq.) was dissolved in fluorobenzene (0.5 mL) and $(^{Me}BDI)Ga$ (14.1 mg, 28.9 µmol, 1 eq.) was added. The reaction mixture was stirred for 5 minutes and subsequently layered with hexane (0.5 mL). An off-white precipitate formed which was isolated by removing the mother liquor. Washing with hexane (3 x 2 mL) gave the product $[(^{tBu}BDI)Zn\text{-}Ga(^{Me}BDI)^+][B(C_6F_5)_4^-]$ in 62% yield (31 mg). Crystals suitable for X−ray diffraction were grown from the reaction mixture layered with hexane. **^{1}H NMR** (C_6D_5Br, 600 MHz, 298 K): δ 7.23 (t, $^3J_{HH}$ = 7.8 Hz, 1H, aryl−*H*), 7.11 (m, 3H, aryl−*H*), 7.05 (m, 1H, aryl−*H*), 6.97 (m, 2H, aryl−*H*), 6.90 (m, 3H, aryl−*H*), 6.73 (d, $^3J_{HH}$ = 7.6 Hz, 1H, aryl−*H*), 6.70 (d, $^3J_{HH}$ = 7.7 Hz, 1H, aryl−*H*), 5.88 (s, 1H, $CCHC$), 5.42 (s, 1H, $CCHC$), 3.50 (sept, $^3J_{HH}$ = 6.4 Hz, 1H, $CH(CH_3)_2$), 3.06 (sept, $^3J_{HH}$ = 7.1 Hz, 1H, $CH(CH_3)_2$), 2.85 (sept, $^3J_{HH}$ = 6.9 Hz, 1H, $CH(CH_3)_2$), 2.55 (sept, $^3J_{HH}$ = 7.0 Hz, 1H, $CH(CH_3)_2$), 2.47 (sept, $^3J_{HH}$ = 6.8Hz, 1H, $CH(CH_3)_2$), 2.14 (2 x sept, 2H, $CH(CH_3)_2$), 2.03 (sept, $^3J_{HH}$ = 6.6 Hz, 1H, $CH(CH_3)_2$), 1.49 (s, 3H, ^{Me}BDI), 1.41 (s, 3H, ^{Me}BDI), 1.32 (d, $^3J_{HH}$ = 6.5 Hz, 3H, $CH(CH_3)_2$), 1.22 (d, $^3J_{HH}$ = 6.7 Hz, 3H, $CH(CH_3)_2$), 1.19 (2 x d, 6H, $CH(CH_3)_2$), 1.02 (d, $^3J_{HH}$ = 6.8 Hz, 3H, $CH(CH_3)_2$), 0.97 (s, 9H, ^{tBu}BDI), 0.97 ($d_{(superimposed)}$, 3H, $CH(CH_3)_2$), 0 93 (m, 9H, $CH(CH_3)_2$), 0.93 (s, 9H, ^{tBu}BDI), 0.83 (d, $^3J_{HH}$ = 6.7 Hz, 3H, $CH(CH_3)_2$), 0.81 (d, $^3J_{HH}$ = 6.5 Hz, 6H, $CH(CH_3)_2$), 0.57 (d, $^3J_{HH}$ = 6.8 Hz, 3H, $CH(CH_3)_2$), 0.04 (d, $^3J_{HH}$ = 6.8 Hz, 3H, $CH(CH_3)_2$), −0.10 (d, $^3J_{HH}$ = 6.8 Hz, 6H, $CH(CH_3)_2$)

ppm. **^{11}B NMR** (C_6D_5Br, 193 MHz, 298 K): δ −15.5 (s, 1B, *B*(C_6F_5)$_4^-$) ppm. **^{13}C NMR** (C_6D_5Br, 101 MHz, 298 K): δ 180.9 (s, *C*CH*C*), 179.9 (s, *C*CH*C*), 171.5 (s, *C*CH*C*), 171.3 (s, *C*CH*C*), 145.5 (s, aryl−*C*), 144.9 (s, aryl−*C*), 144.5 (s, aryl−*C*), 143.9 (s, aryl−*C*), 141.6 (s, aryl−*C*), 139.4 (s, aryl−*C*), 139.0 (s, aryl−*C*), 138.7 (s, aryl−*C*), 130.0 (s, aryl−*C*), 126.6 (s, aryl−*C*), 126.2 (s, aryl−*C*), 125.7 (s, aryl−*C*), 125.5 (s, aryl−*C*), 124.6 (s, aryl−*C*), 124.2 (s, aryl−*C*), 123.7 (s, aryl−*C*), 104.6 (s, C*C*HC), 101.2 (s, C*C*HC), 45.0 (s, ^{tBu}BDI), 44.7 (s, ^{tBu}BDI), 33.7 (s, ^{tBu}BDI), 32.8 (s, ^{tBu}BDI), 24.9 (s, ^{Me}BDI), 24.4 (s, ^{Me}BDI), 29.7 (s, *C*H(CH_3)$_2$), 29.2 (s, *C*H(CH_3)$_2$), 28.6 (s, *C*H(CH_3)$_2$), 33.2 (s, CH(CH_3)$_2$), 32.7 (s, CH(*C*H_3)$_2$), 28.6 (s, CH(*C*H_3)$_2$), 28.1 (s, CH(*C*H_3)$_2$), 28.0 (s, CH(*C*H_3)$_2$), 25.6 (s, CH(*C*H_3)$_2$), 24.2 (s, CH(*C*H_3)$_2$), 23.2 (s, CH(*C*H_3)$_2$), 23.1 (s, CH(*C*H_3)$_2$), 22.1 (s, CH(*C*H_3)$_2$) ppm. Some signals for the aromatic and aliphatic proton and B(*C*$_6F_5$)$_4$ were not detected due to very limited solubility of the compound; assignment was done by 2D NMR techniques. **^{19}F NMR** (C_6D_5Br, 565 MHz, 298 K): δ −165.4 (t, 8F, *meta* aryl−*F*), −161.6 (t, 4F, *para* aryl−*F*), −131.0 (d, 8F, *ortho* aryl−*F*) ppm. **Elemental analysis** Found: C 61.55; H, 5.45; N, 3.27. Calc. for $C_{88}H_{94}BF_{20}GaN_4Zn$ (M = 1733.63 g/mol): C, 60.97; H, 5.47; N, 3.23%.

2. Selected NMR spectra

2.1 Spectra of [(^{tBu}BDI)Mg-Al(^{Me}BDI)$^+$][B(C_6F_5)$_4^-$]

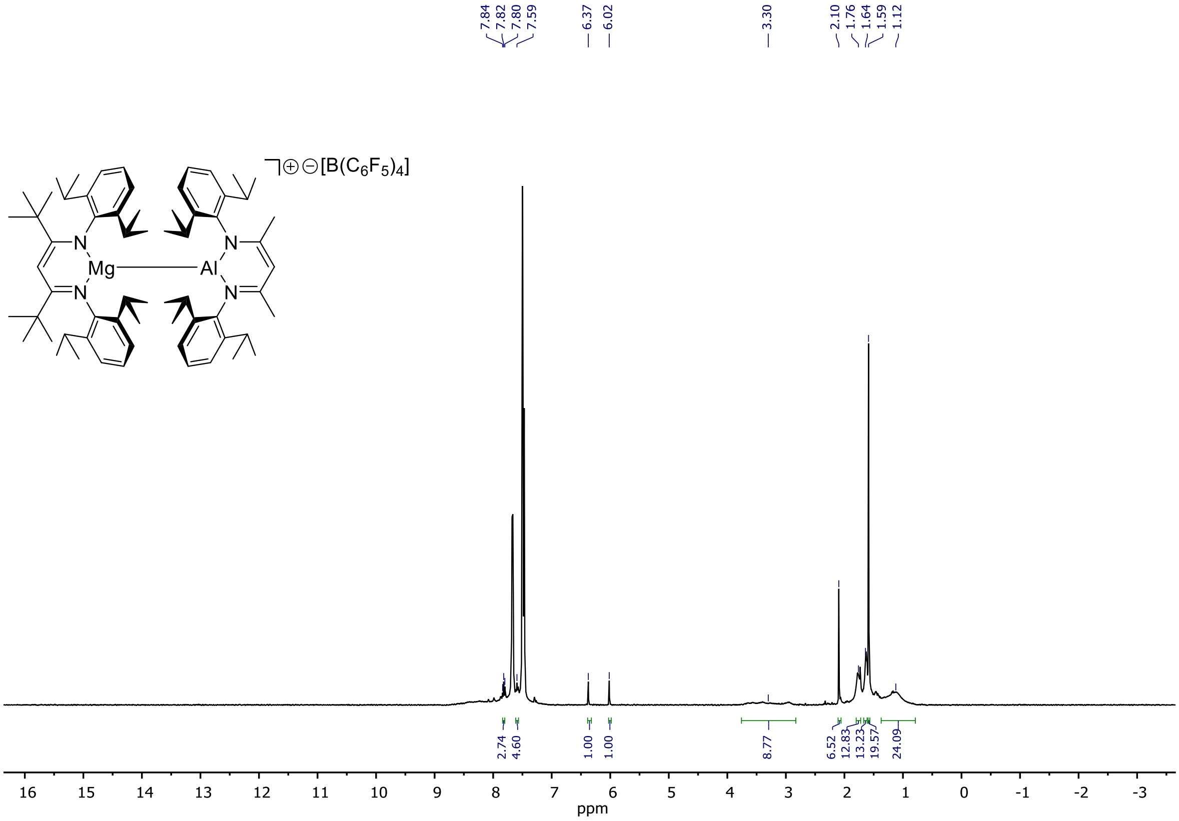

Figure S1: ^{1}H NMR (400 MHz, 298 K) spectrum of [(^{tBu}BDI)Mg-Al(^{Me}BDI)$^+$][B(C_6F_5)$_4^-$] in C_6D_5F.

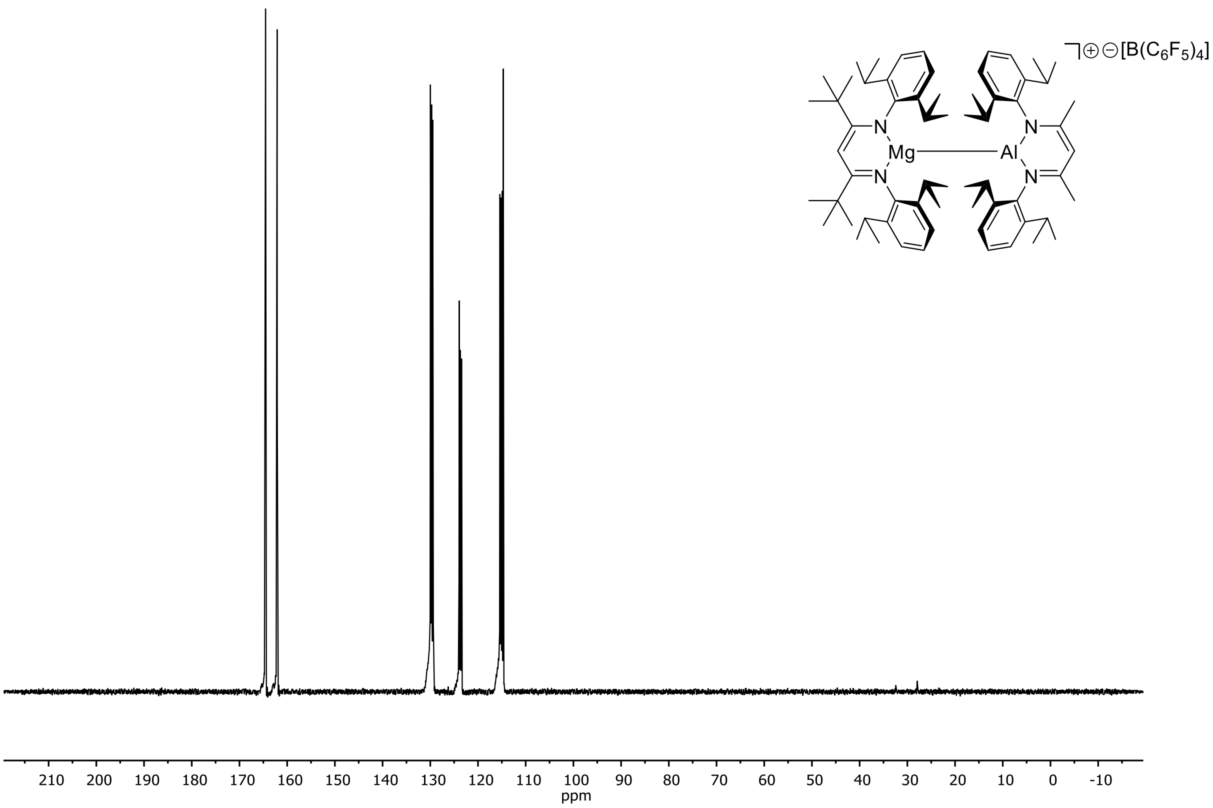

Figure S2: ^{13}C NMR (101 MHz, 298 K) spectrum of [(^{tBu}BDI)Mg-Al(^{Me}BDI)$^+$][B(C_6F_5)$_4^-$] in C_6D_5F.

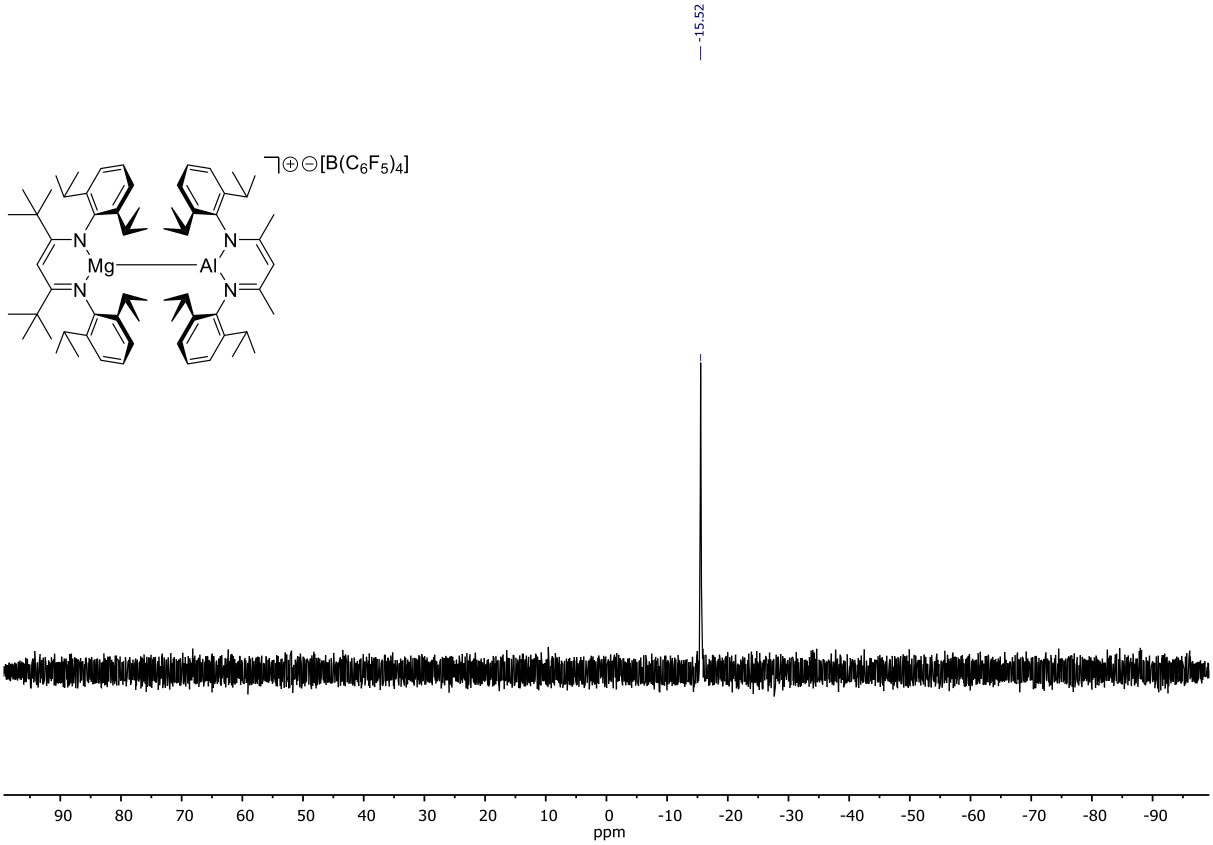

Figure S3: ^{11}B NMR (128 MHz, 298 K) spectrum of [(^{tBu}BDI)Mg-Al(^{Me}BDI)$^+$][B(C_6F_5)$_4^-$] in C_6D_5F.

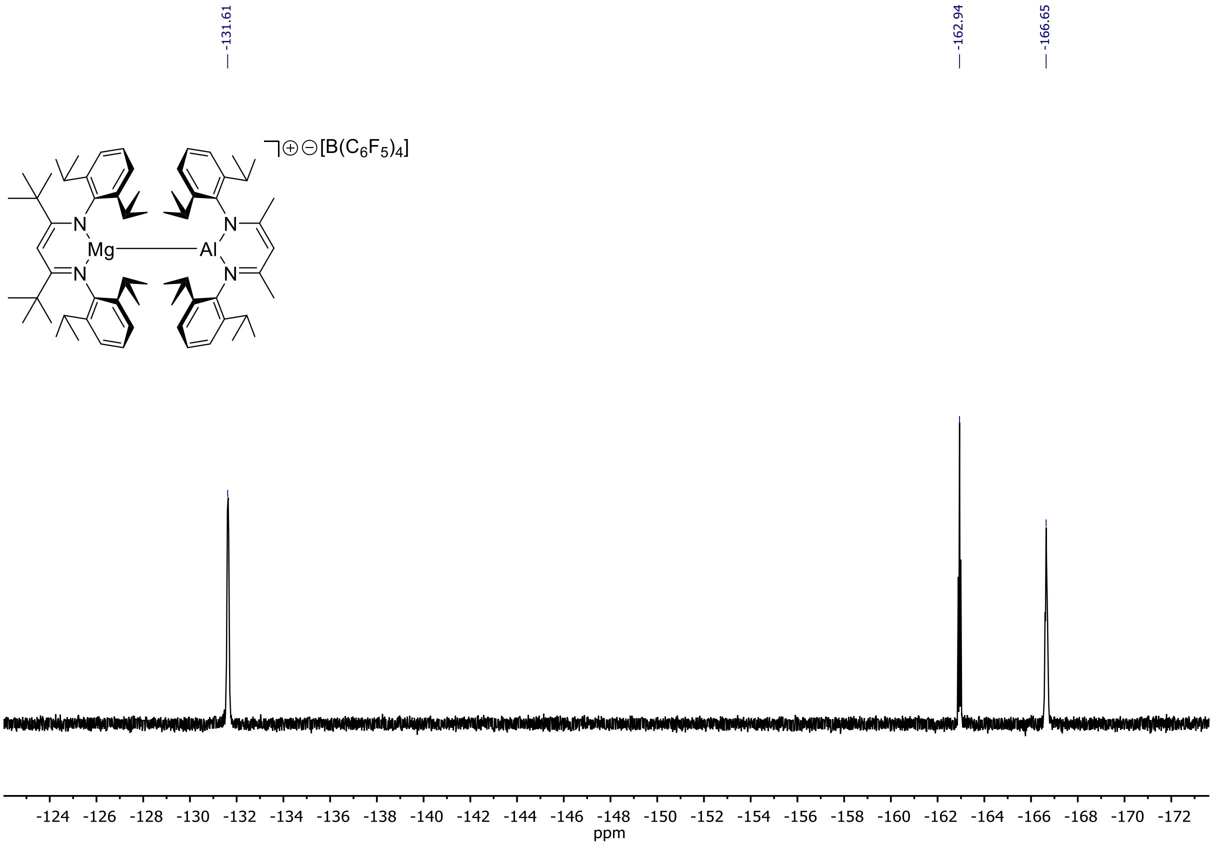

Figure S4: ^{19}F NMR (376 MHz, 298 K) spectrum of [(^{tBu}BDI)Mg-Al(^{Me}BDI)$^{+}$][B(C_6F_5)$_4^{-}$] in C_6D_5F.

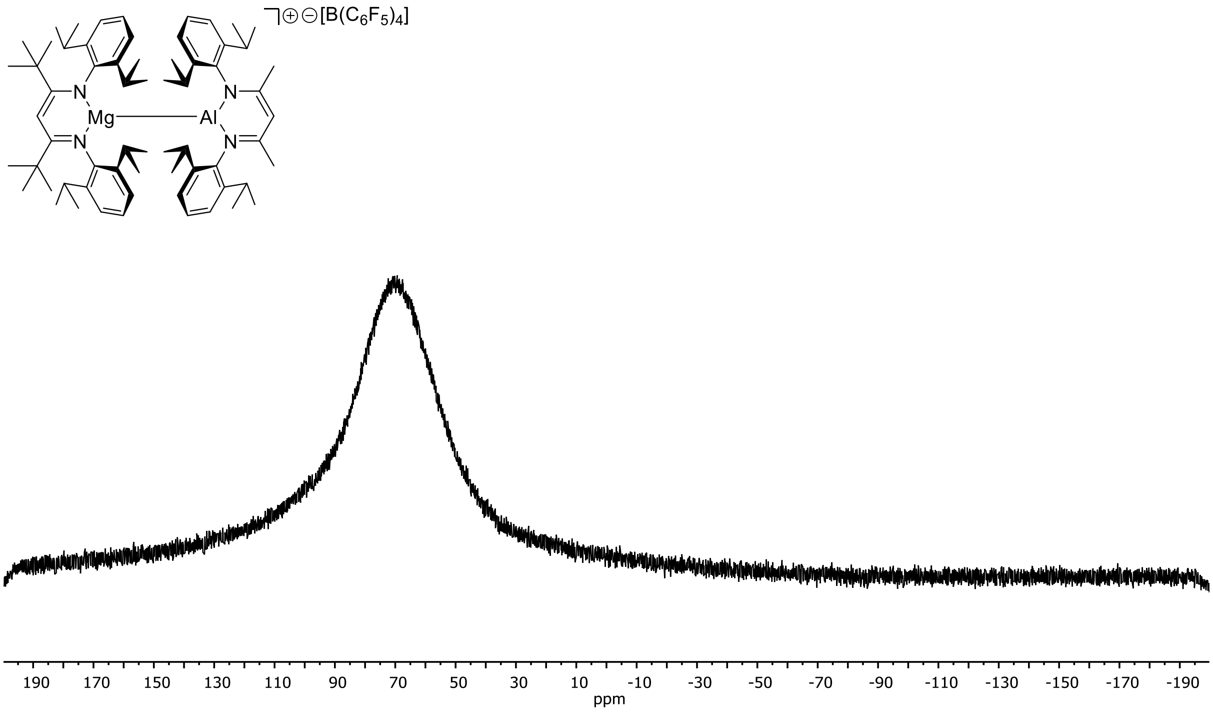

Figure S5: ^{27}Al NMR (162 MHz, 298 K) spectrum of [(^{tBu}BDI)Mg-Al(^{Me}BDI)$^{+}$][B(C_6F_5)$_4^{-}$] in C_6D_5F.

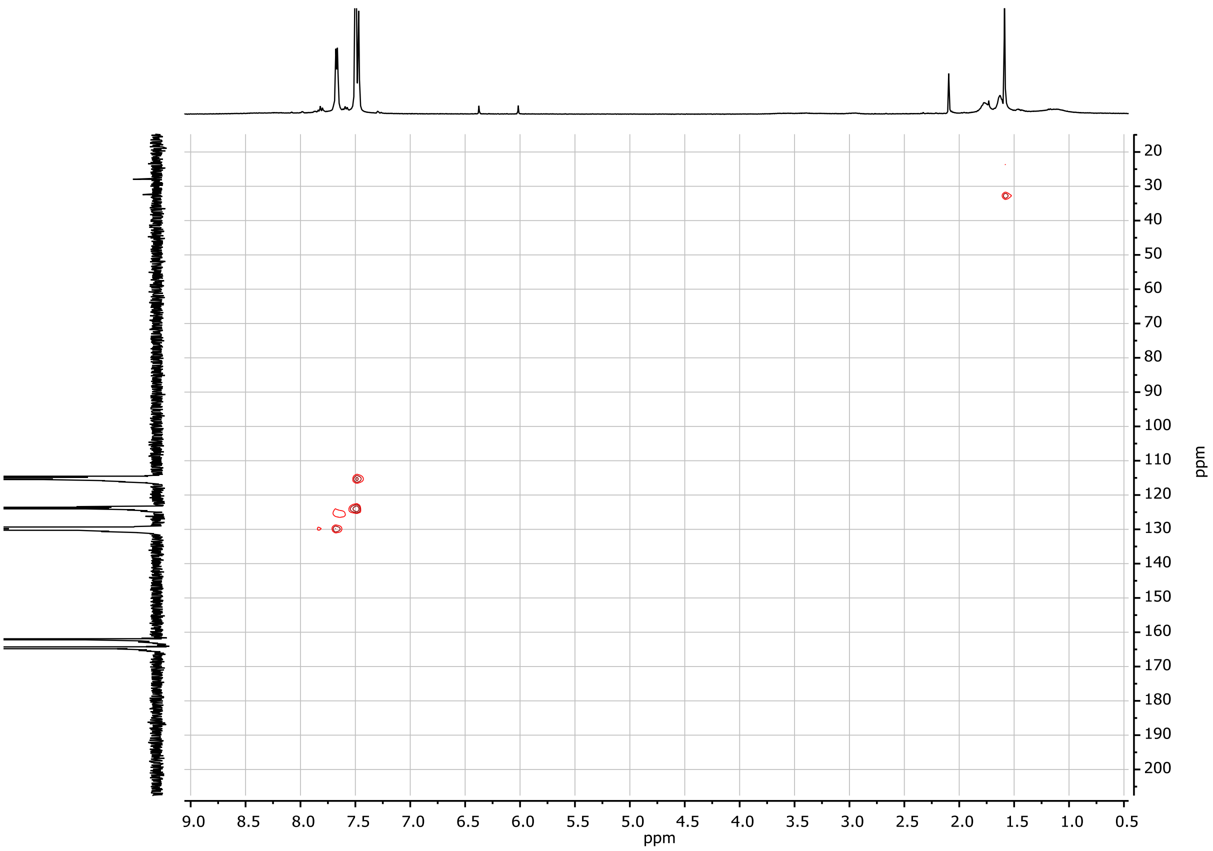

Figure S6: 2D HSQC NMR spectrum of [(^{tBu}BDI)Mg-Al(^{Me}BDI)$^{+}$][B(C_6F_5)$_4^{-}$] in C_6D_5F.

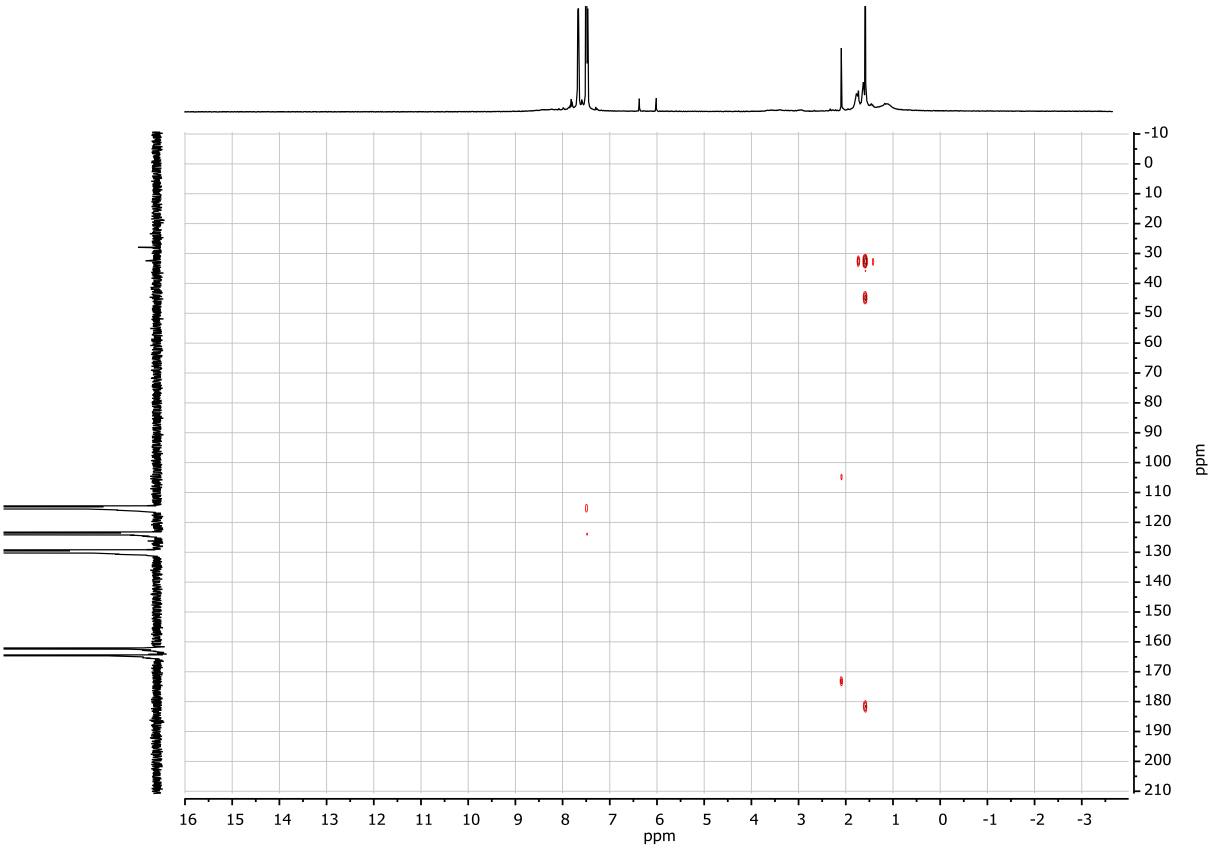

Figure S7: 2D HMBC NMR spectrum of [(^{tBu}BDI)Mg-Al(^{Me}BDI)$^{+}$][B(C_6F_5)$_4^{-}$] in C_6D_5F.

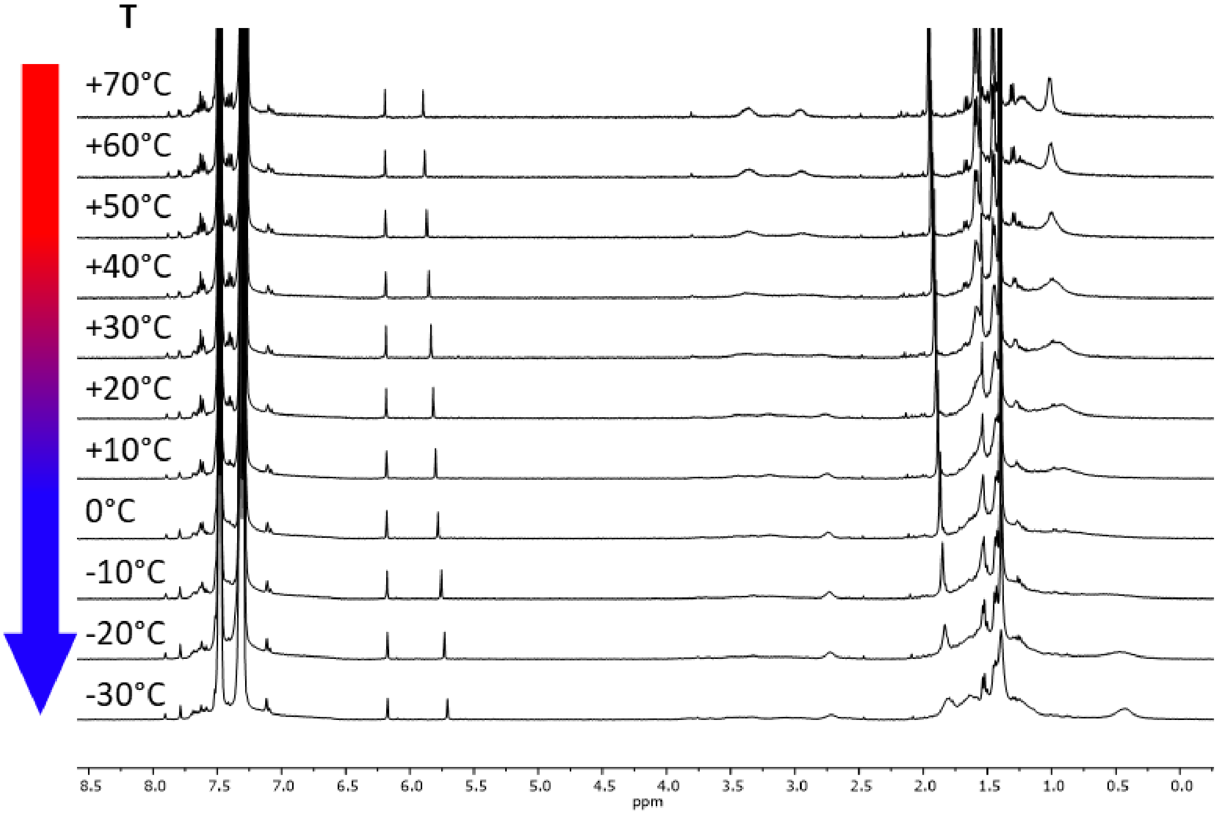

Figure S8: Temperature-dependent ^{1}H NMR spectrum (400 MHz) of $[(^{tBu}BDI)Mg\text{-}Al(^{Me}BDI)^{+}][B(C_6F_5)_4^{-}]$ in C_6D_5F.

2.2 Spectra of [(^{tBu}BDI)Mg-Ga(^{Me}BDI)$^{+}$][B(C_6F_5)$_4^{-}$]

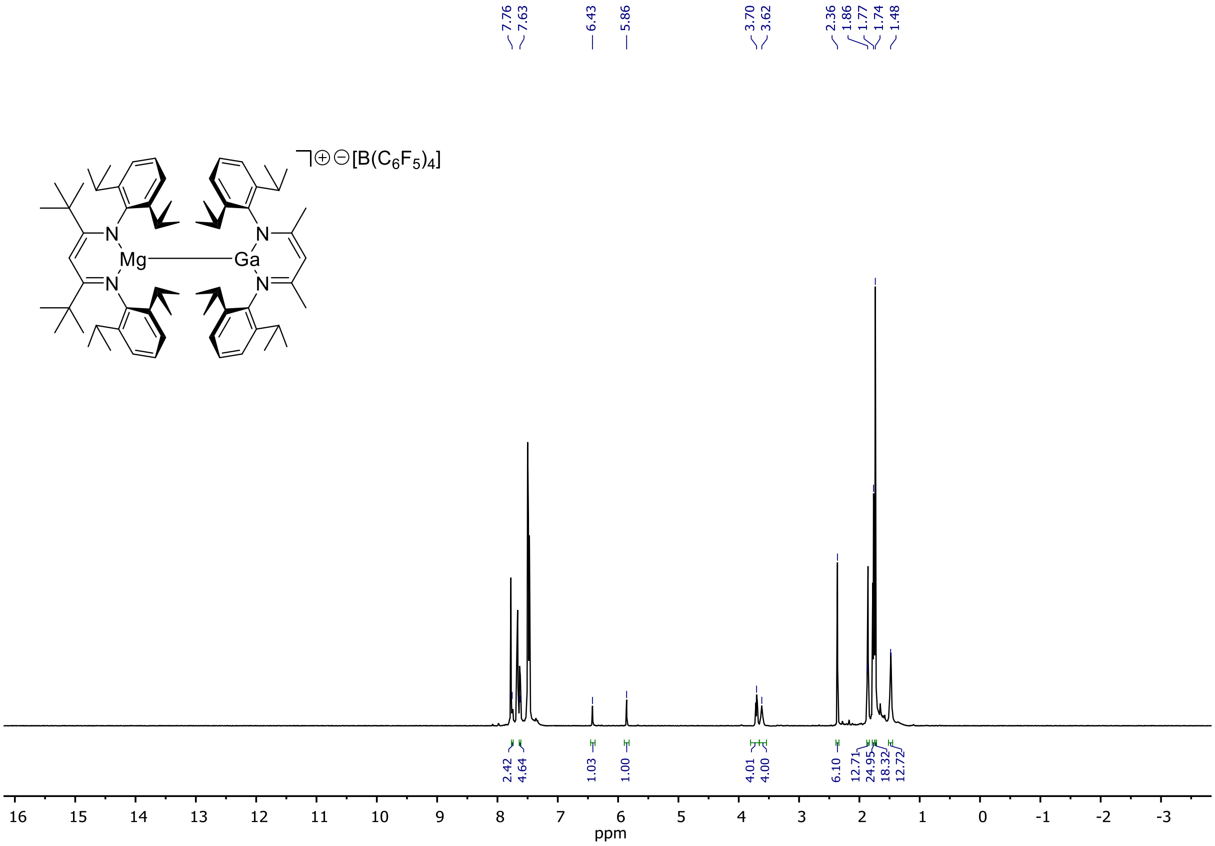

Figure S9: ^{1}H NMR (600 MHz, 298 K) spectrum of [(^{tBu}BDI)Mg-Ga(^{Me}BDI)$^{+}$][B(C_6F_5)$_4^{-}$] in C_6D_5F.

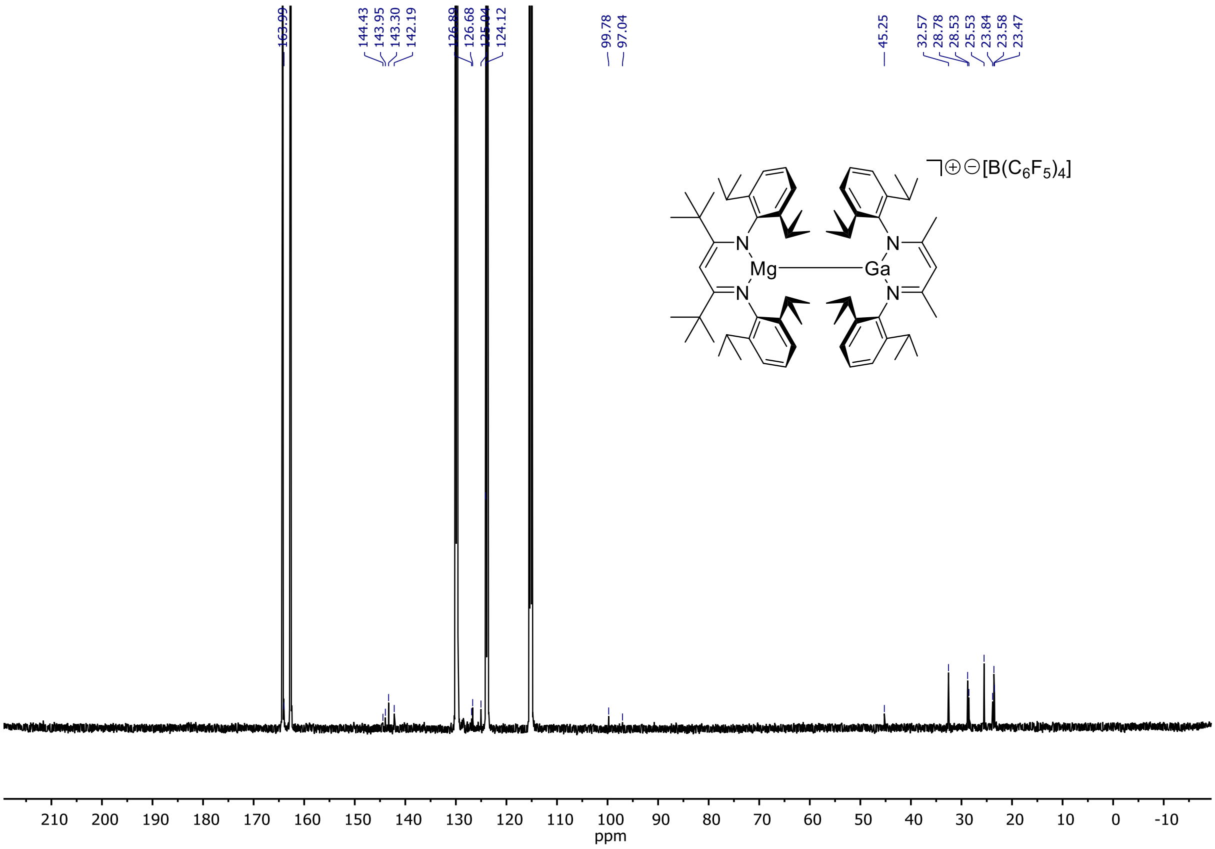

Figure S10: ^{13}C NMR (151 MHz, 298 K) spectrum of [(^{tBu}BDI)Mg-Ga(^{Me}BDI)$^{+}$][B(C_6F_5)$_4^{-}$] in C_6D_5F.

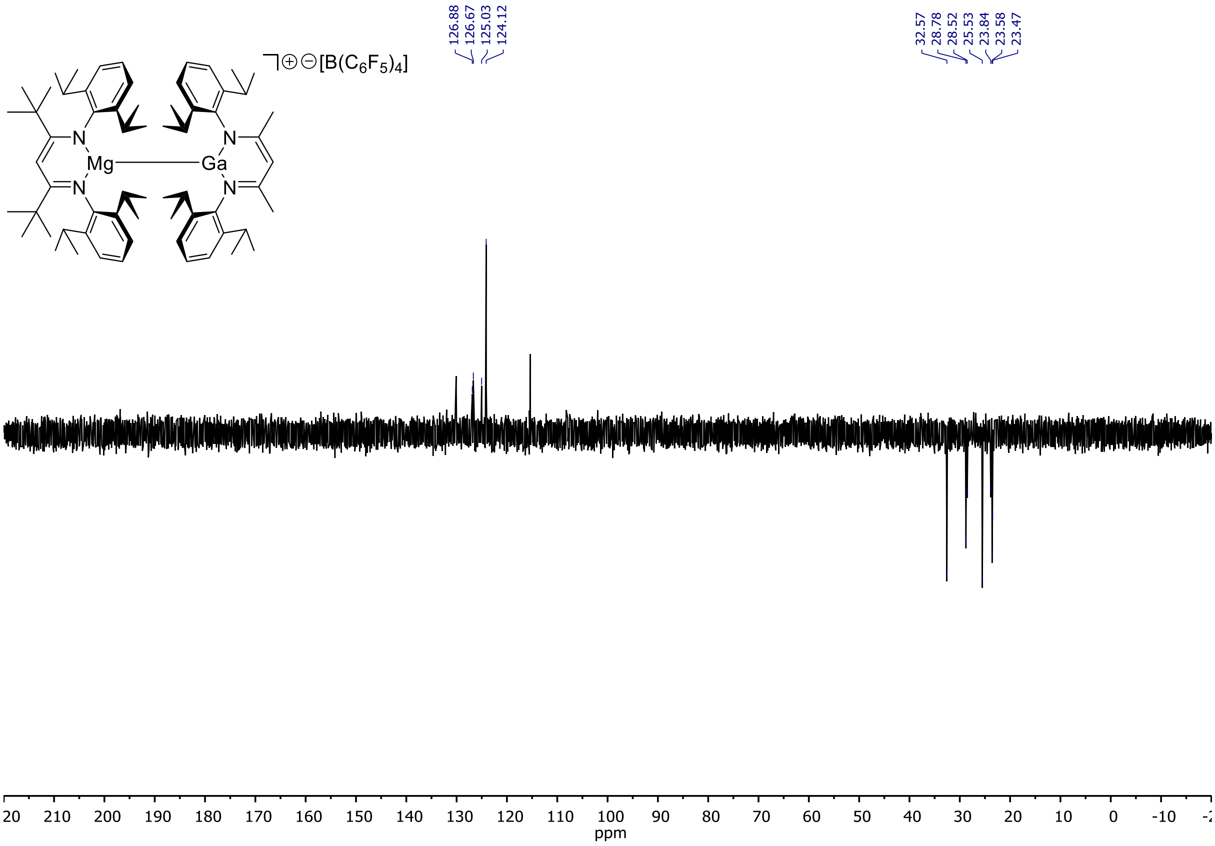

Figure S11: ^{13}C DEPT135 NMR (151 MHz, 298 K) spectrum of [(^{tBu}BDI)Mg-Ga(^{Me}BDI)$^{+}$][B(C_6F_5)$_4$$^{-}$] in C_6D_5F.

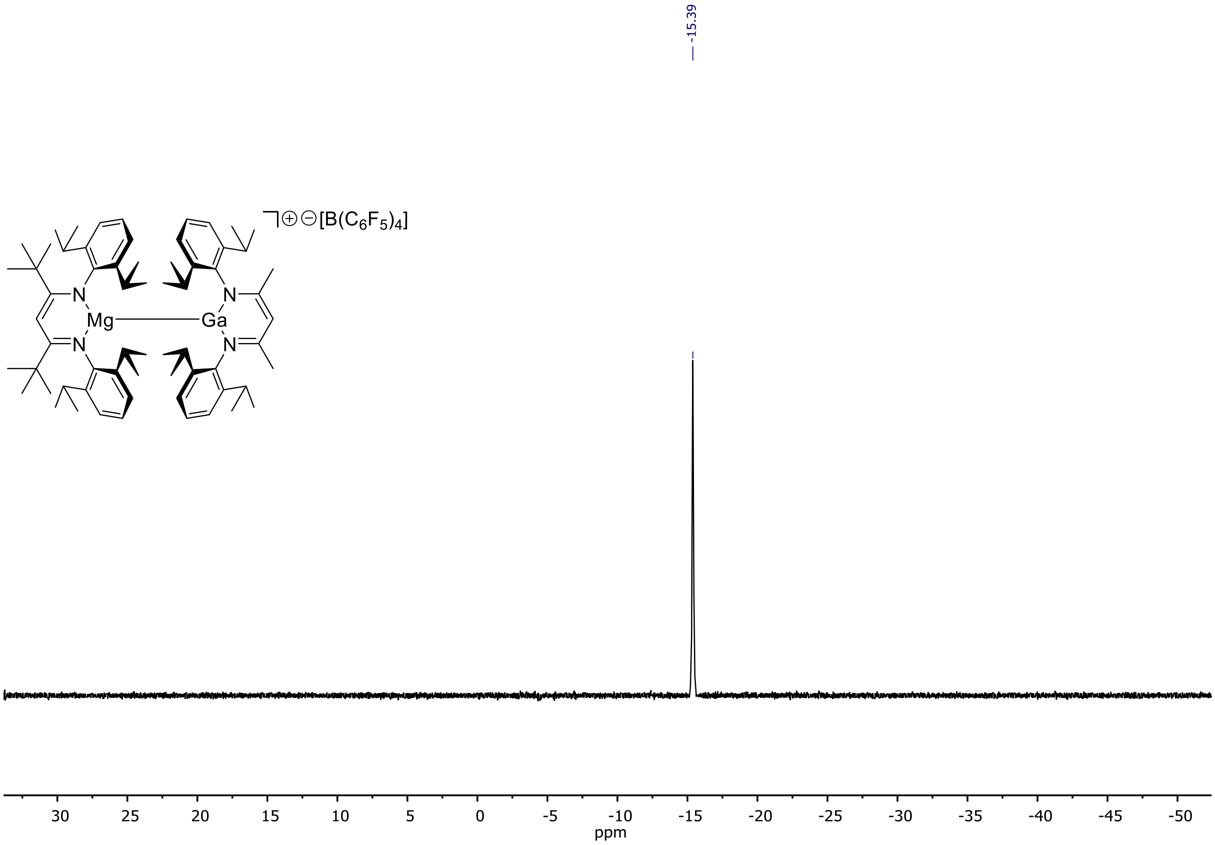

Figure S12: ^{11}B NMR (192 MHz, 298 K) spectrum of [(^{tBu}BDI)Mg-Ga(^{Me}BDI)$^{+}$][B(C_6F_5)$_4$$^{-}$] in C_6D_5F.

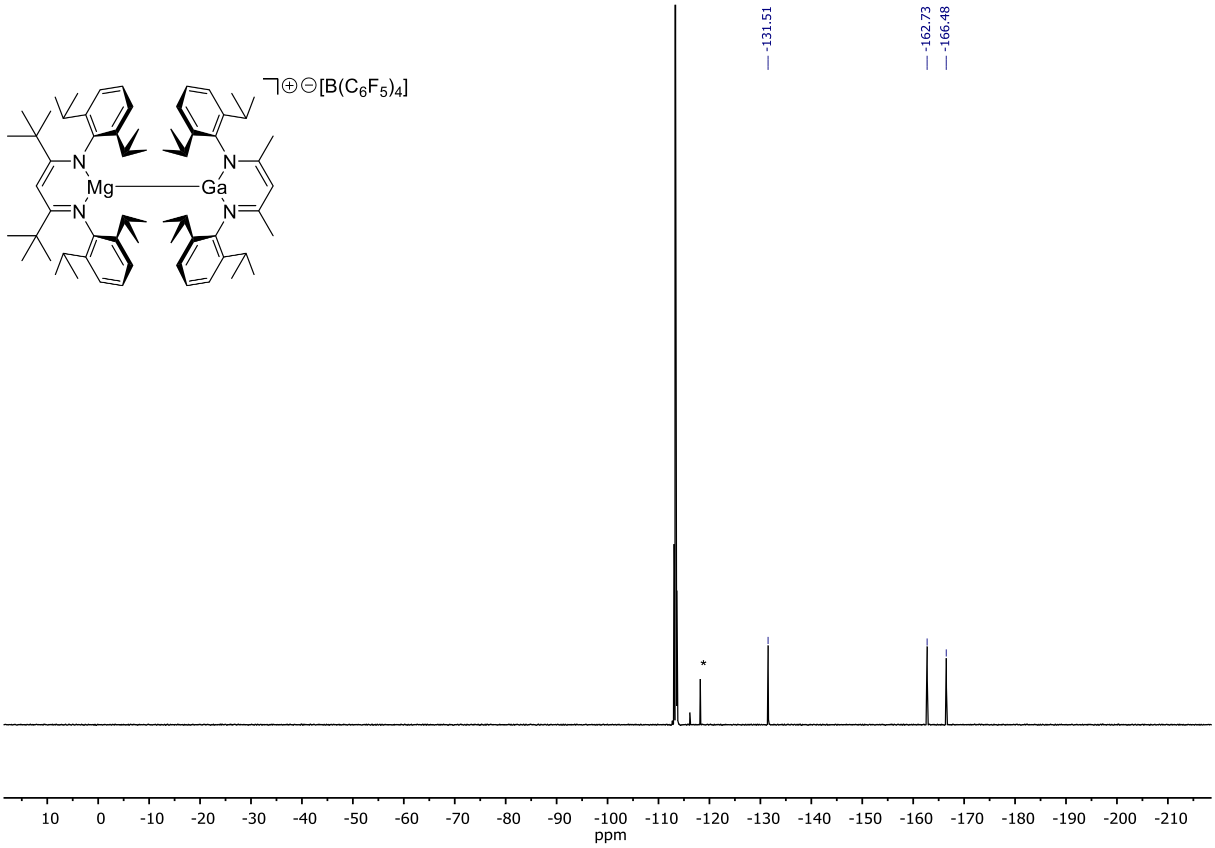

Figure S13: ^{19}F NMR (565 MHz, 298 K) spectrum of [(^{tBu}BDI)Mg-Ga(^{Me}BDI)$^+$][B(C_6F_5)$_4^-$] in C_6D_5F.

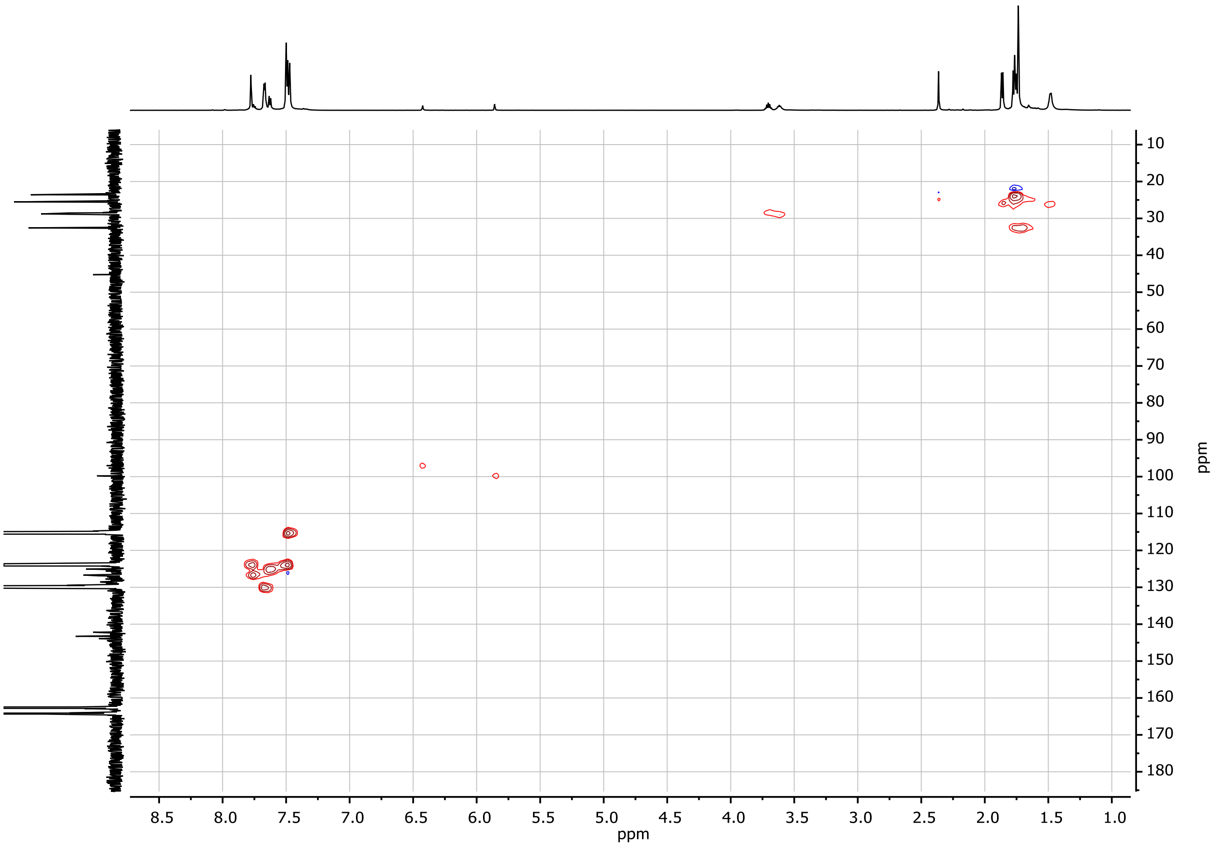

Figure S14: 2D HSQC NMR spectrum of [(^{tBu}BDI)Mg-Ga(^{Me}BDI)$^+$][B(C_6F_5)$_4^-$] in C_6D_5F.

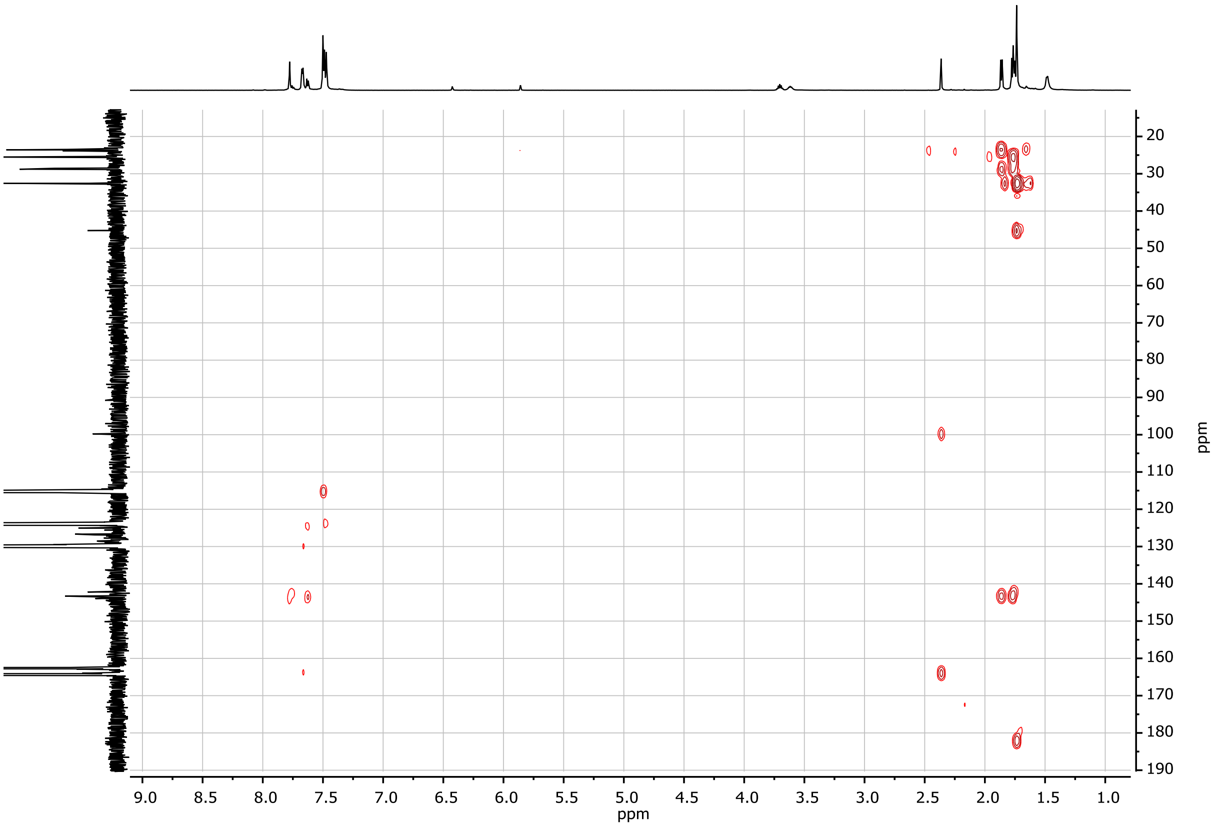

Figure S15: 2D HMBC NMR spectrum of [(^{tBu}BDI)Mg-Ga(^{Me}BDI)$^{+}$][B(C_6F_5)$_4^{-}$] in C_6D_5F.

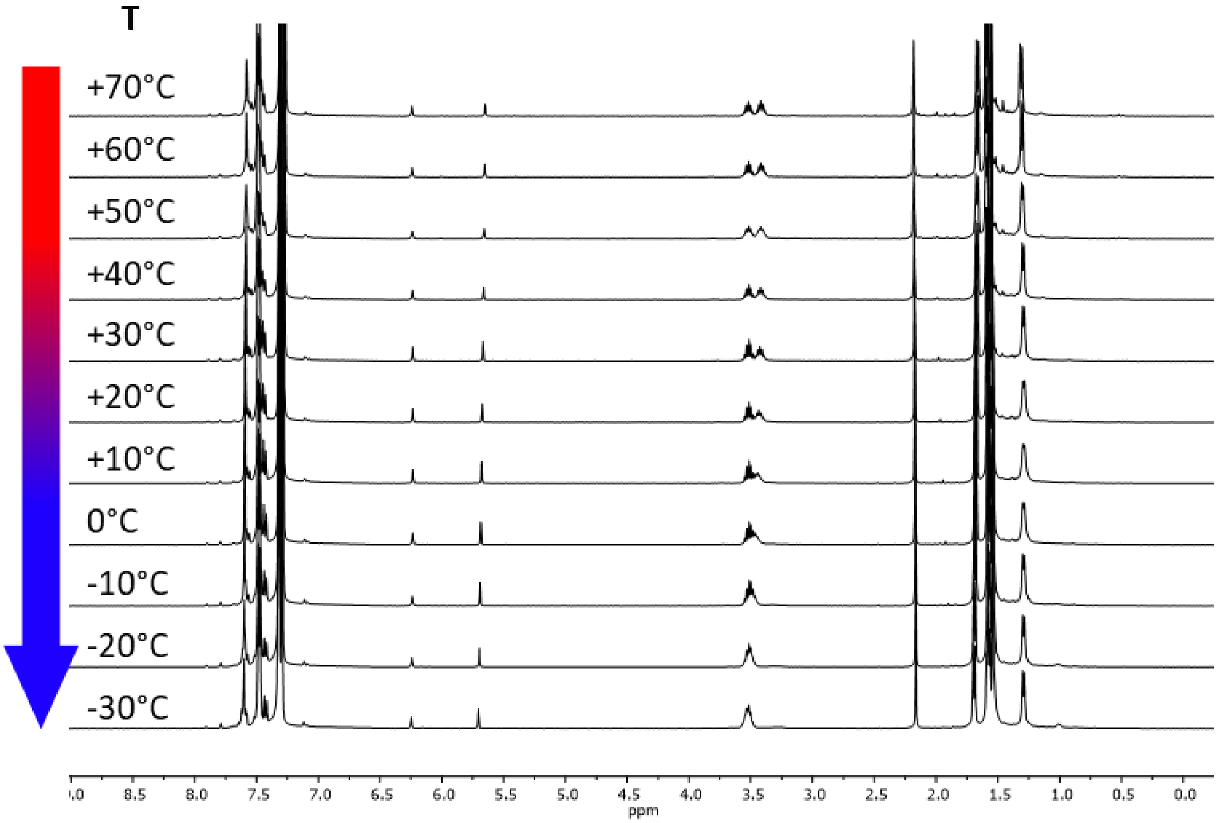

Figure S16: Temperature-dependent ^{1}H NMR (400 MHz) spectrum of [(^{tBu}BDI)Mg-Ga(^{Me}BDI)$^{+}$][B(C_6F_5)$_4^{-}$] in C_6D_5F.

2.3 Spectra of [(^{tBu}BDI)Zn-Ga(^{Me}BDI)$^{+}$][B(C_6F_5)$_4^{-}$]

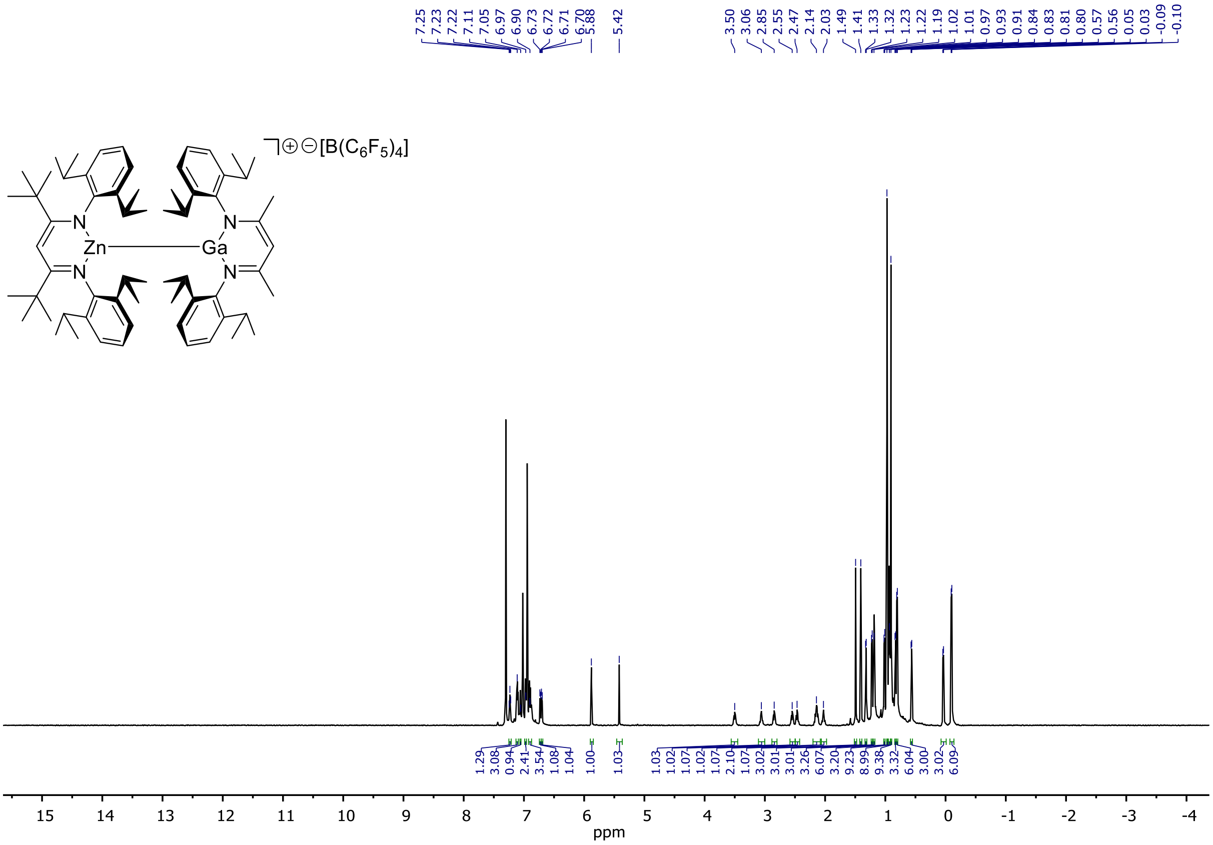

Figure S17: ^{1}H NMR (600 MHz, 298 K) spectrum of [(^{tBu}BDI)Zn-Ga(^{Me}BDI)$^{+}$][B(C_6F_5)$_4^{-}$] in C_6D_5Br.

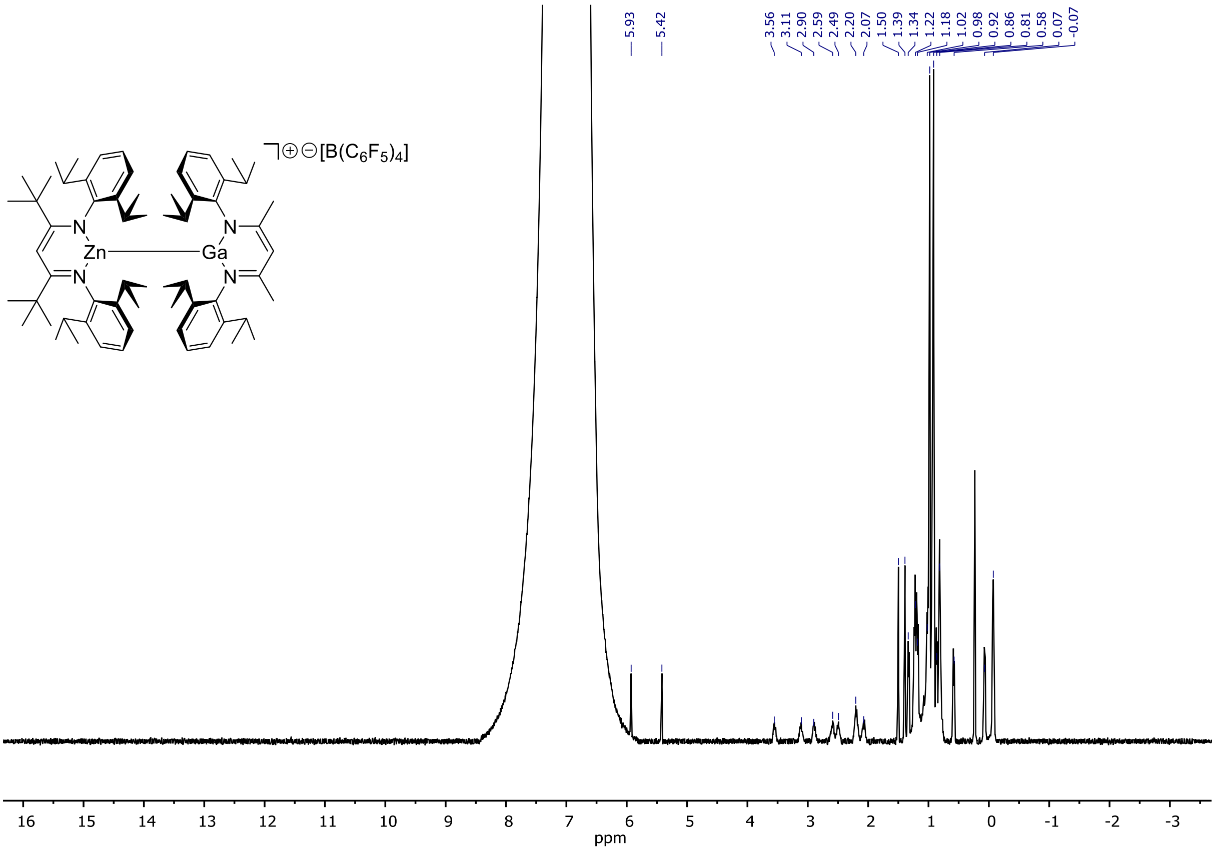

Figure S18: ^{1}H NMR (no-D, 400 MHz, 298 K) spectrum of [(^{tBu}BDI)Zn-Ga(^{Me}BDI)$^{+}$][B(C_6F_5)$_4^{-}$] in C_6H_5F.

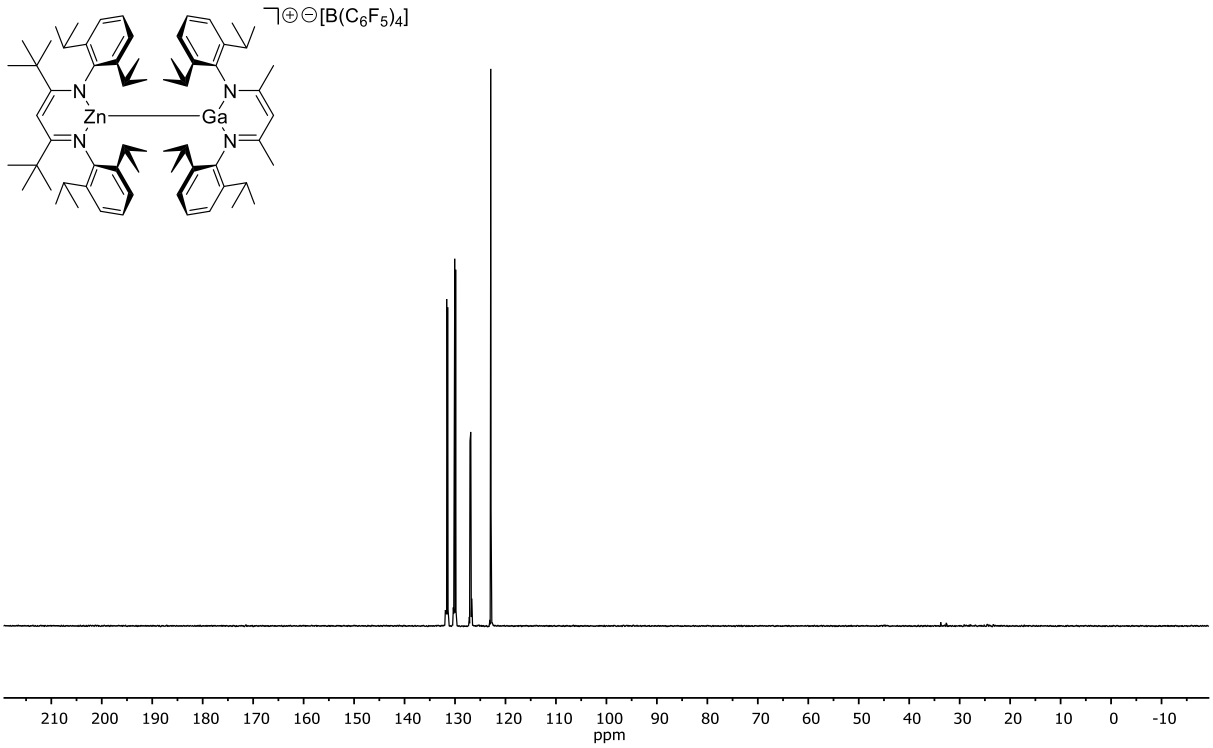

Figure S19: ^{13}C NMR (151 MHz, 298 K) spectrum of $[(^{tBu}BDI)Zn\text{-}Ga(^{Me}BDI)^{+}][B(C_6F_5)_4^{-}]$ in C_6D_5Br.

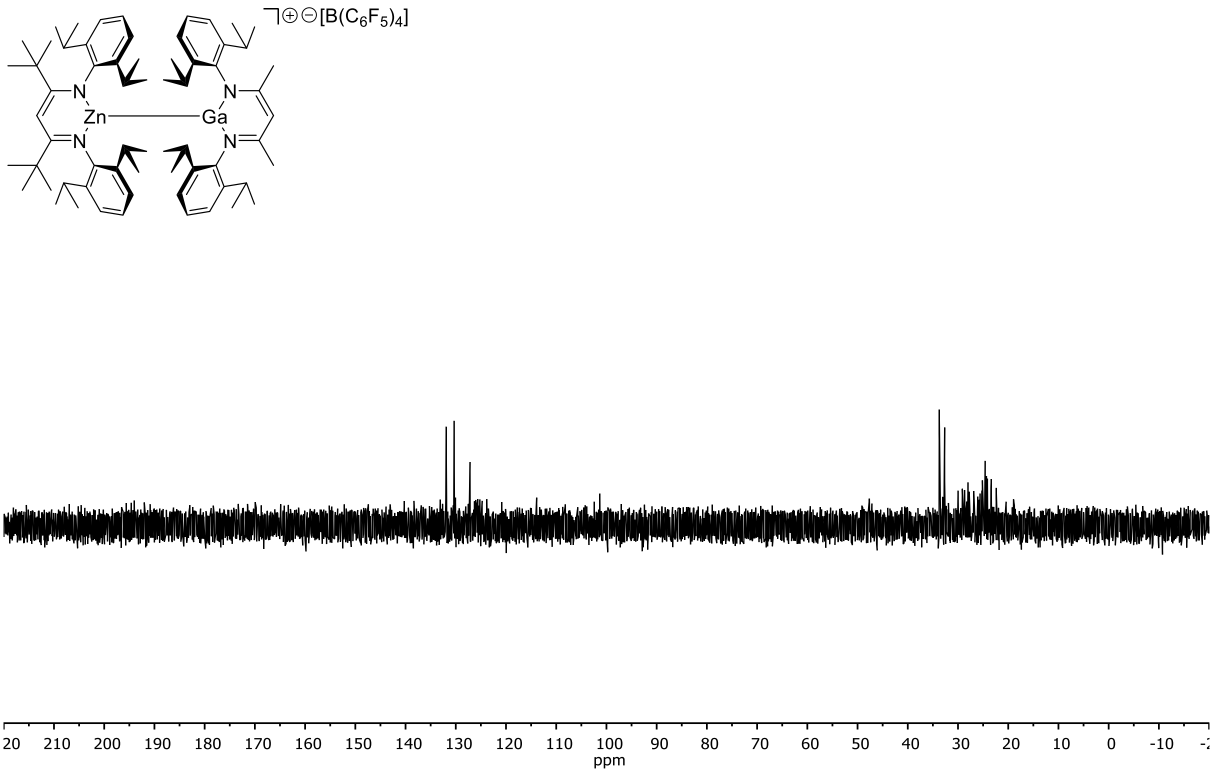

Figure S20: ^{13}C DEPT135 NMR (151 MHz, 298 K) spectrum of $[(^{tBu}BDI)Zn\text{-}Ga(^{Me}BDI)^{+}][B(C_6F_5)_4^{-}]$ in C_6D_5Br.

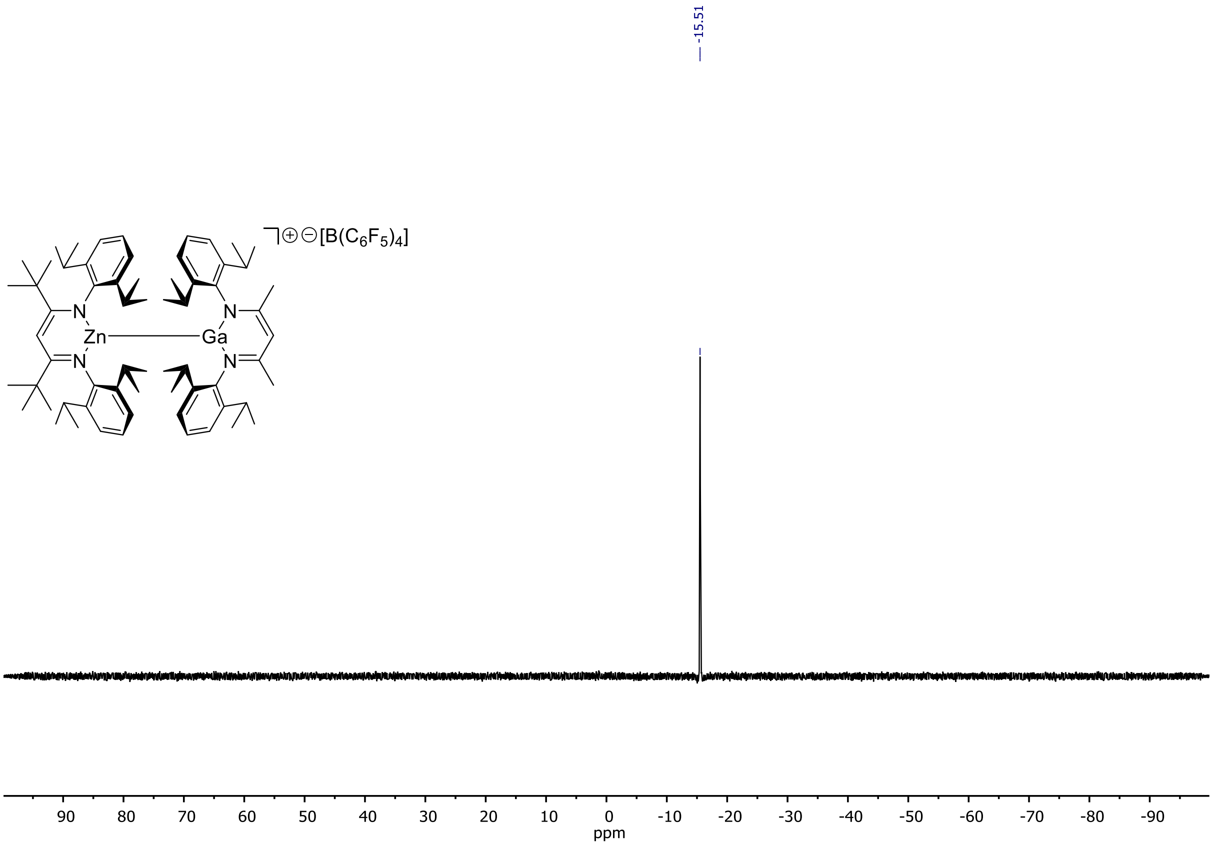

Figure S21: ^{11}B NMR (192 MHz, 298 K) spectrum of $[(^{tBu}BDI)Zn\text{-}Ga(^{Me}BDI)^{+}][B(C_6F_5)_4^{-}]$ in C_6D_5Br.

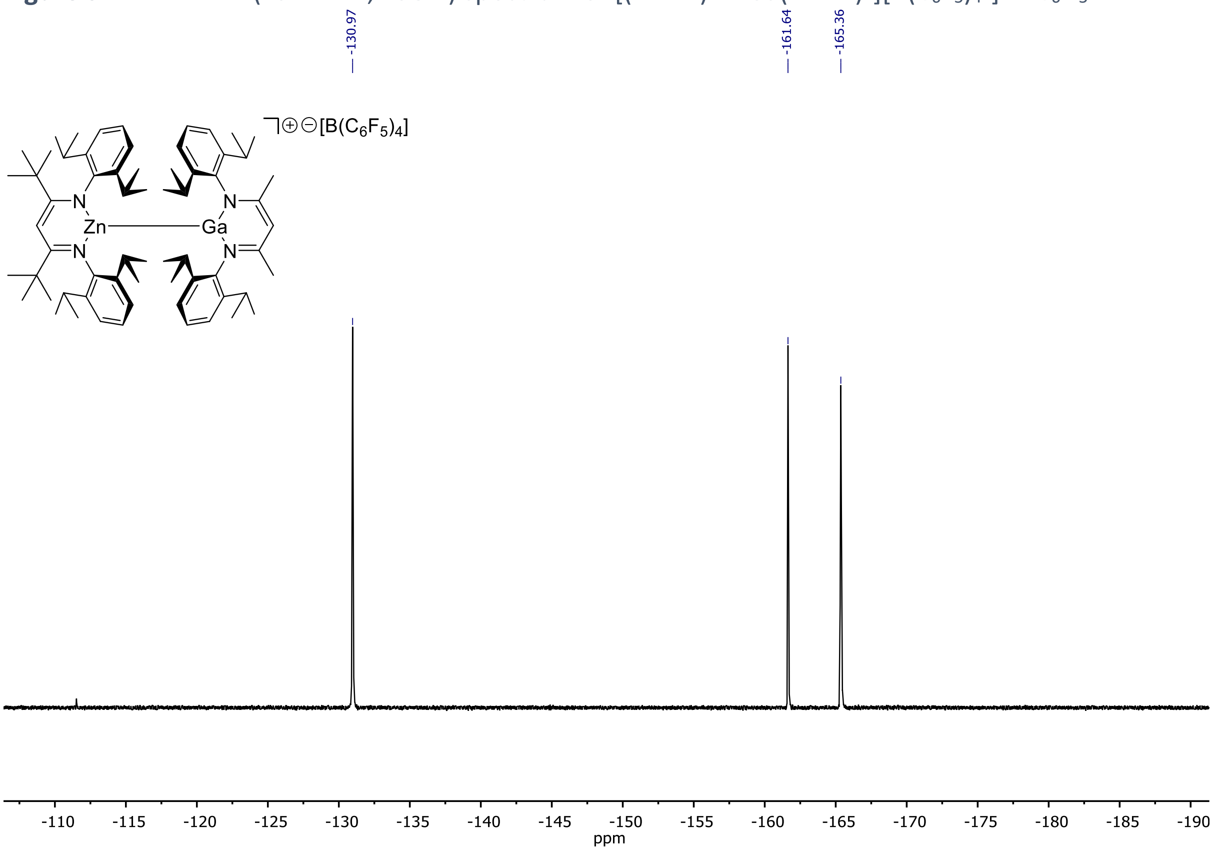

Figure S22: ^{19}F NMR (565 MHz, 298 K) spectrum of $[(^{tBu}BDI)Zn\text{-}Ga(^{Me}BDI)^{+}][B(C_6F_5)_4^{-}]$ in C_6D_5Br.

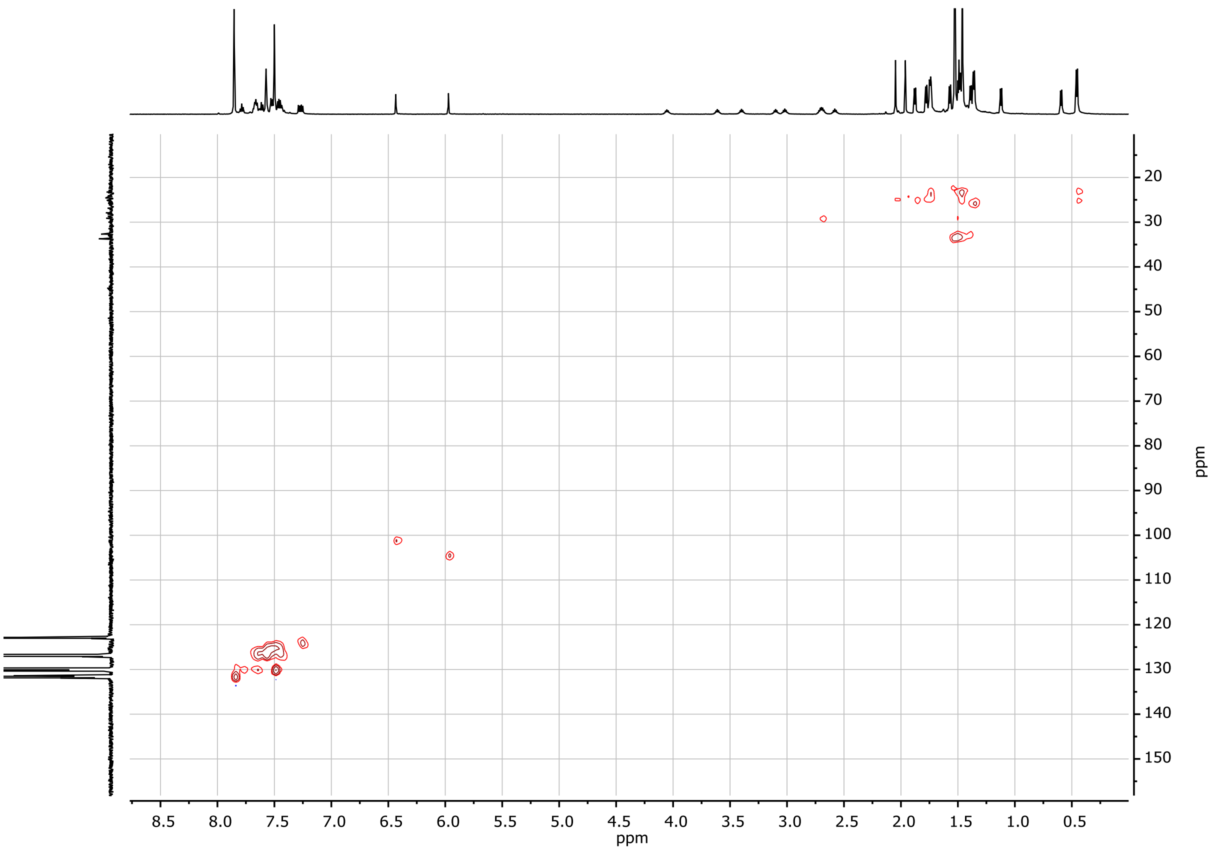

Figure S23: 2D HSQC NMR spectrum of $[(^{tBu}BDI)Zn\text{-}Ga(^{Me}BDI)^{+}][B(C_6F_5)_4^{-}]$ in C_6D_5Br.

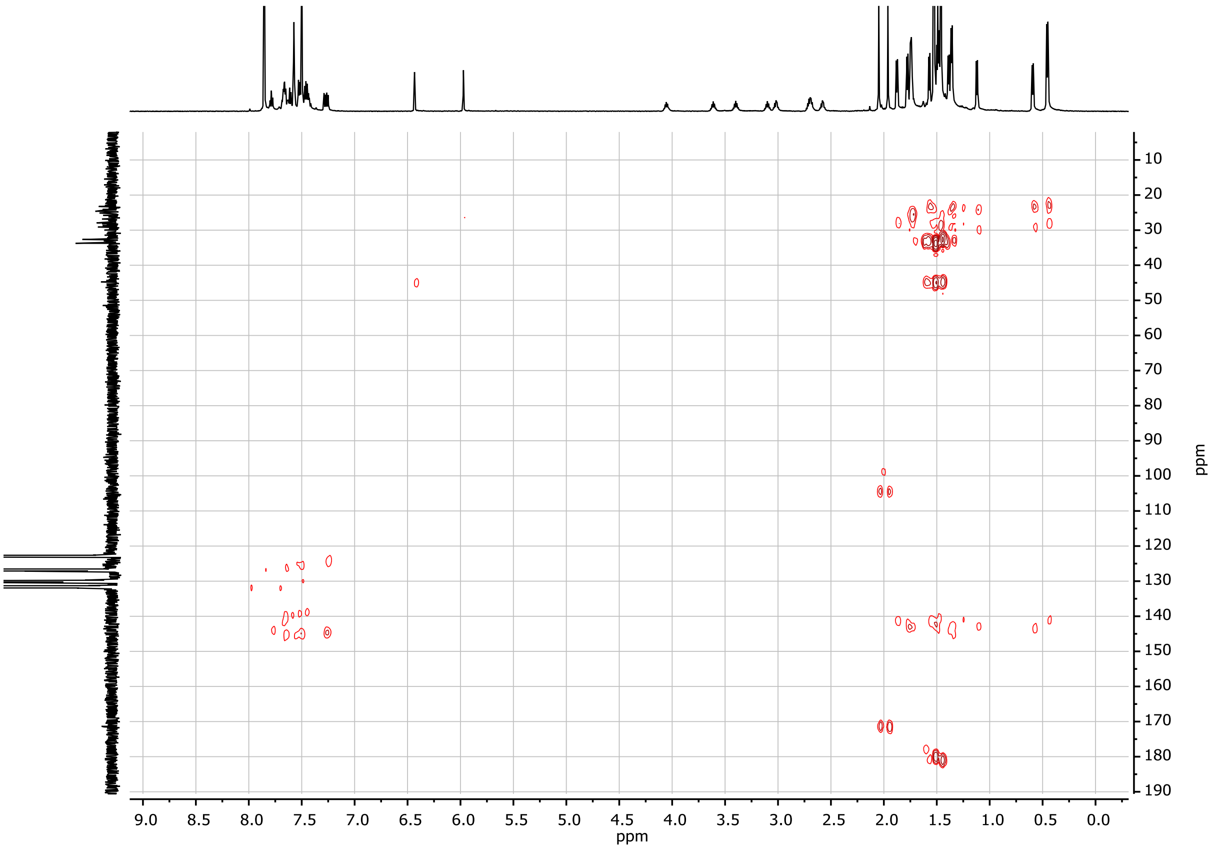

Figure S24: 2D HMBC NMR spectrum of $[(^{tBu}BDI)Zn\text{-}Ga(^{Me}BDI)^{+}][B(C_6F_5)_4^{-}]$ in C_6D_5Br.

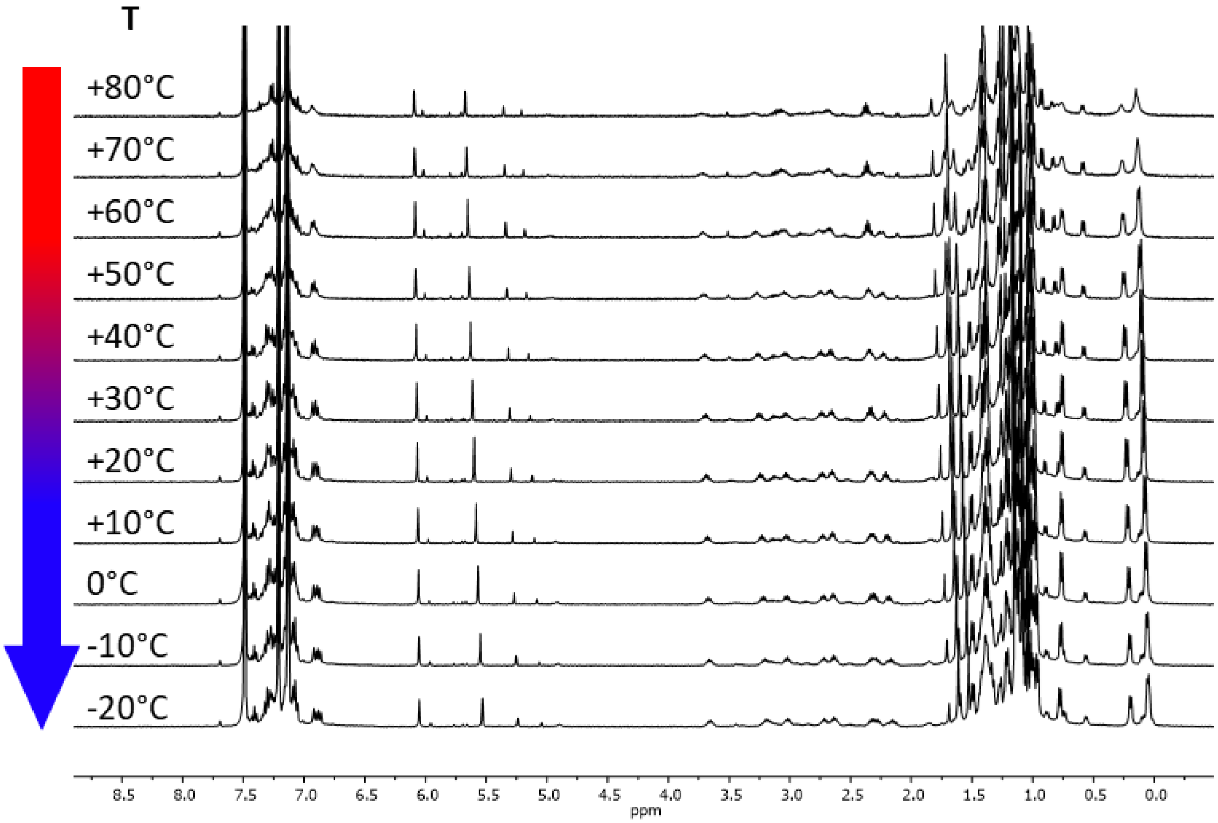

Figure S25: Temperature-dependent ^{1}H NMR (400 MHz) spectrum of $[(^{tBu}BDI)Zn\text{-}Ga(^{Me}BDI)^{+}][B(C_6F_5)_4^{-}]$ in C_6D_5Br.

3. Reactivity studies

Reaction of [(^{tBu}BDI)Zn^{+}·(C_6H_6)][B(C_6F_5)$_4^{-}$] and (^{Me}BDI)Al with fluorobenzene

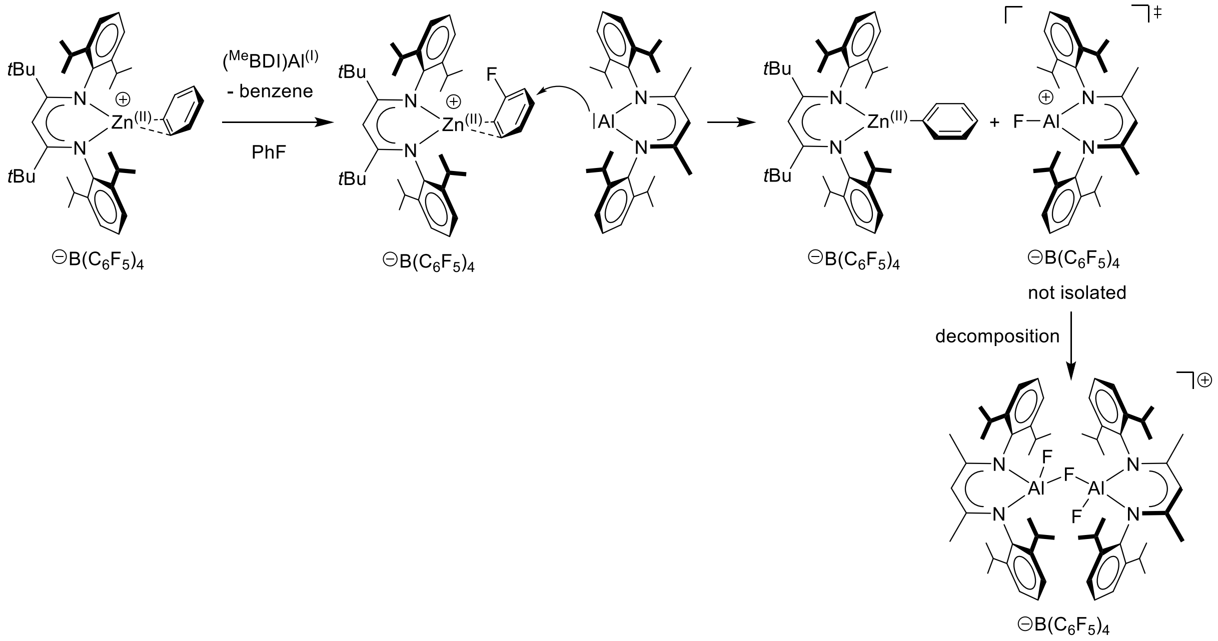

Figure S26: PhF activation with [(^{tBu}BDI)Zn(C_6H_6)$^{+}$][B(C_6F_5)$_4^{-}$] and (^{Me}BDI)Al.

(^{tBu}BDI)ZnPh: The complex was obtained as product from the reaction of [(^{tBu}BDI)Zn^{+}·benzene][B(C_6F_5)$_4^{-}$] and (^{Me}BDI)Al$^{(I)}$ in fluorobenzene. [(^{tBu}BDI)Zn·benzene]$^{+}$[B(C_6F_5)$_4$]$^{-}$ (30.2 mg, 22.8 µmol, 1 eq.) was dissolved in fluorobenzene (0.4 mL) and (^{Me}BDI)Al$^{(I)}$ (10.1 mg, 22.8 µmol, 1 eq.) was added. The pale yellow reaction mixture was stirred for 5 minutes at room temperature and addition of hexane (0.5 mL) resulted in precipitation of a white solid. After filtration, all volatiles were removed from the filtrate to give a pale white powder. (^{tBu}BDI)ZnPh was extracted with hexane and drying *in vacuo* gave the product as an off-white solid in 75% yield (11 mg). Crystals suitable for X–ray diffraction were grown from a concentrated hexane solution. In one case crystals of (^{Me}BDI)Al(F)-(µ-F)-(F)Al(^{Me}BDI)$^{+}$][B(C_6F_5)$_4^{-}$] crystallized from the mother liquor.

Alternative synthesis (^{tBu}BDI)ZnPh: To a mixture of Ph_2Zn (218 mg, 0.994 mmol, 1 eq.) and ^{tBu}BDIH (500 mg, 0.994 mmol, 1 eq.) was toluene (10 mL) added. The light-yellow solution was heating to 60 °C overnight, the solvent was removed and the solid was dried at 50 °C under vacuum. The product was isolated as off-white powder in 88% yield (553 mg).

^{1}H NMR (C_6D_6, 600 MHz, 298 K): δ 7.22 (t, 2H, aryl–*H*), 7.10 (d, 4H, aryl–*H*), 6.96 (m, 3H, Zn-*Ph*), 5.97 (m, 2H, Zn-*Ph*), 5.60 (s, 1H, ^{tBu}CC*H*C), 3.37 (sept, $^{3}J_{HH}$ = 6.9 Hz, 4H, C*H*(CH_3)$_2$), 1.28 (d, $^{3}J_{HH}$ = 6.9 Hz, 12H, CH(C*H$_3$*)$_2$), 1.23 (s, 18H, ^{tBu}BDI), 1.18 (d, $^{3}J_{HH}$ = 6.8 Hz, 12H, CH(C*H$_3$*)$_2$). **^{13}C NMR** (C_6D_6, 151 MHz, 298 K): δ 175.4 (s, ^{tBu}*C*CH*C*), 147.3 (s, aryl–*C*), 141.4 (s, aryl–*C*), 139.8 (s, aryl–*C*), 127.0 (s, aryl–*C*), 126.6 (s, aryl–*C*), 125.7 (s, aryl–*C*), 124.0 (s, aryl–*C*), 94.0 (s, ^{tBu}C*C*HC), 42.8 (s, ^{tBu}BDI), 31.9 (s, ^{tBu}BDI), 27.4 (s,

CH(CH_3)$_2$), 24.1 (s, CH(CH_3)$_2$), 22.0 (s, CH(CH_3)$_2$) ppm. **Elemental analysis** Found: C 77.31; H, 8.26; N, 4.26. Calc. for $C_{41}H_{58}N_2Zn$ (M = 644.31 g/mol): C, 76.43; H, 9.07; N, 4.35%. Even though these results are outside the range viewed as established analytically pure, they are provided to illustrate the best values obtained to date.

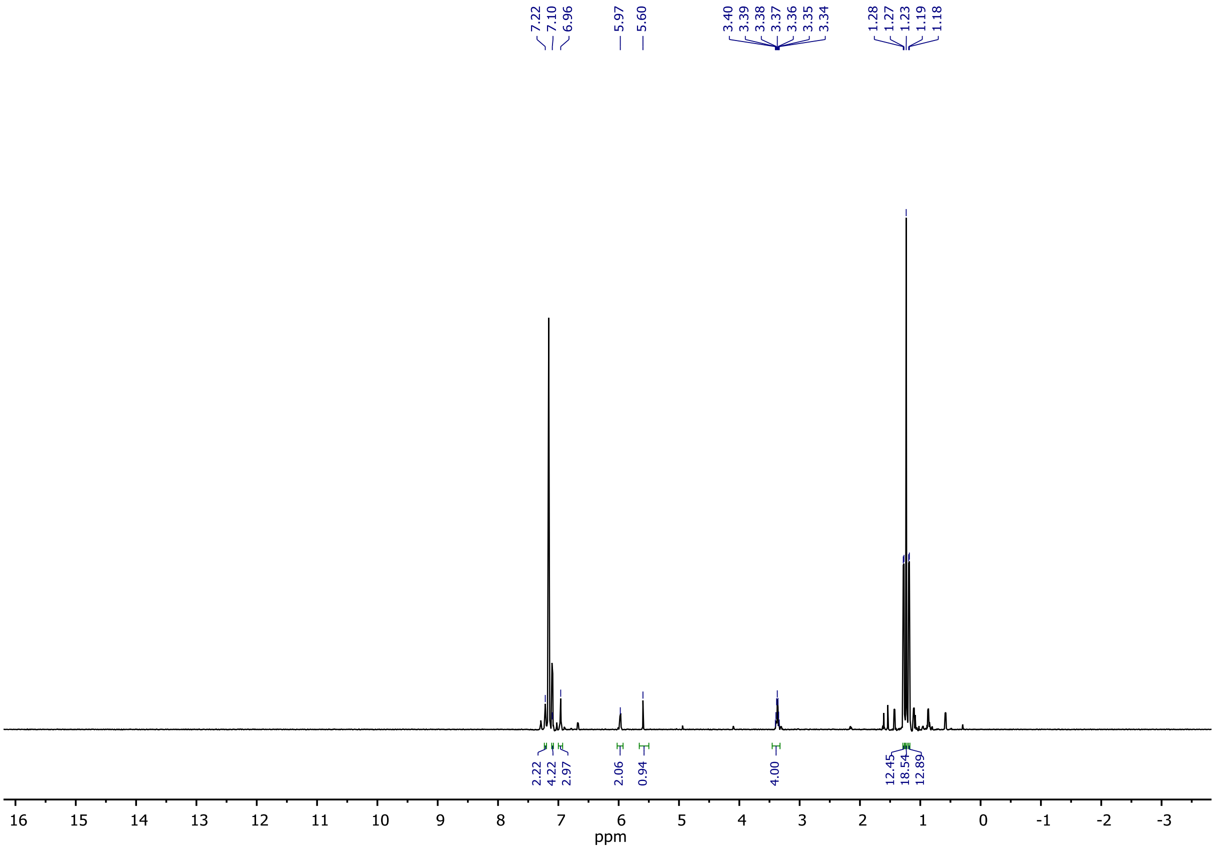

Figure S27: ^{1}H NMR (600 MHz, 298 K) spectrum of (^{tBu}BDI)ZnPh in C_6D_6.

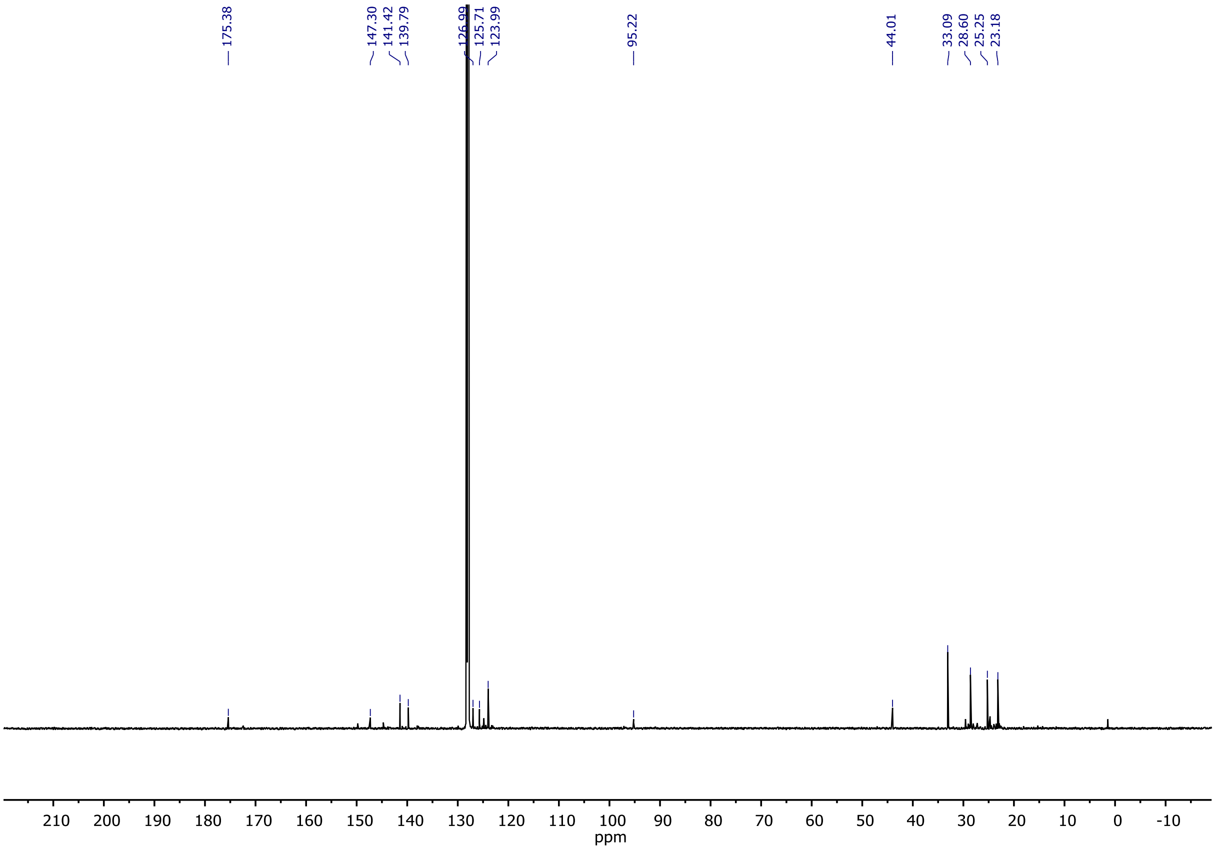

Figure S28: ^{13}C NMR (151 MHz, 298 K) spectrum of (^{tBu}BDI)ZnPh in C_6D_6.

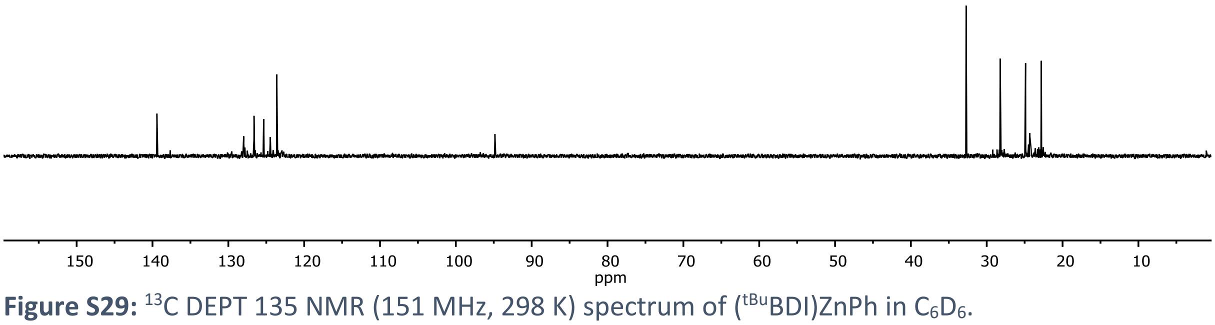

Figure S29: ^{13}C DEPT 135 NMR (151 MHz, 298 K) spectrum of (^{tBu}BDI)ZnPh in C_6D_6.

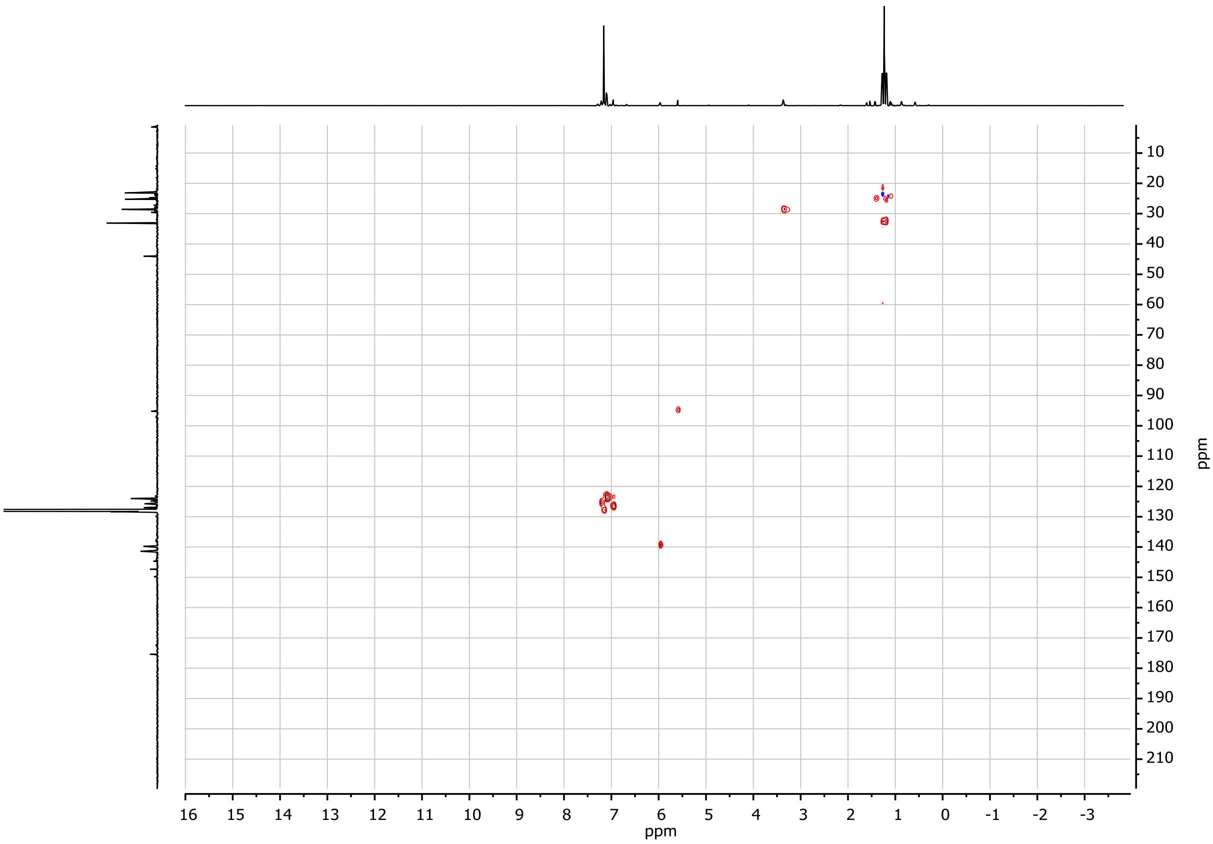

Figure S30: 2D HSQC NMR spectrum of (^{tBu}BDI)ZnPh in C_6D_6.

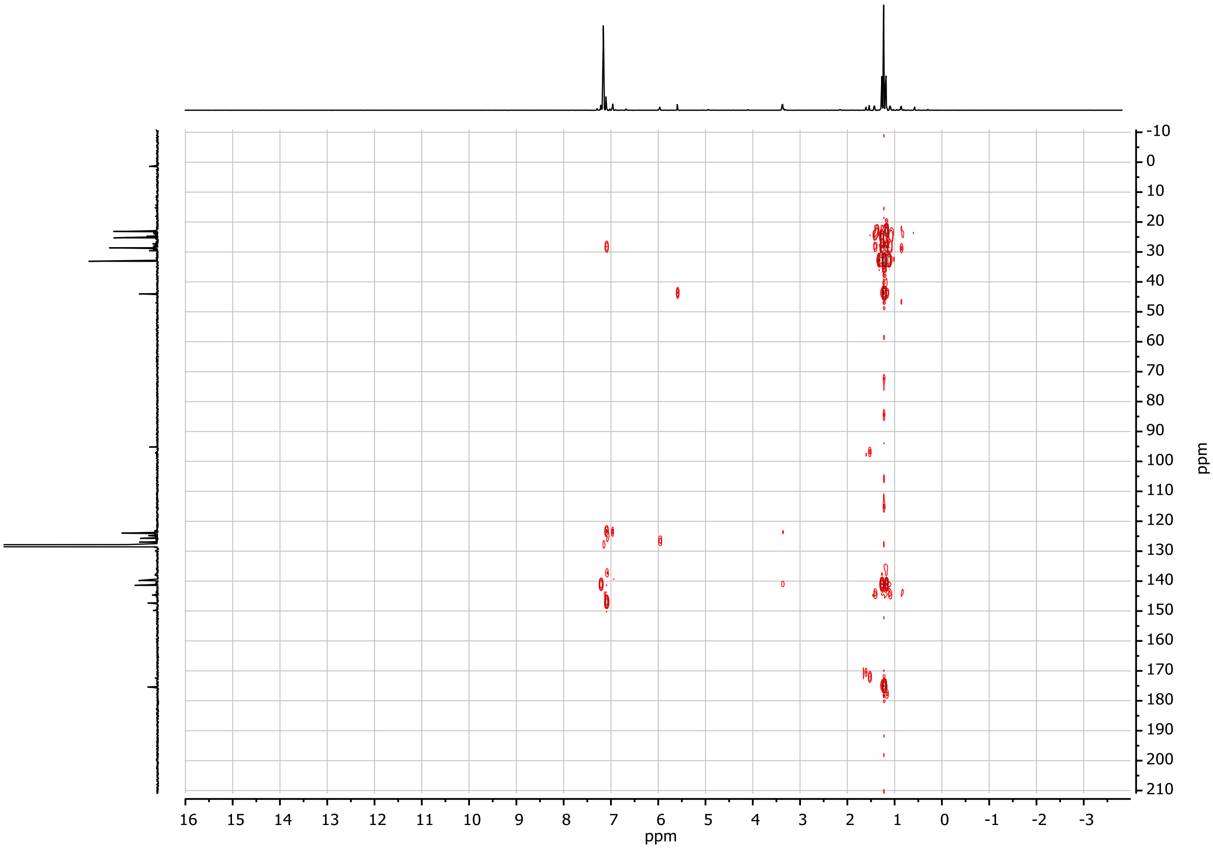

Figure S31: 2D HMBC NMR spectrum of (^{tBu}BDI)ZnPh in C_6D_6.

Onward reactivity of (^{tBu}BDI)ZnPh with I_2

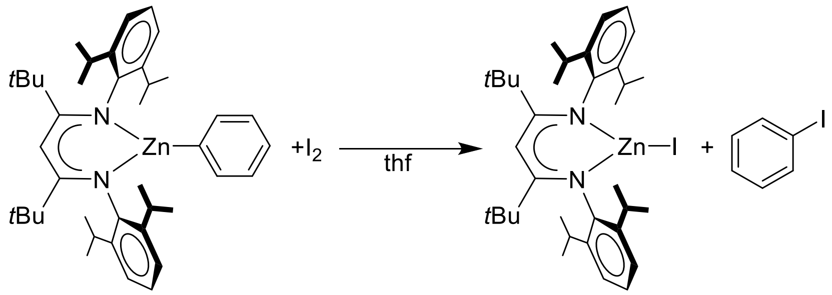

Figure S32: Reactivity of (^{tBu}BDI)ZnPh with I_2 to iodobenzene and the corresponding (^{tBu}BDI)ZnI complex.

(^{tBu}BDI)ZnI: The complex was obtained as product from the reaction of (^{tBu}BDI)ZnPh and I_2 in C_6D_6. (^{tBu}BDI)ZnPh (38.5 mg, 59.7 µmol, 1 eq.) was dissolved in C_6D_6 (550 µL), and I_2 (15.9 mg, 62.7 µmol, 1.05 eq.) was added. The mixture turned immediately light yellow and was stirred overnight at room temperature. The ^{1}H NMR shows a conversion of 83% to the corresponding (^{tBu}BDI)ZnI. The NMR signals for iodobenzene are in consistent to the literature known signals.[S9]

Alternative synthesis of (^{tBu}BDI)ZnI: ZnI_2 (118 mg, 0.370 mmol, 1 eq.) and (^{tBu}BDI)K (200 mg, 0.370 mmol, 1 eq.) were dissolved in thf (5.5 mL) and let stirred overnight at room temperature. The solvent was removed under vacuum and the resulting solid was extracted with hexane (15 mL). The extract was dried under vacuum to obtain an off-white powder in a yield of 79% (172 mg).

^{1}H NMR (C_6D_6, 600 MHz, 298 K): δ 7.10 (t, 2H, aryl-*H*), 7.04 (d, 4H, aryl-*H*), 5.59 (s, 1H, ^{tBu}CC*H*C), 3.20 (sept, $^3J_{HH}$ = 6.9 Hz, 4H, C*H*(CH_3)$_2$), 1.45 (d, $^3J_{HH}$ = 6.8 Hz, 12H, CH(C*H$_3$*)$_2$), 1.25 (d, $^3J_{HH}$ = 6.9 Hz, 12H, CH(C*H$_3$*)$_2$), 1.16 (s, 6H, ^{Me}BDI) ppm. **^{13}C NMR** (C_6D_6, 151 MHz, 298 K): δ 177.6 (s, ^{tBu}*C*CH*C*), 144.9 (s, aryl−*C*), 141.2 (s, aryl−*C*), 126.4 (s, aryl−*C*), 123.8 (s, aryl−*C*), 96.1 (s, ^{tBu}C*C*HC), 44.1 (s, ^{tBu}BDI), 33.0 (s, ^{tBu}BDI), 28.7 (s, *C*H(CH_3)$_2$), 25.6 (s, CH(*C*H$_3$)$_2$), 23.4 (s, CH(*C*H$_3$)$_2$) ppm. **Elemental analysis** Found: C 61.70; H, 7.86; N, 3.85. Calc. for $C_{35}H_{56}IN_2Zn$ (M = 602.31 g/mol): C, 60.56; H, 7.86; N, 3.85%. Even though these results are outside the range viewed as established analytically pure, they are provided to illustrate the best values obtained to date. **GC/MS (EI, 70eV):** R_T 5.40 min: m/z = 203.99 [M+] (Iodobenzene).

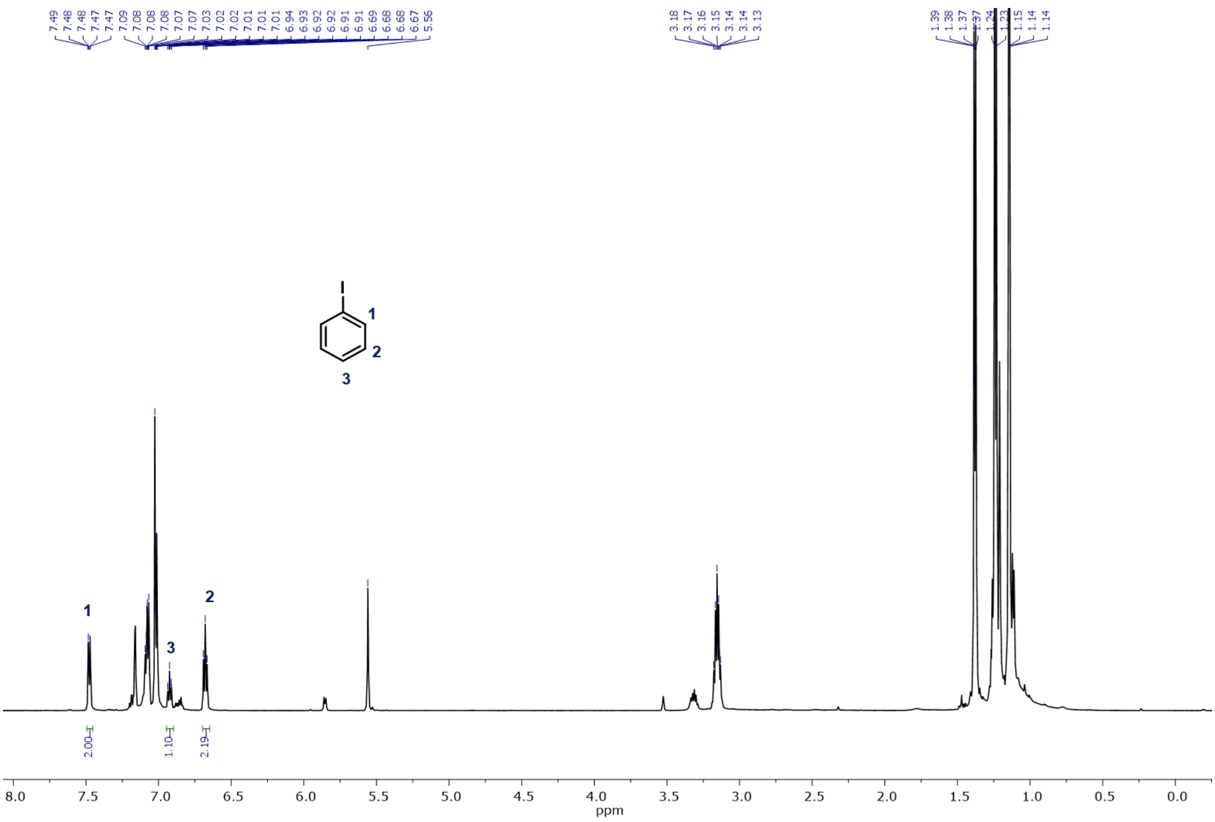

Figure S33: ^{1}H NMR (600 MHz, 298 K) spectrum of reaction mixture of (^{tBu}BDI)ZnPh and I_2 in C_6D_6, the integrated signals are of iodobenzene. See Figure below for signals of (^{tBu}BDI)ZnI.

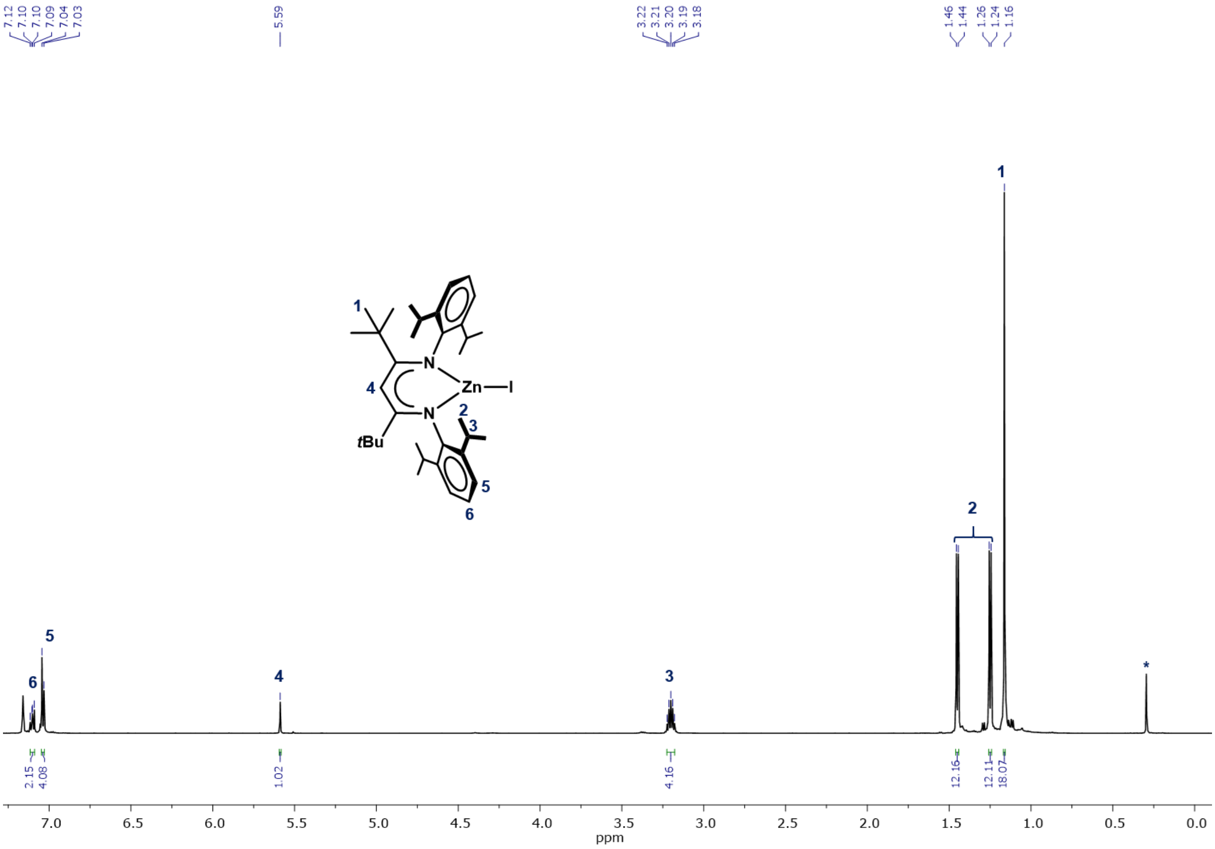

Figure S34: ^{1}H NMR (600 MHz, 298 K) spectrum of (^{tBu}BDI)ZnI in C_6D_6, (*) indicates residue of grease.

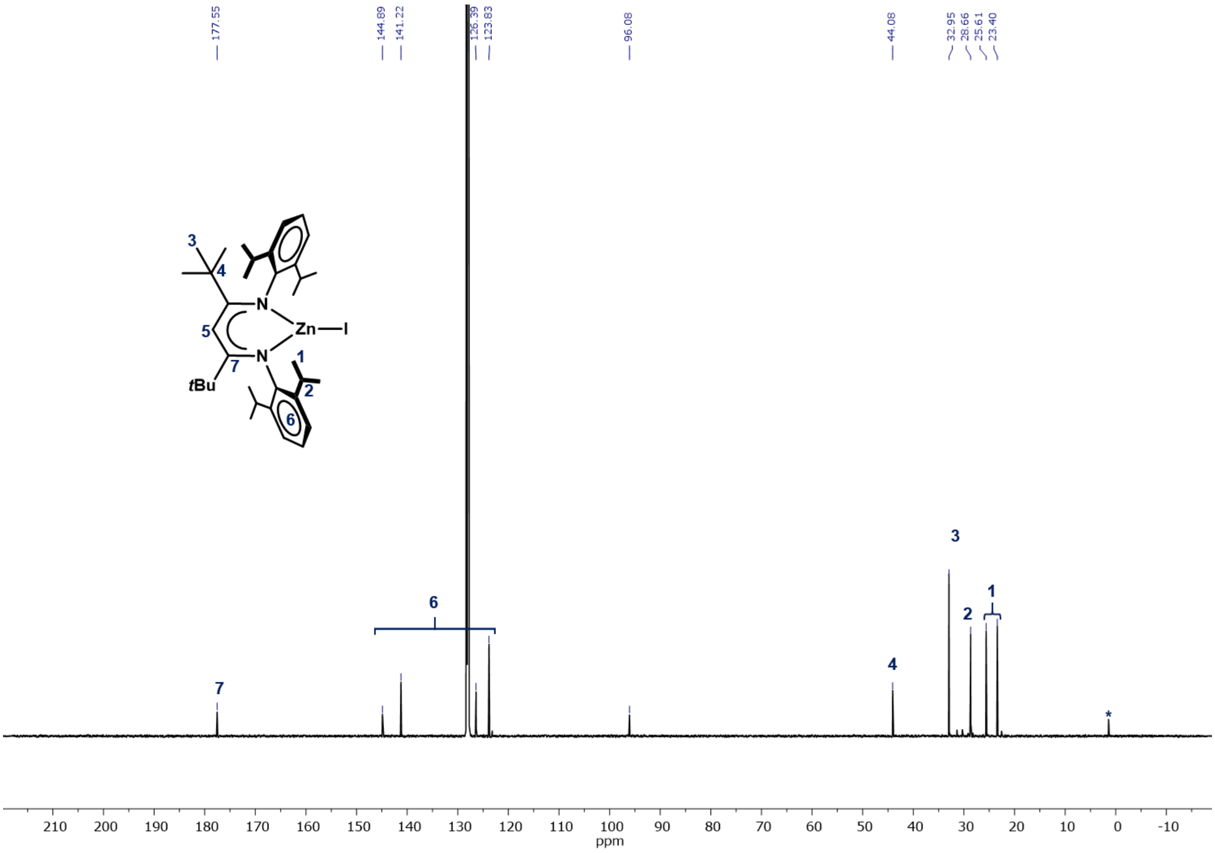

Figure S35: ^{13}C NMR (151 MHz, 298 K) spectrum of (^{tBu}BDI)ZnI in C_6D_6, (*) indicates residue of grease.

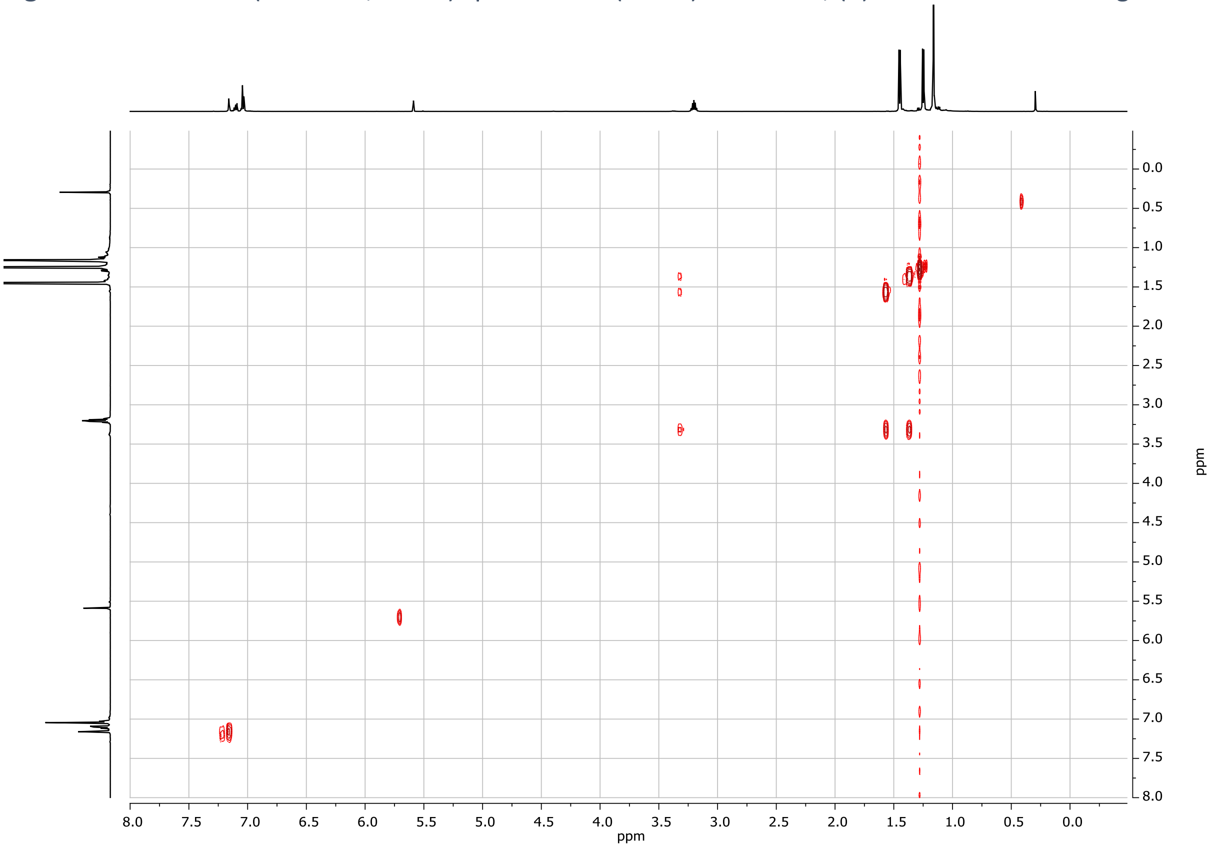

Figure S36: 2D COSY NMR spectrum of (^{tBu}BDI)ZnI in C_6D_6.

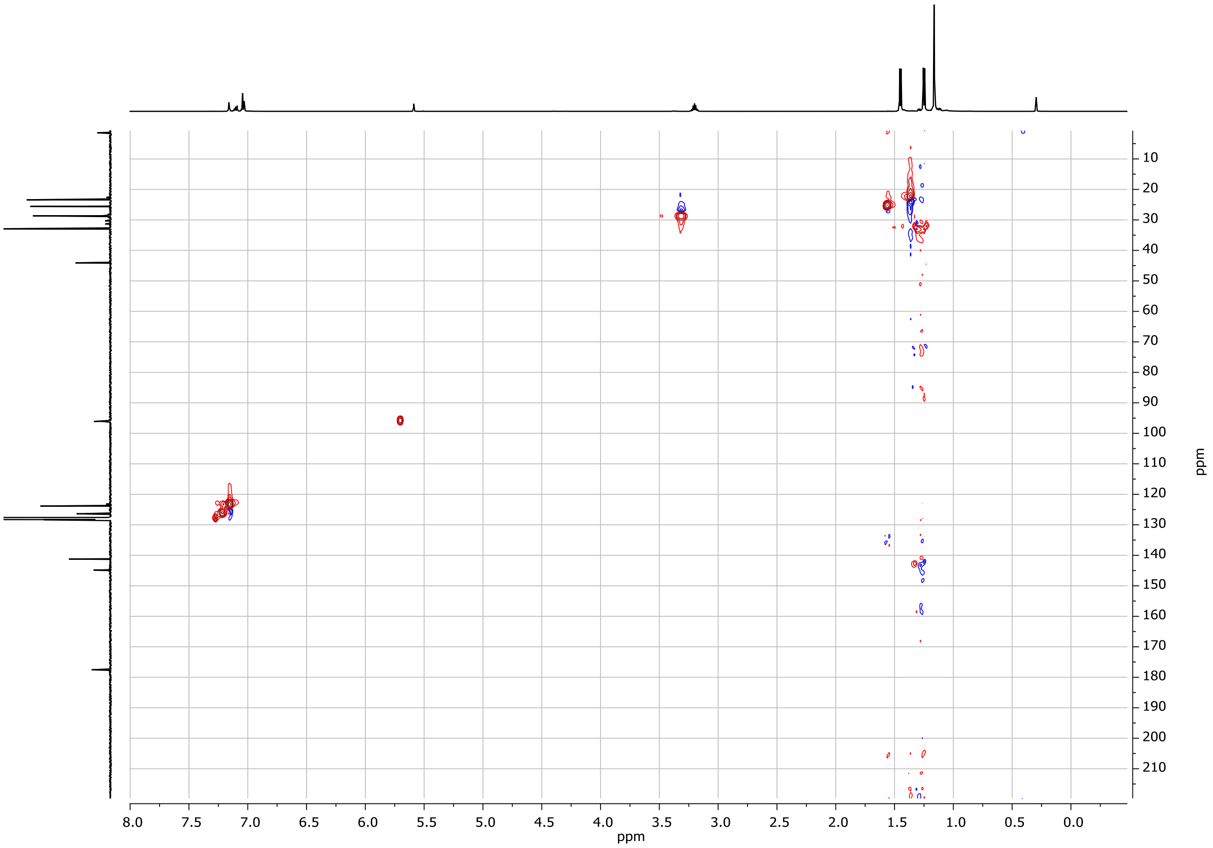

Figure S37: 2D HSQC NMR spectrum of (^{tBu}BDI)ZnI in C_6D_6.

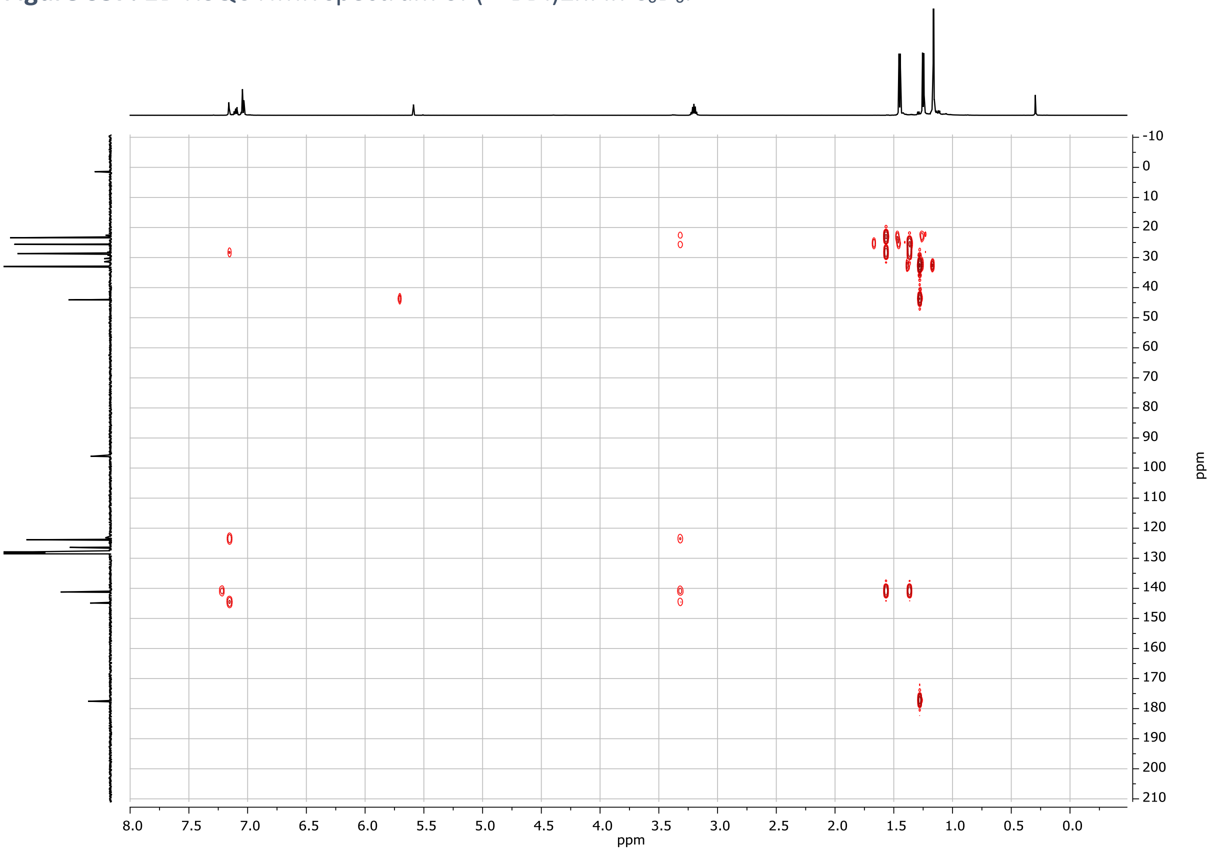

Figure S38: 2D HMBC NMR spectrum of (^{tBu}BDI)ZnI in C_6D_6.

Reaction of [(^{tBu}BDI)Zn$^+$·(C$_6$H$_6$)][B(C$_6$F$_5$)$_4^-$] and (^{Me}BDI)Al with *p*-fluorotoluene

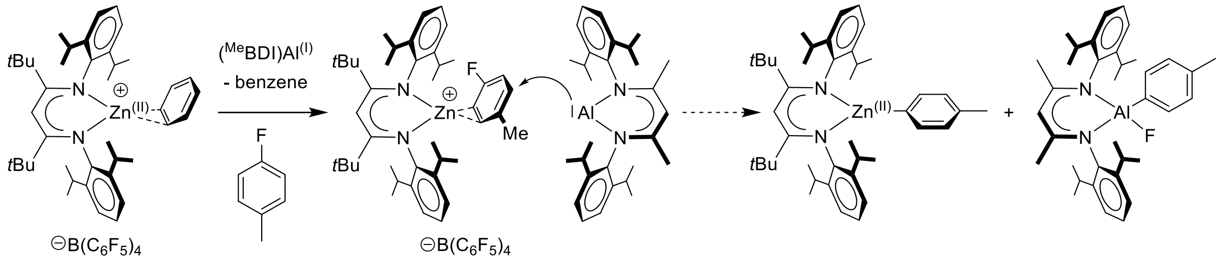

Figure S39: *p*-Fluorotoluene activation with [(^{tBu}BDI)Zn$^+$·benzene][B(C$_6$F$_5$)$_4^-$] and (^{Me}BDI)Al$^{(I)}$.

(^{tBu}BDI)Zn(*p*-tolyl): The complex was obtained as product from the reaction of [(^{tBu}BDI)Zn$^+$·benzene][B(C$_6$F$_5$)$_4^-$] and (^{Me}BDI)Al$^{(I)}$ in *p*-fluorotoluene. [(^{tBu}BDI)Zn$^+$·benzene][B(C$_6$F$_5$)$_4^-$] (32.5 mg, 24.5 µmol, 1 eq.) was dissolved in *p*-fluorotoluene (0.5 mL) and (^{Me}BDI)Al$^{(I)}$ (10.9 mg, 24.5 µmol, 1 eq.) was added. The orange reaction mixture was stirred for 5 min at room temperature and the solvent was removed. The orange foam was stripped with pentane (1 mL) and extracted with hexane (2 x 2 mL). A mixture of two products was obtained by removing all volatiles from the extract. Suitable crystals of (^{tBu}BDI)Zn(*p*-tolyl) for X-ray diffraction were grown from a concentrated pentane solution. These crystals are contaminated with a second species which could not be separated by recrystallization. Both compounds have been characterized by advanced NMR methods.

The ^{1}H NMR spectrum shows two different signals for BDI CH backbone groups. The one at 5.05 ppm belongs to ^{Me}BDI and the one at 5.60 ppm is typical for ^{tBu}BDI. Pulse-gradient DOSY NMR analysis verified the existence of two different complexes in a 1/0.75 ratio, one circa 50-100 Dalton lighter than the other. The major species is the (^{tBu}BDI)Zn(*p*-tolyl) complex. The other species could be identified as (^{Me}BDI)Al(*p*-tolyl)F by two-dimensional NMR studies. Its ^{1}H, ^{13}C and ^{19}F NMR data compare very well to that of the closely related literature-known complex (^{Me}BDI)Al(Ph)F complex.[S8]

(^{tBu}BDI)Zn(*p*-tolyl):

^{1}H NMR (C$_6$D$_6$, 600 MHz, 298 K): δ 7.24 (t, 2H, DIPP-aryl-*H*), 7.12 (d, 4H, DIPP-aryl-*H*), 6.78 (d, 2H, *C$_6$H$_4$*-CH$_3$), 5.87 (d, 2H, *C$_6$H$_4$*-CH$_3$), 5.60 (s, 1H, ^{tBu}CC*H*C), 3.38 (sept, $^3J_{HH}$ = 6.9 Hz, 4H, C*H*(CH$_3$)$_2$), 1.99 (s, 3H, C$_6$H$_4$-C*H$_3$*), 1.28 (d, $^3J_{HH}$ = 6.9 Hz, 12H, CH(C*H$_3$*)$_2$), 1.24 (s, 18H, *^{tBu}*BDI), 1.20 (d, $^3J_{HH}$ = 6.8 Hz, 12H, CH(C*H$_3$*)$_2$) ppm. **^{13}C NMR** (C$_6$D$_6$), 151 MHz, 298 K): δ 175.3 (s, ^{tBu}*C*CH*C*), 147.4 (s, DIPP-aryl-*C*), 145.4 (s, *C$_6$H$_4$*-CH$_3$), 141.5 (s, DIPP-aryl−*C*), 139.8 (s, *C$_6$H$_4$*-CH$_3$), 135.6 (s, *C$_6$H$_4$*-CH$_3$), 127.8 (s, *C$_6$H$_4$*-CH$_3$), 125.7 (s, DIPP-aryl−*C*), 124.0 (s, DIPP-aryl−*C*), 95.2 (s, *^{tBu}*CCHC), 44.0 (s, *^{tBu}*BDI), 33.1 (s, *^{tBu}*BDI), 28.5 (s, *C*H(CH$_3$)$_2$), 25.3 (s, CH(*C*H$_3$)$_2$), 23.2 (s, CH(*C*H$_3$)$_2$), 21.5 (s, C$_6$H$_4$-*CH$_3$*) ppm.

(^{Me}BDI)Al(*p*-tolyl)F:

^{1}H NMR (C$_6$D$_6$, 600 MHz, 298 K): δ 7.20 (m, 2H, DIPP-aryl-*H*), 7.20 (m, 2H, DIPP-aryl-*H*), 7.08 (dd, $^3J_{HH}$ = 6.7, 2.2 Hz, 2H, DIPP-aryl-*H*), 6.78 (d, 2H, *C$_6$H$_4$*-CH$_3$), 6.74 (d, $^3J_{HH}$ = 7.4 Hz, 2H *C$_6$H$_4$*-CH$_3$) 5.05 (s, 1H, ^{Me}CC*H*C), 3.75 (sept, $^3J_{HH}$ = 6.7 Hz, 2H, C*H*(CH$_3$)$_2$), 3.11 (sept, $^3J_{HH}$ = 6.9 Hz, 2H, C*H*(CH$_3$)$_2$), 1.95 (s, 3H,

C_6H_4-CH_3), 1.63 (s, 6H, *Me*BDI), 1.46 (d, $^3J_{HH}$ = 6.9 Hz, 6H, CH(CH_3)$_2$) 1.21 (d, $^3J_{HH}$ = 6.7 Hz, 6H, CH(CH_3)$_2$), 1.07 (d, $^3J_{HH}$ = 6.9 Hz, 6H, CH(CH_3)$_2$), 0.89 (d, $^3J_{HH}$ = 6.8 Hz, 6H, CH(CH_3)$_2$) ppm. **^{13}C NMR** (C_6D_6, 151 MHz, 298 K): δ 170.9 (s, ^{Me}*C*CH*C*), 143.8 (s, DIPP-aryl-*C*), 140.4 (s, DIPP-aryl-*C*), 138.1 (s, *C_6H_4*-CH$_3$), 137.1 (s, *C_6H_4*-CH$_3$), 127.8 (s, *C_6H_4*-CH$_3$), 127.7 (s, DIPP-aryl-*C*), 125.2 (s, DIPP-aryl-*C*), 124.4 (s, DIPP-aryl-*C*), 97.9 (s, ^{Me}C*C*HC), 29.0 (s, *C*H(CH$_3$)$_2$), 28.5 (s, *C*H(CH$_3$)$_2$), 25.8 (s, CH(*C*H$_3$)$_2$), 24.7 (s, CH(*C*H$_3$)$_2$), 24.5 (s, CH(*C*H$_3$)$_2$), 23.8 (s, CH(*C*H$_3$)$_2$), 23.2 (s, *Me*BDI), 21.5 (s, C_6H_4-*C*H$_3$) ppm. The quaternary C attached to Al is not visible due to quadrupole line-broadening (a similar observation was made for (*Me*BDI)Al(Ph)F).[S8] **^{19}F NMR** (C_6D_6, 565 MHz, 298 K): δ -159.1 (s, 1F, Al-F) ppm.

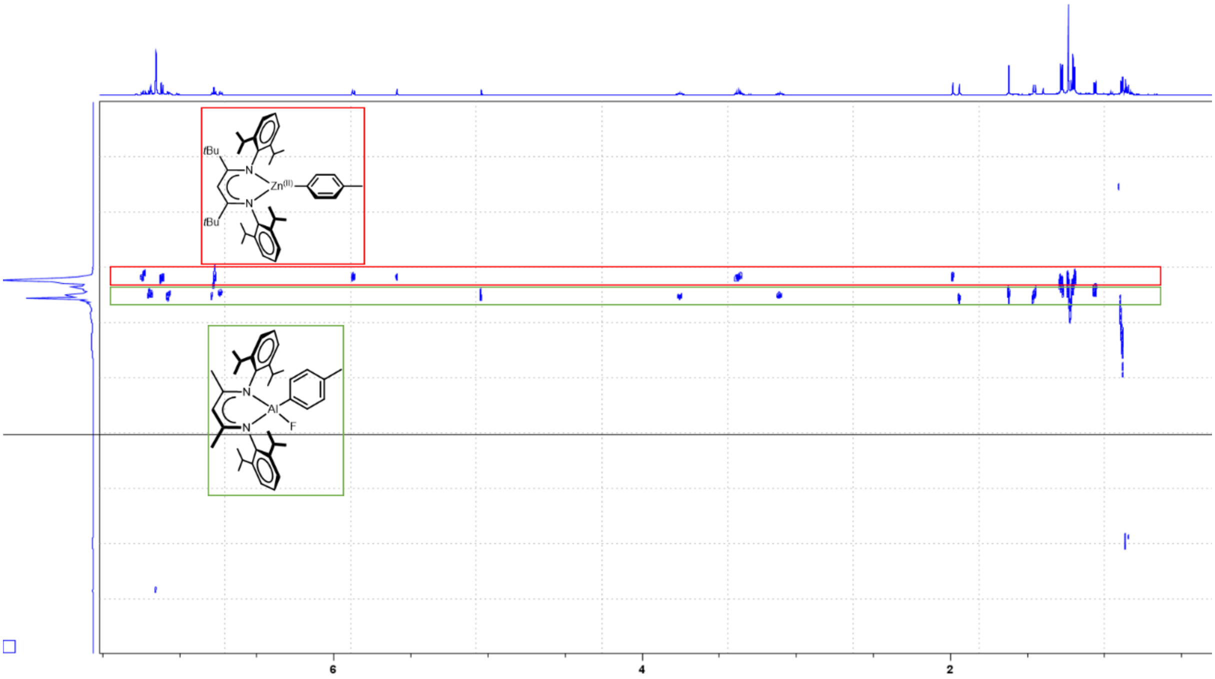

Figure S40: Diffusion NMR spectrum of a mixture of (^{tBu}BDI)Zn(*p*-tolyl) and (^{Me}BDI)Al(*p*-tolyl)F in C_6D_6.

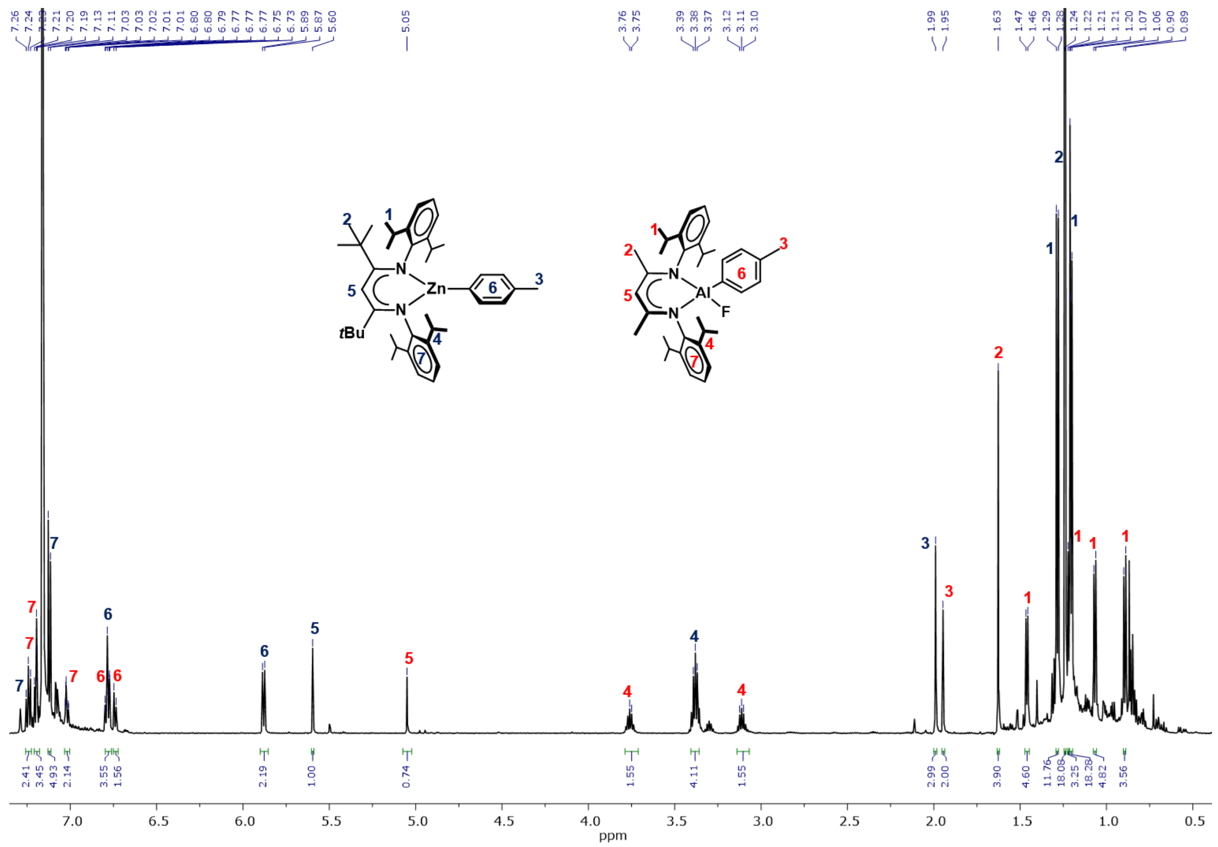

Figure S41: ^{1}H NMR (600 MHz, 298 K) spectrum of a mixture of (^{tBu}BDI)Zn(*p*-tolyl) and (^{Me}BDI)Al(*p*-tolyl)F in C_6D_6.

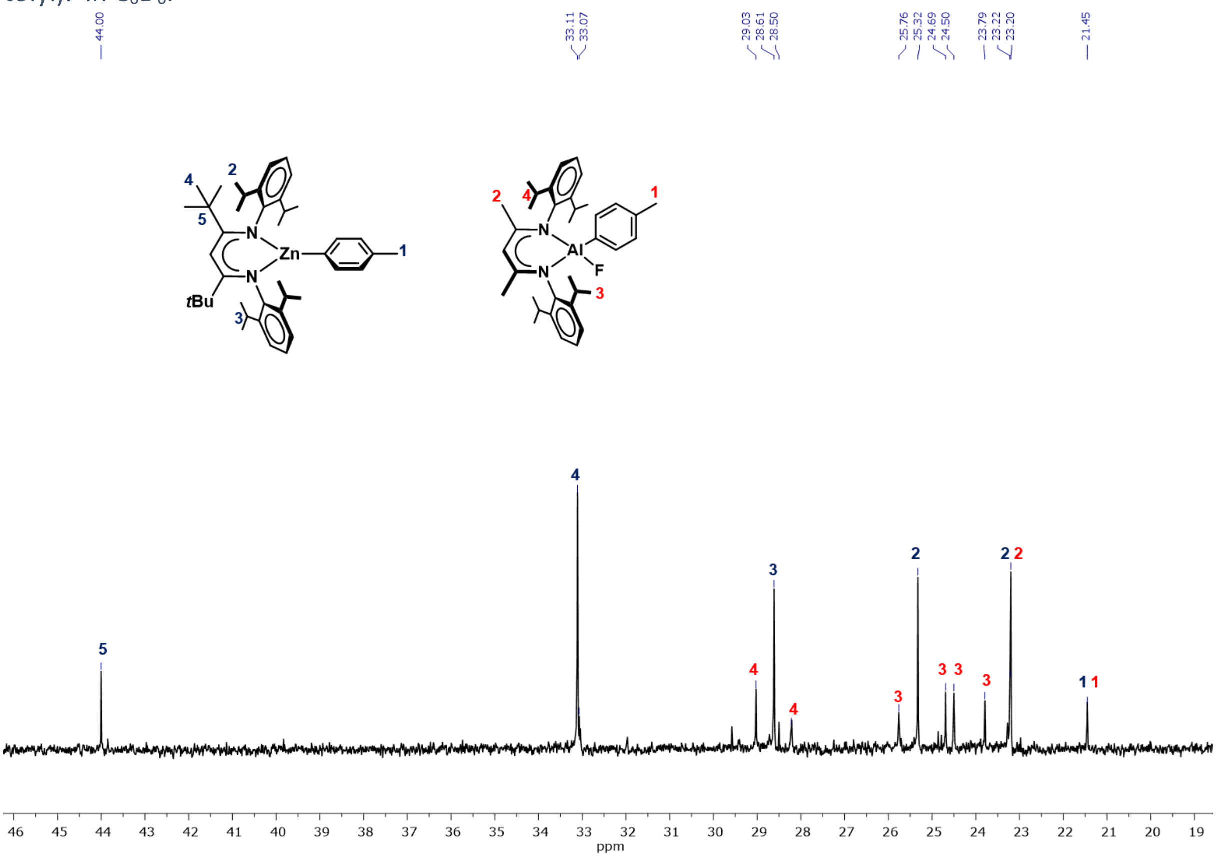

Figure S42: ^{13}C NMR (151 MHz, 298 K) spectrum of a mixture of (^{tBu}BDI)Zn(p-tolyl) and (^{Me}BDI)Al(p-tolyl)F in C_6D_6, area from 18 – 47 ppm.

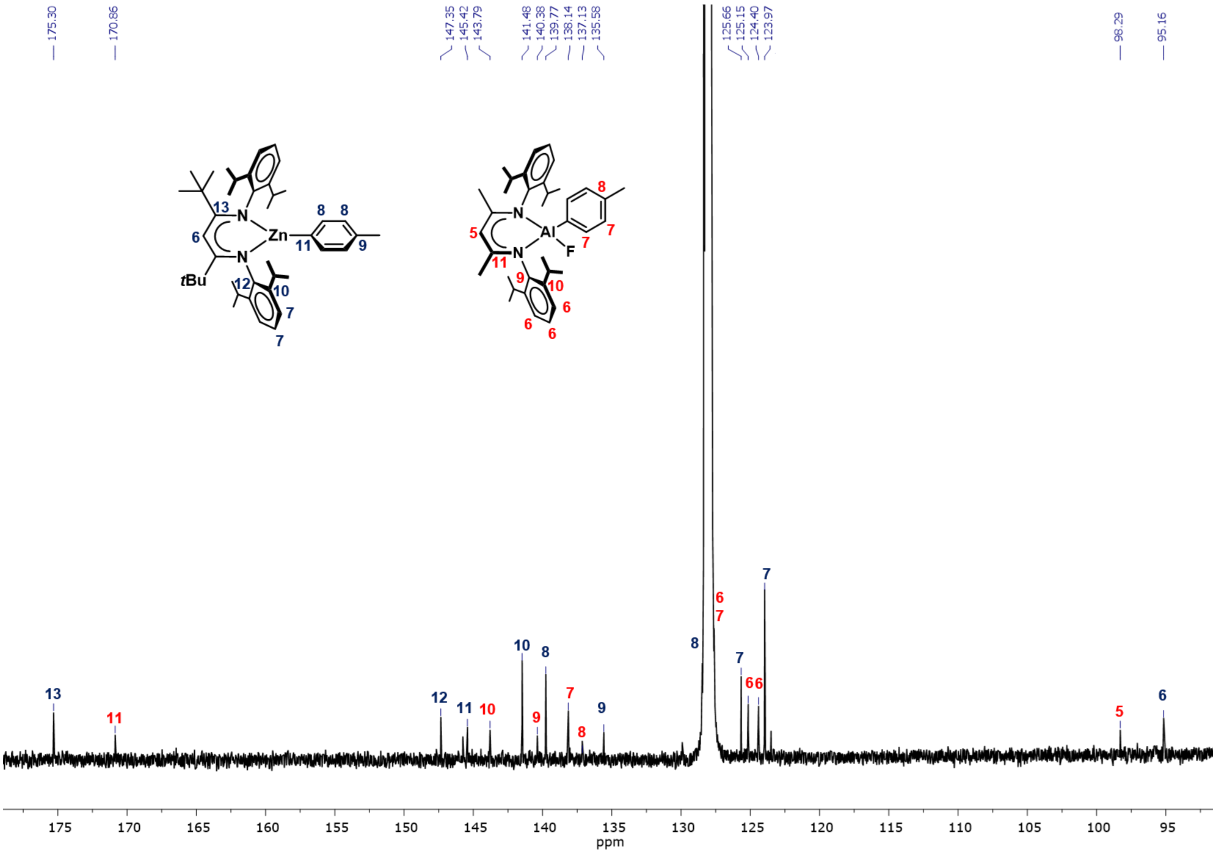

Figure S43: ^{13}C NMR (151 MHz, 298 K) spectrum of a mixture of (^{tBu}BDI)Zn(*p*-tolyl) and (^{Me}BDI)Al(*p*-tolyl)F in C_6D_6, area from 92 – 180 ppm.

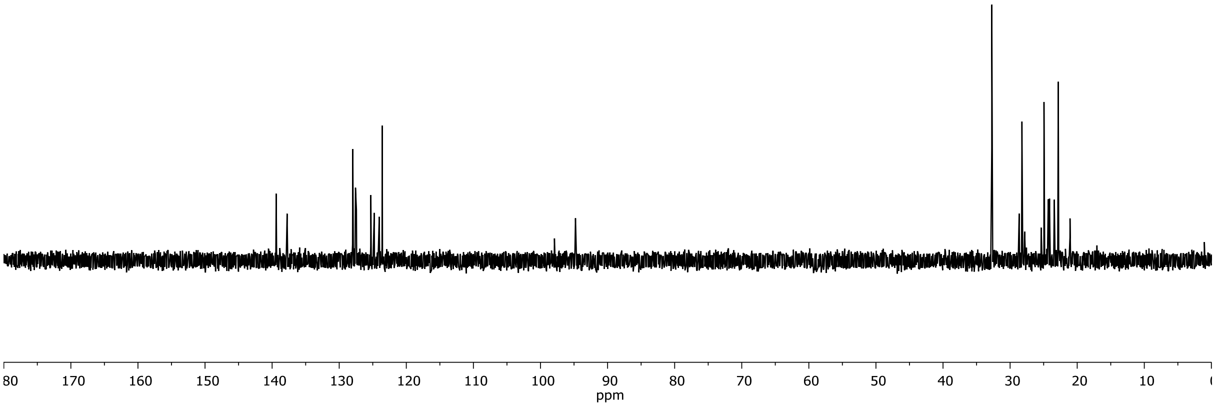

Figure S44: ^{13}C DEPT135 NMR (151 MHz, 298 K) spectrum of a mixture of (^{tBu}BDI)Zn(*p*-tolyl) and (^{Me}BDI)Al(*p*-tolyl)F in C_6D_6.

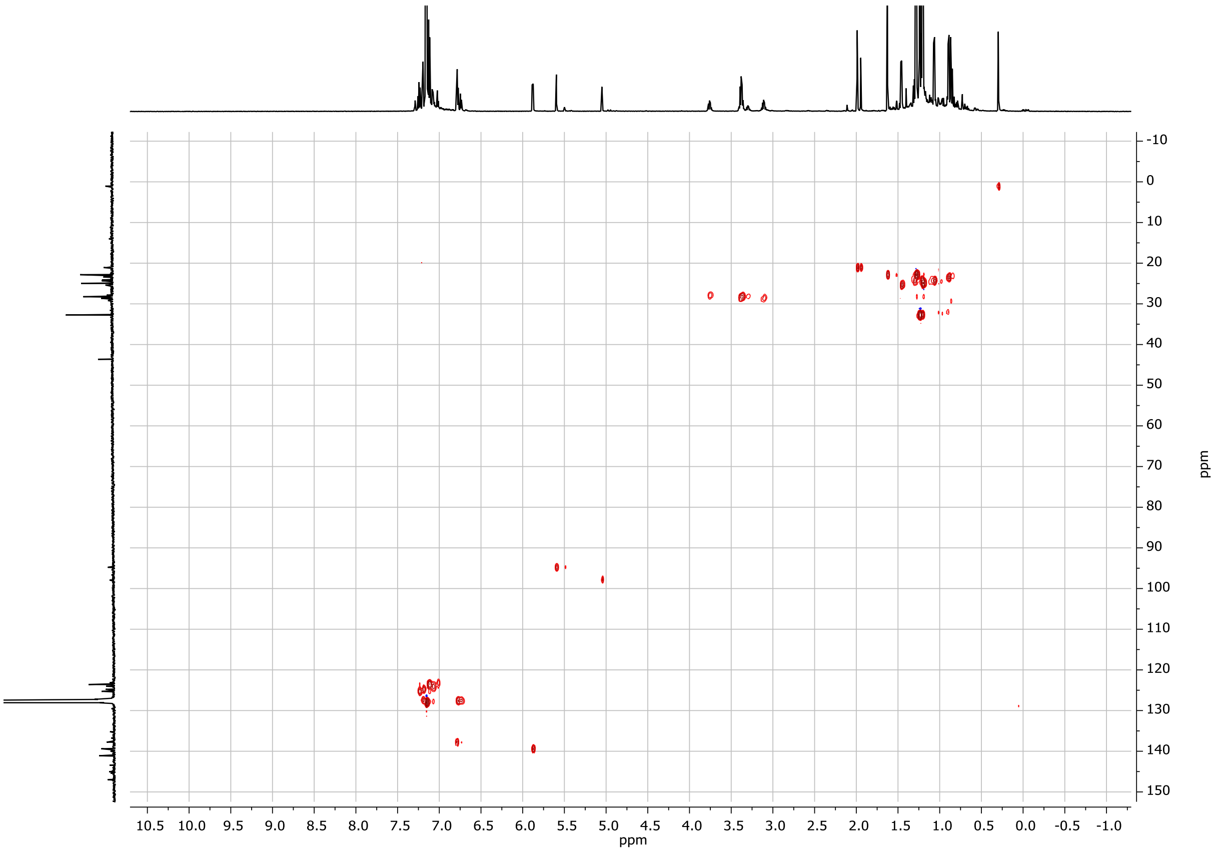

Figure S45: 2D HSCQ NMR spectrum of a mixture of (^{tBu}BDI)Zn(*p*-tolyl) and (^{Me}BDI)Al(*p*-tolyl)F in C_6D_6.

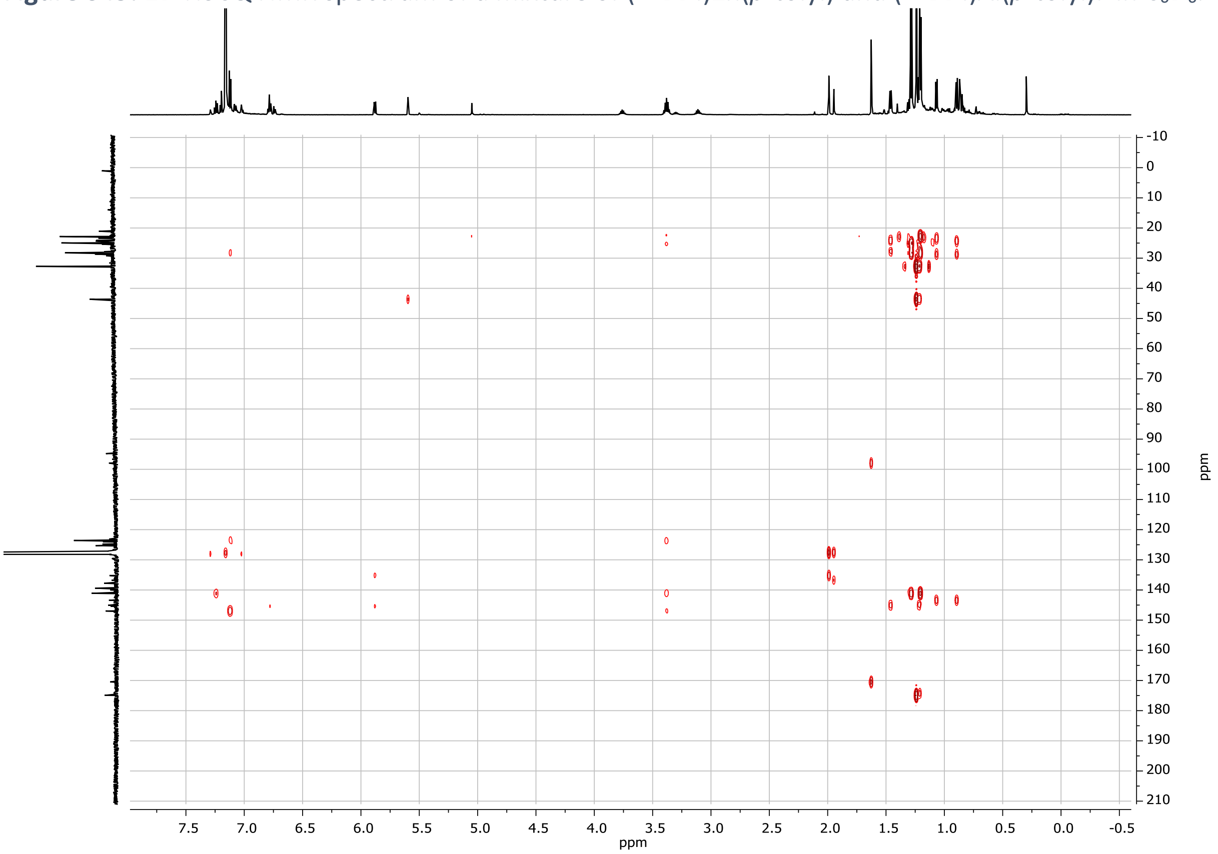

Figure S46: 2D HMBC NMR spectrum of a mixture of (^{tBu}BDI)Zn(*p*-tolyl) and (^{Me}BDI)Al(*p*-tolyl)F in C_6D_6.

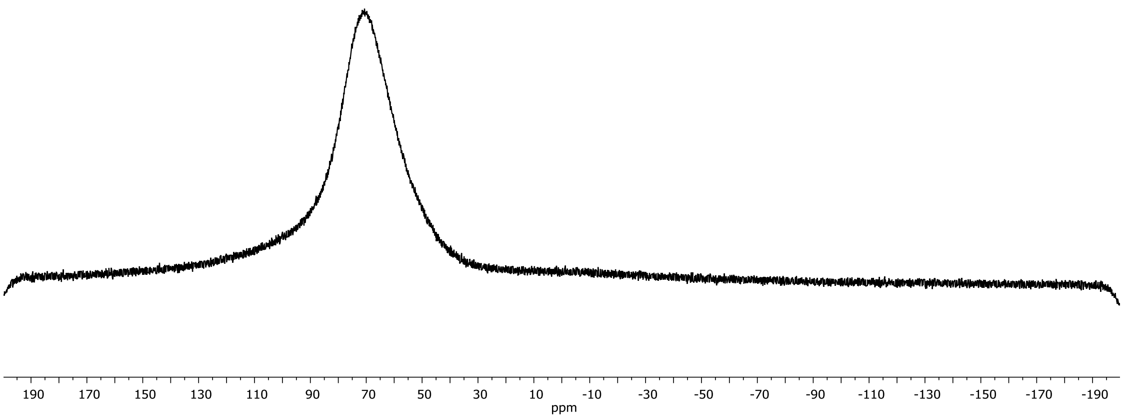

Figure S47: **^{27}**Al NMR (156 MHz, 298 K) spectrum of a mixture of (^{tBu}BDI)Zn(*p*-tolyl) and (^{Me}BDI)Al(*p*-tolyl)F in C_6D_6.

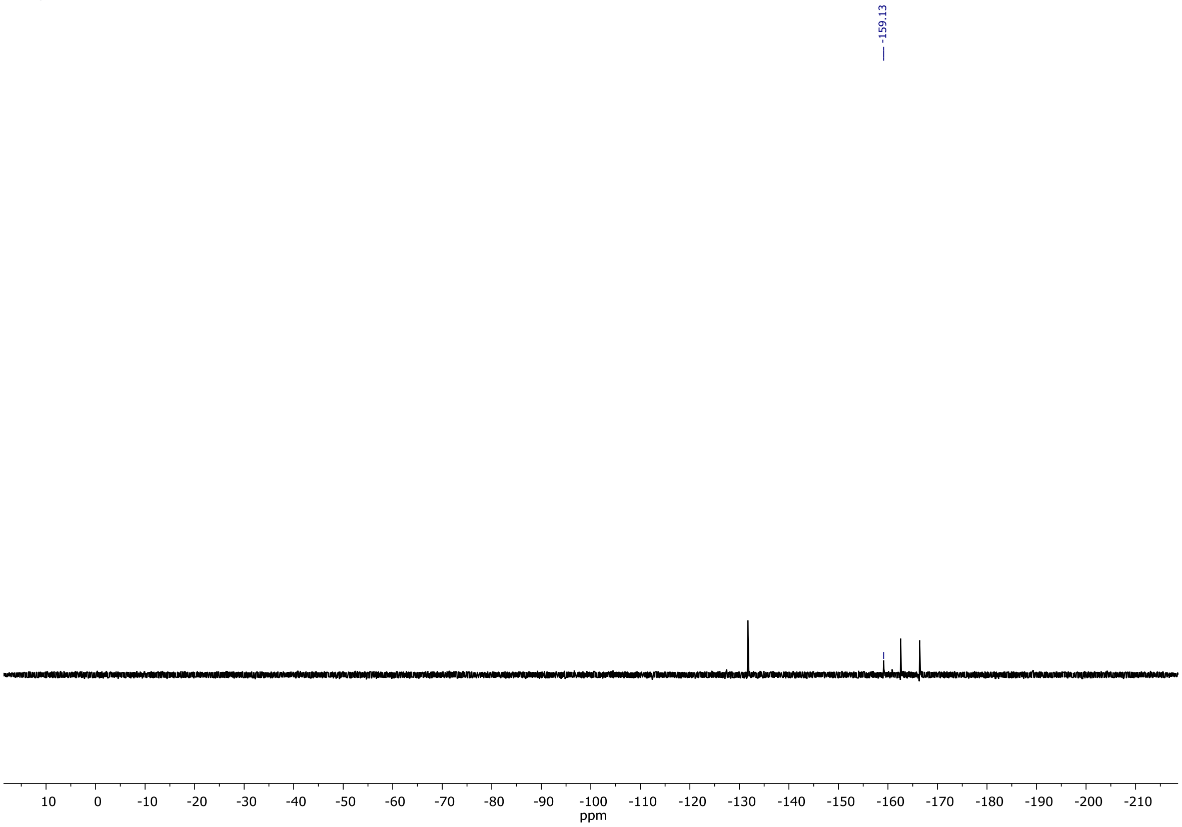

Figure S48: ^{19}F NMR (565 MHz, 298 K) spectrum mixture of (^{tBu}BDI)Zn(*p*-tolyl) and (^{Me}BDI)Al(*p*-tolyl)F in C_6D_6. Traces of $[B(C_6F_5)_4]^-$ are still observable (-166.4, -162.6, -131.7 ppm).

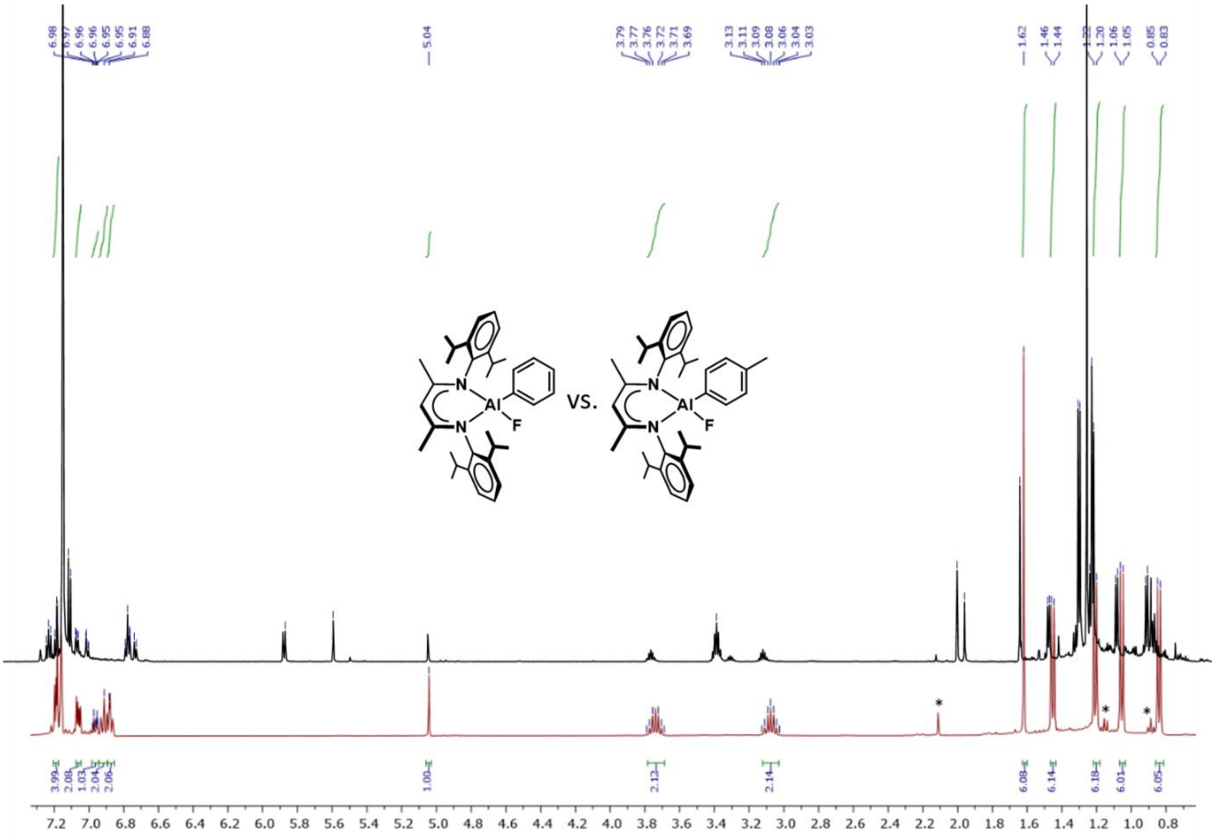

Figure S49: ^{1}H NMR spectrum comparison of (^{Me}BDI)Al(*p*-tolyl)F and (^{Me}BDI)Al(Ph)F in C_6D_6.[S8]

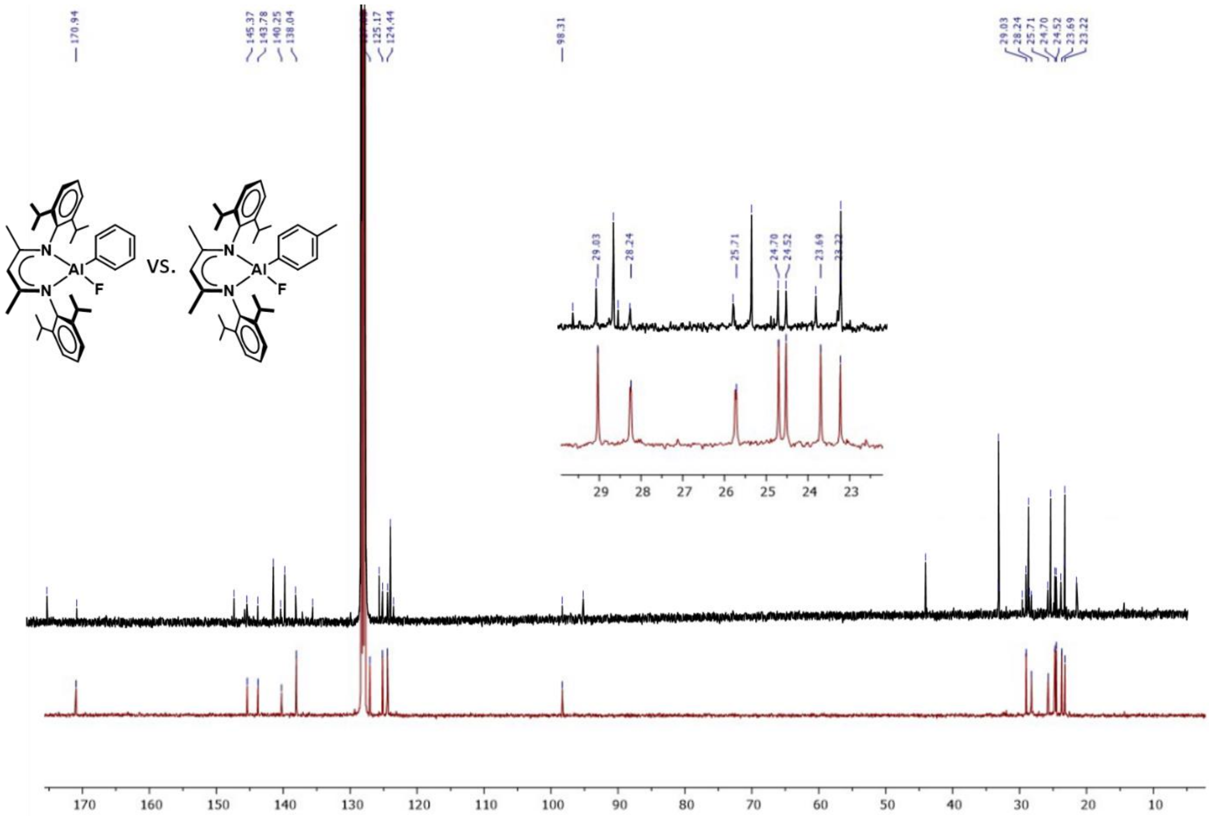

Figure S50: ^{13}C NMR spectrum comparison of (^{Me}BDI)Al(*p*-tolyl)F and (^{Me}BDI)Al(Ph)F in in C_6D_6.[S8]

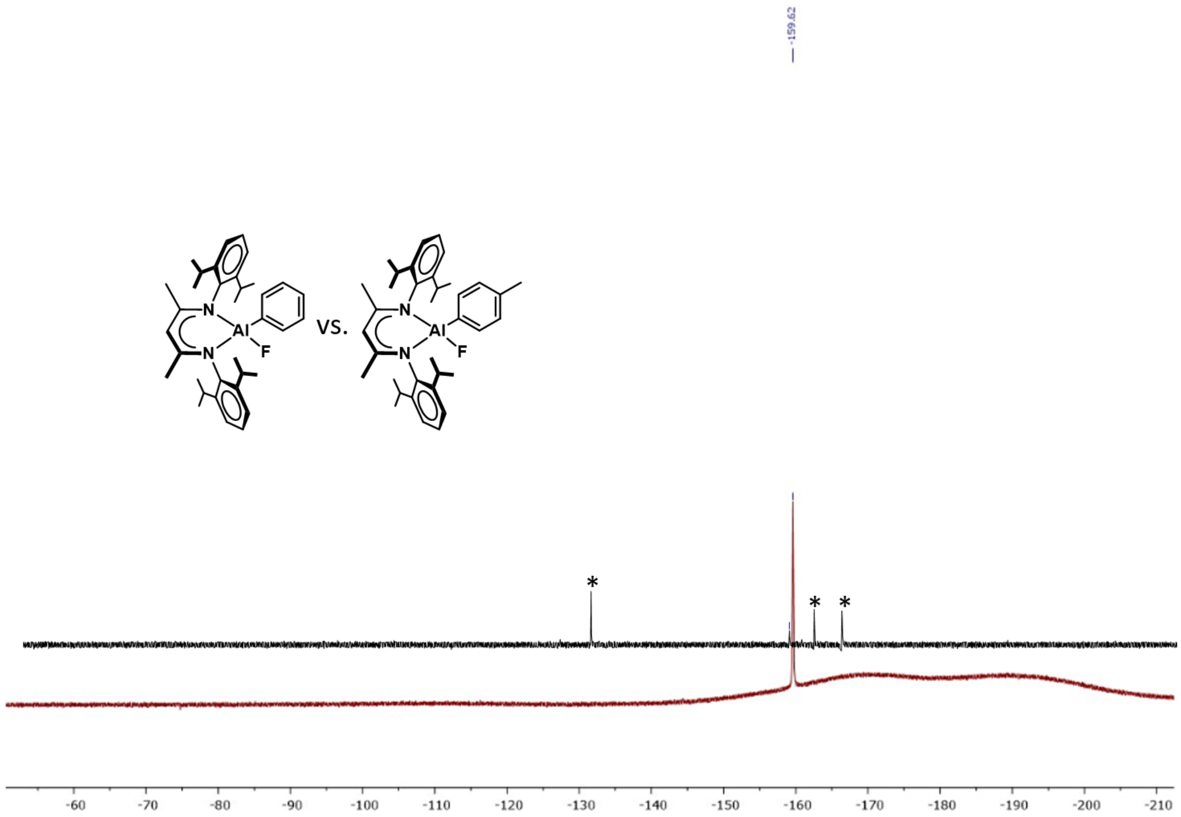

Figure S51: ^{19}F NMR spectrum comparison of (^{Me}BDI)Al(*p*-tolyl)F and (^{Me}BDI)Al(Ph)F in C_6D_6.[S8] The * indicates residual $B(C_6F_5)_4^-$.

4. Single Crystal X-Ray Diffraction

A crystal of the corresponding compound was embedded in inert perfluoropolyalkylether (viscosity 1800 cSt; ABCR GmbH) and mounted using a Hampton Research CryoLoop. The crystal was then flash cooled to 100.0(6) K in a nitrogen gas stream and kept at this temperature during the experiment. The crystal structure was measured on an Agilent SuperNova diffractometer with Atlas S2 detector using a CuKα microfocus source. The measured data was processed with the CrysAlisPro (v40.53) software package.[S10] Using Olex2,[S11] the structure was solved with the ShelXT[S12] structure solution program using Intrinsic Phasing and refined with the ShelXL[S13] refinement package using Least Squares Minimization. All non-hydrogen atoms were refined anisotropically. CCDC Nr's are shown in Table S1.

[(^{tBu}BDI)Mg-Al(^{Me}BDI)$^+$][B(C_6F_5)$_4^-$]: Most hydrogen atoms were placed in ideal positions and refined as riding atoms with relative isotropic displacement parameters. Since the hydrogen atoms at C2 and C38 in the ligand backbones deviate slightly from the idealized geometry, their positions were located from difference Fourier maps and refined. Disorder of two isopropyl group and of the co-crystallized fluorobenzene was observed. The disorder was modeled with the help of similarity restraints (SIMU, SADI) and rigid bond restraints (RIGU).[S14] The relative occupancies of the two alternative orientations of the isopropyl groups were refined to 0.835(8)/0.165(8) (*i*Pr 1) and 0.80(2)/0.20(2) (*i*Pr 2), respectively. In case of fluorobenzene, which is disordered about a 2-fold rotation axis, the occupancy was set to 0.5.

[(^{tBu}BDI)Mg-Ga(^{Me}BDI)$^+$][B(C_6F_5)$_4^-$]: Most hydrogen atoms were placed in ideal positions and refined as riding atoms with relative isotropic displacement parameters. Since the hydrogen atoms at C2 and C38 in the ligand backbones deviate slightly from the idealized geometry, their positions were located from difference Fourier maps and refined. Disorder of two isopropyl groups and of the co-crystallized fluorobenzene was observed. The disorder was modeled with the help of similarity restraints (SIMU, SADI) and rigid bond restraints (RIGU).[S14] The relative occupancies of the two alternative orientations of the isopropyl groups were refined to 0.705(11)/0.295(11) (*i*Pr 1) and 0.66(3)/0.34(3) (*i*Pr 2), respectively. In case of fluorobenzene, which is disordered about a 2-fold rotation axis, the occupancy was set to 0.5.

[(^{tBu}BDI)Zn-Ga(^{Me}BDI)$^+$][B(C_6F_5)$_4^-$]: Most hydrogen atoms were placed in ideal positions and refined as riding atoms with relative isotropic displacement parameters. Since the hydrogen atoms at C2 and C38 in the ligand backbones deviate slightly from the idealized geometry, their positions were located from difference Fourier maps and refined. Disorder of two isopropyl groups and of the co-crystallized fluorobenzene was observed. The disorder was modeled with the help of similarity restraints (SIMU, SADI). The relative occupancies of the two alternative orientations of the isopropyl groups were refined

to 0.845(6)/0.155(6) (*i*Pr 1) and 0.56(3)/0.44(3) (*i*Pr 2). In case of fluorobenzene, which is disordered about a 2-fold rotation axis, the occupancy was set to 0.5.

(^{tBu}BDI)ZnPh: All hydrogen atoms were placed in ideal positions and refined as riding atoms with relative isotropic displacement parameters. Disorder of one *tert*-butyl group was observed and modeled with the help of one rigid bond restraint (RIGU).[S14] The relative occupancies of the two alternative orientations were refined to 0.880(5) and 0.120(5).

(^{tBu}BDI)Zn(*p*-tolyl): All hydrogen atoms were placed in ideal positions and refined as riding atoms with relative isotropic displacement parameters. Disorder of one *tert*-butyl group and one isopropyl group was observed and modeled with the help of similarity restraints (SADI, SIMU). The relative occupancies of the two alternative orientations were refined to 0.672(3)/0.328(3) (*t*Bu) and 0.54(3)/0.46(3) (*i*Pr), respectively.

[(^{Me}BDI)Al(F)-(μ-F)-(F)Al(^{Me}BDI)$^{+}$][B(C_6F_5)$_4^{-}$]: A colorless crystal was embedded in inert perfluoropolyalkylether (viscosity 1800 cSt; ABCR GmbH) and mounted using a Hampton Research CryoLoop. The crystal was then flash cooled to 100.0(2) K in a nitrogen gas stream and kept at this temperature during the experiment. The crystal structure was measured on an Agilent SuperNova diffractometer with Atlas S2 detector using a CuKα microfocus source. The measured data was processed with the CrysAlisPro (v40.67a) software package.[S15] Using Olex2,[S11] the structure was solved with the ShelXT[S12] structure solution program using Intrinsic Phasing and refined with the ShelXL[S13] refinement package using Least Squares Minimization. Due to poor crystal quality and B- and C-level alerts in the checkcif tests,[S16] bond lengths and angles are not discussed and only the connectivity of [(^{Me}BDI)Al(F)-(μ-F)-(F)Al(^{Me}BDI)$^{+}$][B(C_6F_5)$_4^{-}$] is shown (Figure S33). Therefore, only the spacegroup P-1 and the cell parameters (a: 12.5289(4) Å, b: 16.8221(7) Å, c: 18.5446(8) Å, α: 98.878(4)°, β: 94.600(3)°, γ: 94.396(3)°, V = 3833.5(3) Å^3) are reported and a only ball-and-stick representation of the molecule is shown.

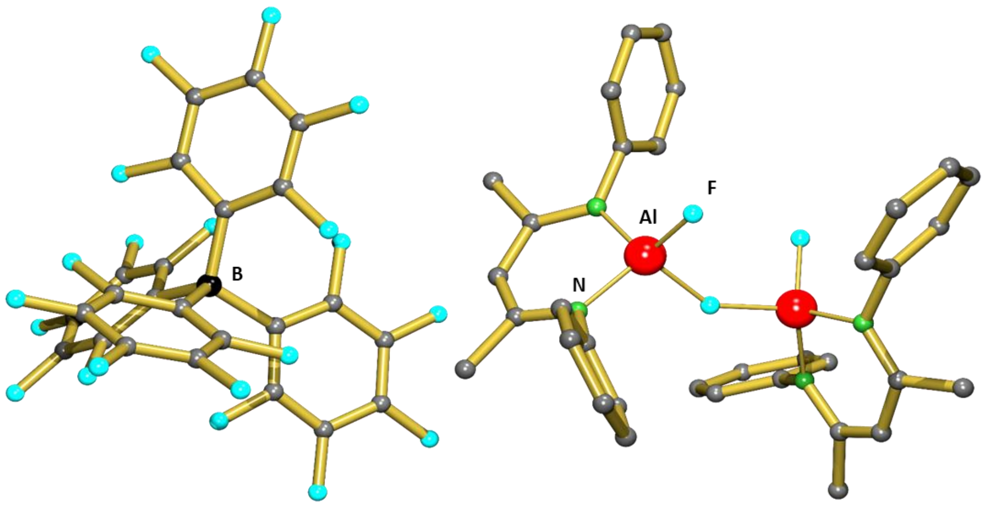

Figure S52 Ball-and-stick representation of [(^{Me}BDI)Al(F)-(μ-F)-(F)Al(^{Me}BDI)$^+$][B(C_6F_5)$_4$$^-$]. *i*Pr groups and hydrogen atoms were omitted for clarity.

Table S1. Crystal data.

Identification code	$[(^{tBu}BDI)Mg\text{-}Al(^{Me}BDI)^+][B(C_6F_5)_4^-]\cdot 0.5\ PhF$	$[(^{tBu}BDI)Mg\text{-}Ga(^{Me}BDI)^+][B(C_6F_5)_4^-]\cdot 0.5\ PhF$
Empirical formula	$C_{91}H_{96.5}AlBF_{20.5}MgN_4$	$C_{91}H_{96.5}BF_{20.5}GaMgN_4$
Formula weight	1697.82	1740.56
Temperature/K	100.0(6)	100.0(2)
Crystal system	orthorhombic	orthorhombic
Space group	Pcca	Pcca
a/Å	40.0598(5)	40.1582(8)
b/Å	20.7984(3)	20.7781(4)
c/Å	20.1956(3)	20.1828(4)
α/°	90	90
β/°	90	90
γ/°	90	90
Volume/Å^3	16826.6(4)	16840.7(6)
Z	8	8
ρ_{calc}g/cm^3	1.340	1.373
μ/mm^{-1}	1.094	1.329
F(000)	7080.0	7224.0
Crystal size/mm^3	0.307 × 0.107 × 0.047	0.103 × 0.095 × 0.052
Crystal color	yellow	yellow
Radiation	Cu Kα (λ = 1.54184)	Cu Kα (λ = 1.54184)
2Θ range for data collection/°	7.53 to 145.952	6.49 to 145.192
Index ranges	-34 ≤ h ≤ 48, -25 ≤ k ≤ 22, -19 ≤ l ≤ 24	-48 ≤ h ≤ 34, -25 ≤ k ≤ 16, -24 ≤ l ≤ 18
Reflections collected	66051	37374
Independent reflections	16394 [R_{int} = 0.0425, R_{sigma} = 0.0304]	16041 [R_{int} = 0.0331, R_{sigma} = 0.0372]
Data/restraints/parameters	16394/231/1185	16041/120/1173
Goodness-of-fit on F^2	1.028	1.033
Final R indexes [I>=2σ (I)]	R_1 = 0.0406, wR_2 = 0.1031	R_1 = 0.0618, wR_2 = 0.1600
Final R indexes [all data]	R_1 = 0.0487, wR_2 = 0.1093	R_1 = 0.0733, wR_2 = 0.1715
Largest diff. peak/hole / e Å^{-3}	0.31/-0.28	1.61/-1.34
CCDC Nr.	2067857	2067858

Table S2. Crystal data (continued).

Identification code	[(^{tBu}BDI)Zn-Ga(^{Me}BDI)$^+$][B(C_6F_5)$_4^-$] ·0.5 PhF	(^{tBu}BDI)ZnPh	(^{tBu}BDI)Zn(*p*-tolyl)
Empirical formula	$C_{91}H_{96.5}BF_{20.5}GaN_4Zn$	$C_{41}H_{58}N_2Zn$	$C_{42}H_{60}N_2Zn$
Formula weight	1781.62	644.26	658.29
Temperature/K	100.0(1)	100.0(1)	100.0(2)
Crystal system	orthorhombic	orthorhombic	monoclinic
Space group	Pcca	$Pca2_1$	$P2_1/c$
a/Å	40.3607(5)	17.2438(4)	16.9681(3)
b/Å	20.5547(3)	12.4223(3)	10.26658(13)
c/Å	20.1458(3)	17.3452(4)	21.8966(3)
α/°	90	90	90
β/°	90	90	96.4807(13)
γ/°	90	90	90
Volume/Å^3	16713.0(4)	3715.48(14)	3790.12(9)
Z	8	4	4
ρ_{calc}g/cm^3	1.416	1.152	1.154
μ/mm^{-1}	1.564	1.115	1.103
F(000)	7368.0	1392.0	1424.0
Crystal size/mm^3	0.244 × 0.151 × 0.034	0.28 × 0.199 × 0.098	0.329 × 0.169 × 0.122
Crystal color	yellow	colourless	colourless
Radiation	Cu Kα (λ = 1.54184)	Cu Kα (λ = 1.54184)	Cu Kα (λ = 1.54184)
2Θ range for data collection/°	6.138 to 144.68	7.116 to 145.186	8.128 to 145.71
Index ranges	-35 ≤ h ≤ 49, -22 ≤ k ≤ 24, -16 ≤ l ≤ 24	-18 ≤ h ≤ 21, -14 ≤ k ≤ 14, -21 ≤ l ≤ 12	-21 ≤ h ≤ 16, -12 ≤ k ≤ 9, -26 ≤ l ≤ 25
Reflections collected	49004	12401	21882
Independent reflections	16097 [R_{int} = 0.0267, R_{sigma} = 0.0269]	5079 [R_{int} = 0.0230, R_{sigma} = 0.0262]	7389 [R_{int} = 0.0258, R_{sigma} = 0.0248]
Data/restraints/parameters	16097/100/1181	5079/4/442	7389/113/492
Goodness-of-fit on F^2	1.024	1.050	1.029
Final R indexes [I>=2σ (I)]	R_1 = 0.0339, wR_2 = 0.0825	R_1 = 0.0238, wR_2 = 0.0618	R_1 = 0.0306, wR_2 = 0.0815
Final R indexes [all data]	R_1 = 0.0428, wR_2 = 0.0872	R_1 = 0.0248, wR_2 = 0.0629	R_1 = 0.0329, wR_2 = 0.0841
Largest diff. peak/hole / e Å^{-3}	0.38/-0.49	0.20/-0.28	0.29/-0.44
CCDC Nr.	2067859	2067860	2081483

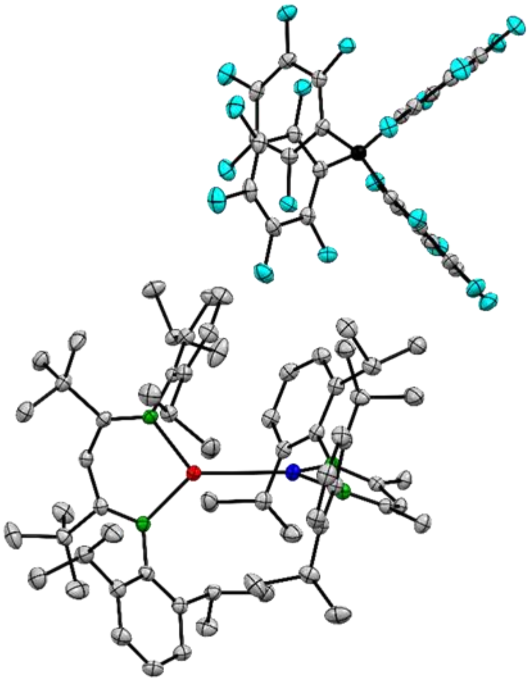

Figure S53 ORTEP representation of [(^{tBu}BDI)Mg-Al(^{Me}BDI)$^{+}$][B(C$_6$F$_5$)$_4$$^{-}$] (probability level 50%). Hydrogen atoms were omitted for clarity.

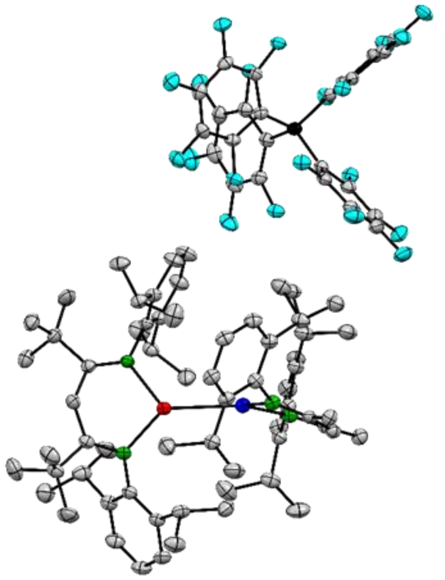

Figure S54 ORTEP representation of [(^{tBu}BDI)Mg-Ga(^{Me}BDI)$^{+}$][B(C$_6$F$_5$)$_4$$^{-}$] (probability level 50%). Hydrogen atoms were omitted for clarity.

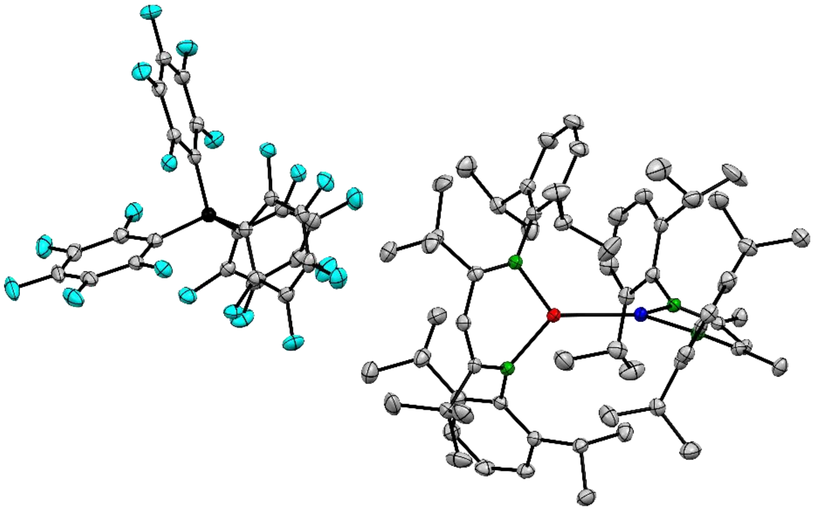

Figure S55 ORTEP representation of [(^{tBu}BDI)Zn-Ga(^{Me}BDI)$^{+}$][B(C_6F_5)$_4^{-}$] (probability level 50%). Hydrogen atoms were omitted for clarity.

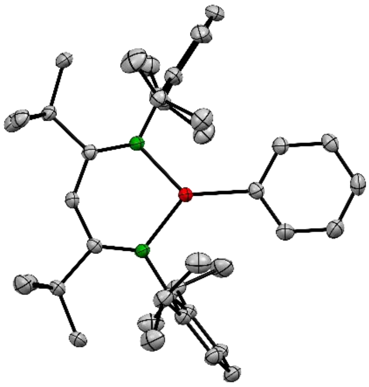

Figure S56 ORTEP representation of (^{tBu}BDI)ZnPh (probability level 50%). Hydrogen atoms were omitted for clarity.

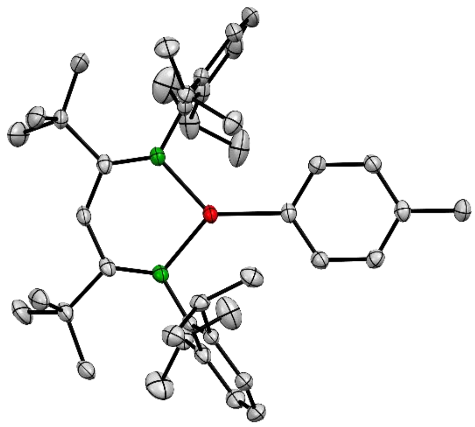

Figure S57 ORTEP representation of (^{tBu}BDI)Zn(*p*-tolyl) (probability level 50%). Hydrogen atoms were omitted for clarity.

5. Computational details

5.1 General methods

All geometry optimizations were carried out using Gaussian 16A.[S17] All methods were used as implemented. All structures were fully optimized on a ωB97XD/6-31+G** level of theory.[S18-20] All structures were characterized as true minima (Nimag=0) or transition states (Nimag=1) by frequency calculations on the same level of theory. Energies were determined at a ωB97XD/6-311+G** level of theory. Solvation effects were approximated using a PCM field of fluorobenzene (an energy profile without solvation corrections is shown in Figure S58).[S21] Charges were calculated via NBO analyses.[S22] All structures were evaluated using Molecule 2.3.[S23] Topological analyses were carried out with AIMAll17 using the wave function of the optimization.[S24-25]

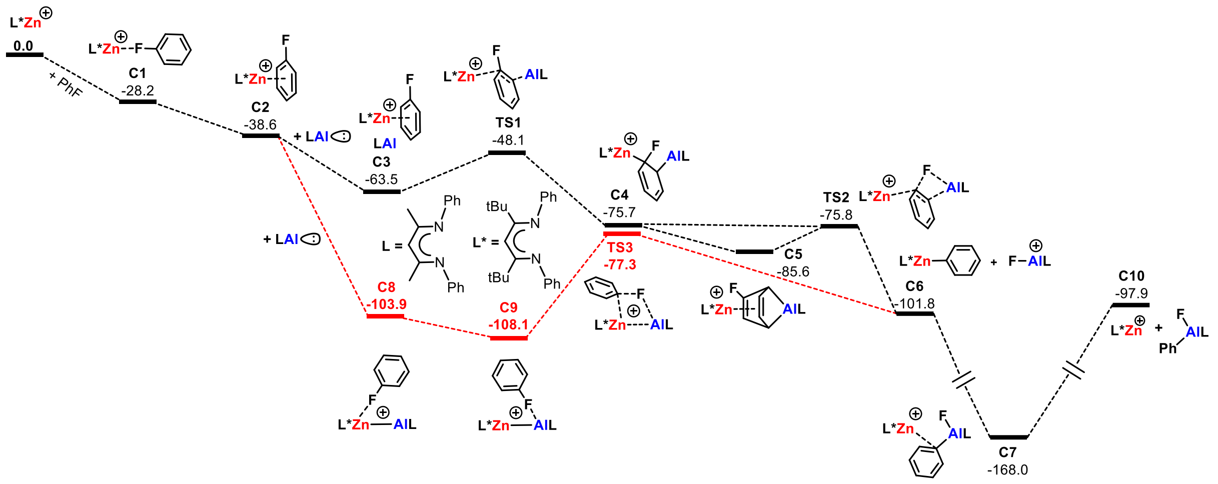

Figure S58 Energy profile for C-F bond activation in fluorobenzene by a combination of L*Zn$^+$ and LAl^I; ωB97XD/6-311+G**//ωB97XD/6-31+G** without solvent correction; ΔH in kcal/mol.

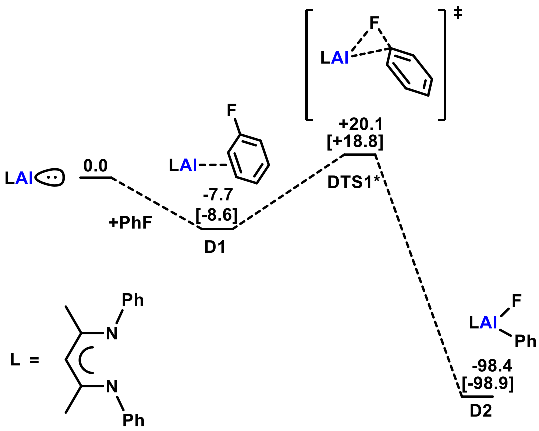

Figure S59 Energy profile for C-F bond activation in fluorobenzene by oxidative addition of LAl^{I}; ωB97XD/6-311+G**//ωB97XD/6-31+G** with solvent correction (PCM = fluorobenzene) and without solvent correction [brackets]; ΔH in kcal/mol. The C-F activation with only LAl^{I} (E_A= + 27.8 kcal/mol) shows higher activation energy than the combination of $L^{*}Zn^{+}$ and LAl^{I} (E_A= + 14.2 kcal/mol), which is not feasible at room temperature.

5.2 HOMO and LUMO energies and representations

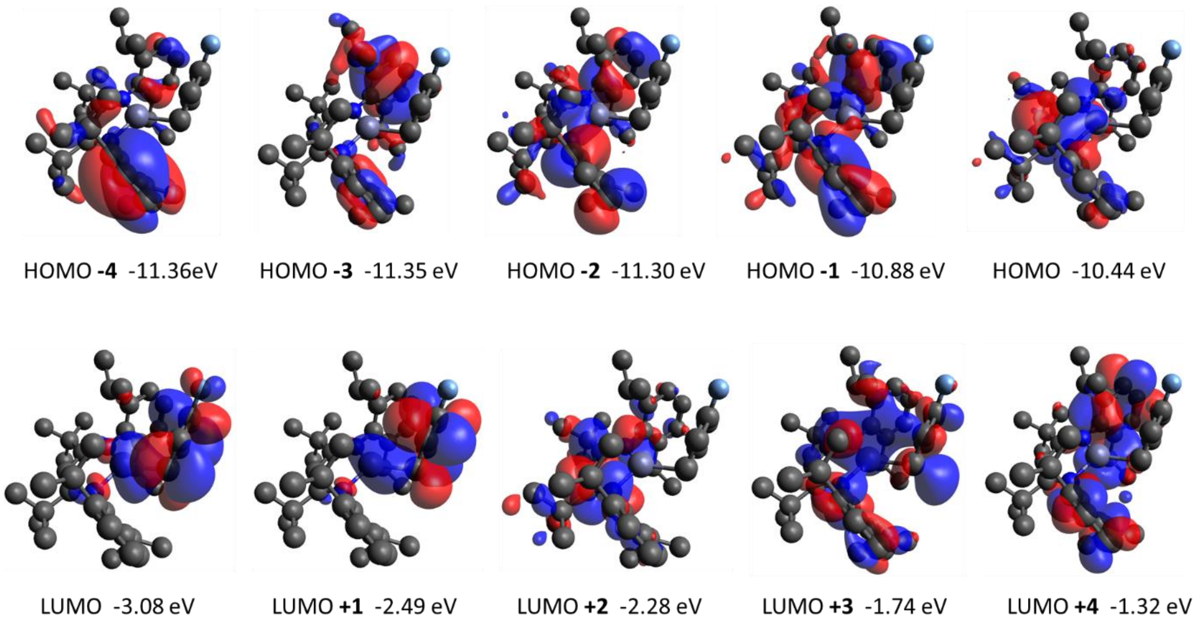

Figure S60 HOMO/LUMO energies and representations for $(^{tBu}BDI)Zn^{+}$···PhF calculated at the ωB97XD/6-311+G** level.

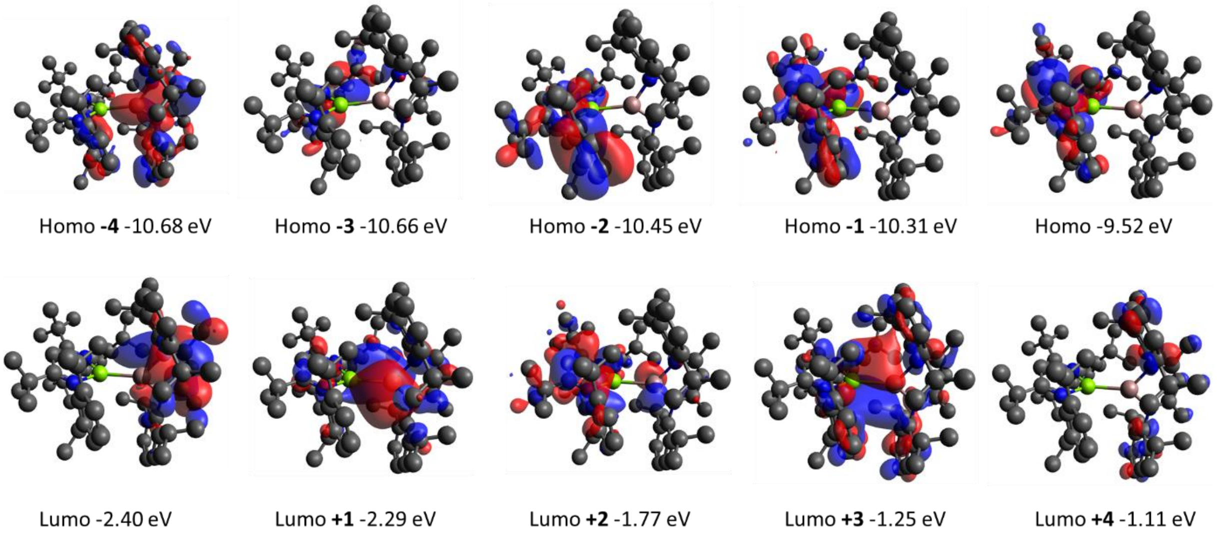

Figure S61 HOMO/LUMO energies and representations for (^{tBu}BDI)Mg^{+}-Ga(^{Me}BDI) calculated at the ωB97XD/6-311+G** level.

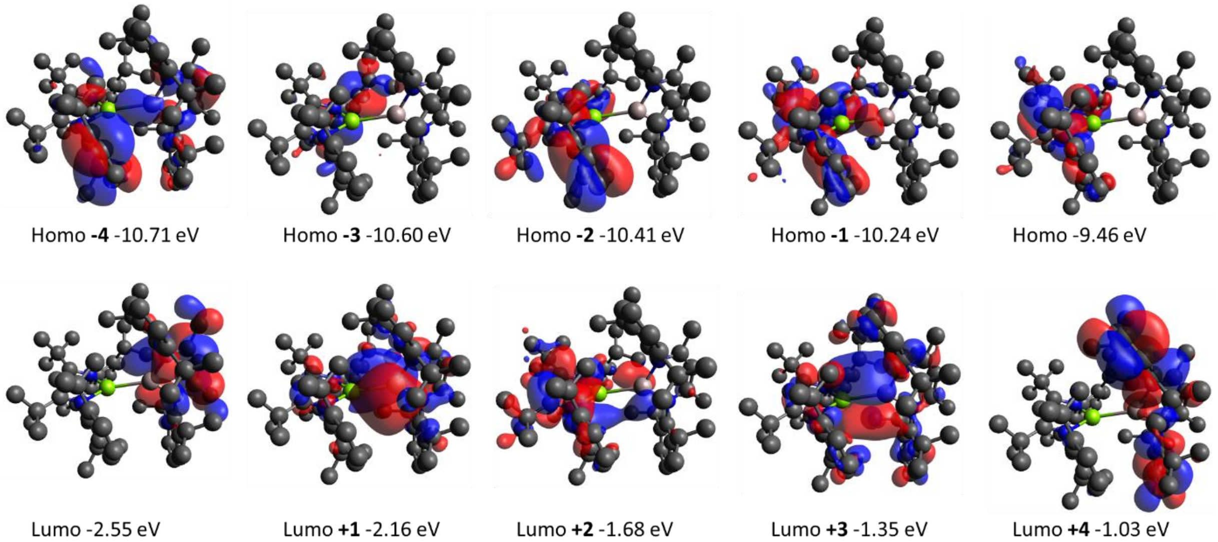

Figure S62 HOMO/LUMO energies and representations for (^{tBu}BDI)Mg^{+}-Al(^{Me}BDI) calculated at the ωB97XD/6-311+G** level.

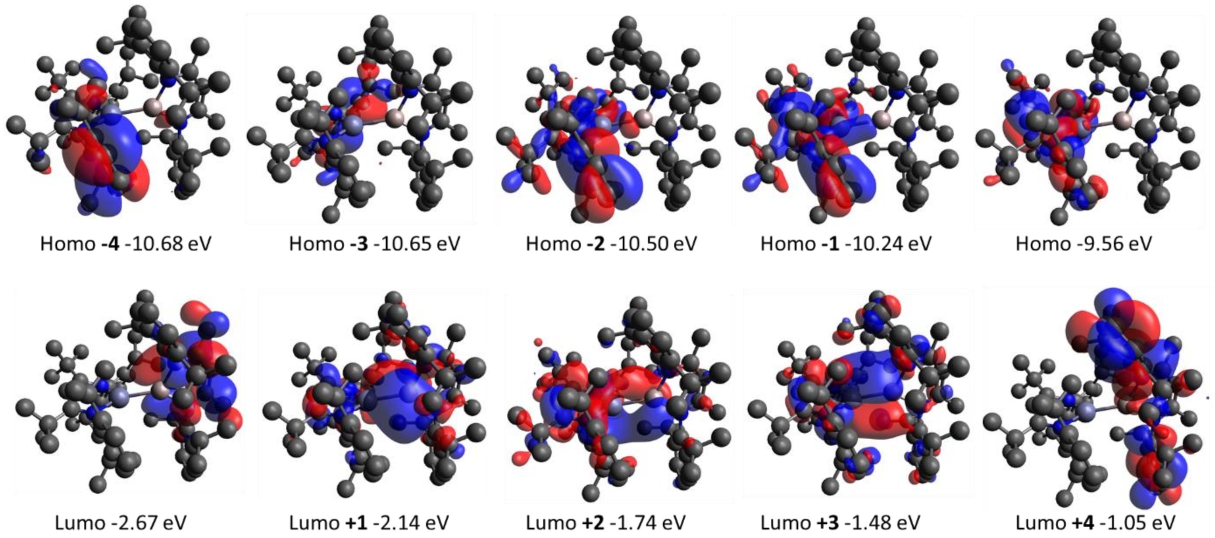

Figure S63 HOMO/LUMO energies and representations for (^{tBu}BDI)Zn^{+}-Al(^{Me}BDI) calculated at the ωB97XD/6-311+G** level.

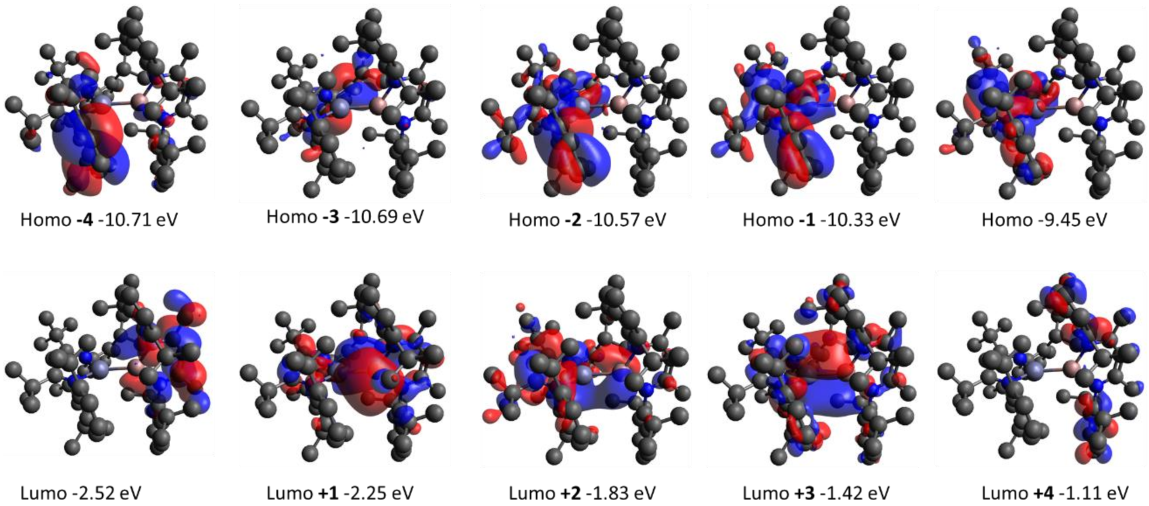

Figure S64 HOMO/LUMO energies and representations for (^{tBu}BDI)Zn^{+}-Ga(^{Me}BDI) calculated at the ωB97XD/6-311+G** level.

Table S2. Selected topological parameters for M^{I}-M^{II} complexes.

M^{II}-M^{I}	G(r)/a.u.	V(r)/a.u.	H(r)/a.u.	ρ(r)/e B^{-5}	H(r)*ρ(r)$^{-1}$/a.u.
Mg-Al	0.019	-0.028	-0.006	0.032	-0.170
Mg-Ga	0.023	-0.027	-0.005	0.033	-0.136
Zn-Al	0.011	-0.037	-0.026	0.061	-0.423
Zn-Ga	0.032	-0.059	-0.027	0.067	-0.402

5.3 X-Y-Z files

	91		
(^{tBu}BDI)Zn^{+}			
Zn	-0.000003	-0.878053	0.000151
N	1.535659	0.156963	0.170917
N	-1.535721	0.156803	-0.171379
C	1.283269	1.472193	0.265431
C	-0.000044	2.004758	-0.001211
C	-1.283366	1.471933	-0.267336
C	2.326807	2.523304	0.742276
C	3.733029	1.986247	1.053959
C	2.485519	3.653395	-0.296392
C	1.772438	3.107846	2.064939
C	-2.326864	2.522448	-0.745610
C	-1.772378	3.105177	-2.069034
C	-2.485757	3.654004	0.291430
C	-3.733046	1.984974	-1.056724
C	2.777193	-0.555287	0.150064
C	3.579414	-0.519437	-1.002874
C	4.727223	-1.312467	-1.017373
C	5.054027	-2.128495	0.060137
C	4.221622	-2.181536	1.171826
C	3.064555	-1.405402	1.231294
C	3.179112	0.326102	-2.203224
C	4.363653	0.796106	-3.050994
C	2.155746	-0.425795	-3.068636
C	2.093108	-1.501157	2.398266
C	2.753353	-1.808621	3.745697
C	1.019327	-2.563695	2.106317
C	-2.777240	-0.555432	-0.149368
C	-3.064666	-1.407156	-1.229329
C	-4.221760	-2.183148	-1.168708
C	-5.054124	-2.128455	-0.057072
C	-4.727216	-1.310931	1.019267
C	-3.579378	-0.517956	1.003602
C	-2.093201	-1.504742	-2.396131
C	-2.753374	-1.814754	-3.743006
C	-1.019237	-2.566603	-2.102296
C	-3.178986	0.329184	2.202796
C	-4.363445	0.800099	3.050174
C	-2.155374	-0.421411	3.069061
H	0.000009	3.080875	-0.001841
H	3.723020	1.180924	1.791337
H	4.317285	2.808739	1.476383
H	4.261486	1.634039	0.167312
H	2.852904	3.270372	-1.253040
H	3.219897	4.374104	0.074706
H	1.559617	4.201888	-0.482816
H	0.813068	3.612435	1.932920
H	2.484676	3.835942	2.464157
H	1.641452	2.320032	2.814371

H	-0.813108	3.610096	-1.937567
H	-2.484658	3.832595	-2.469408
H	-1.641145	2.316320	-2.817324
H	-2.853526	3.272378	1.248485
H	-3.219930	4.374278	-0.080924
H	-1.559869	4.202660	0.477419
H	-3.722986	1.178943	-1.793329
H	-4.317351	2.806999	-1.479998
H	-4.261496	1.633645	-0.169732
H	5.380817	-1.294219	-1.882561
H	5.955766	-2.730769	0.029100
H	4.482478	-2.831278	2.000398
H	2.680328	1.224690	-1.830024
H	5.127810	1.282514	-2.437216
H	4.021055	1.516132	-3.799614
H	4.835798	-0.030454	-3.591367
H	2.576345	-1.366698	-3.438365
H	1.856102	0.179720	-3.929405
H	1.248006	-0.658917	-2.498849
H	1.595729	-0.529981	2.500317
H	3.175288	-2.817639	3.778030
H	2.017132	-1.736686	4.551304
H	3.556944	-1.097560	3.954249
H	0.525518	-2.454259	1.107549
H	0.221319	-2.567526	2.852992
H	1.461848	-3.562597	2.047235
H	-4.482678	-2.834073	-1.996333
H	-5.955890	-2.730640	-0.025143
H	-5.380740	-1.291474	1.884482
H	-1.596023	-0.533647	-2.499895
H	-3.175385	-2.823800	-3.773385
H	-2.017124	-1.744409	-4.548726
H	-3.556917	-1.104045	-3.952940
H	-0.526038	-2.455959	-1.103307
H	-0.220858	-2.571267	-2.848568
H	-1.461527	-3.565541	-2.042074
H	-2.680418	1.227372	1.828371
H	-5.127839	1.285523	2.435911
H	-4.020825	1.521217	3.797733
H	-4.835294	-0.025816	3.591793
H	-2.575732	-1.361963	3.439955
H	-1.855873	0.185236	3.929085
H	-1.247582	-0.654956	2.499548

91

$(^{tBu}BDI)Mg^{+}$

C	-2.291610	2.484055	-0.253028
C	2.312557	1.760772	-1.709186
C	-2.556586	2.927181	-1.706183
C	1.642988	1.618065	-3.100893
C	-1.220353	1.341714	-0.195097
C	1.322419	1.102610	-0.694169

N	-1.459379	0.084904	0.220070
N	1.549925	-0.027325	-0.016509
Mg	0.066036	-0.966623	0.860451
C	0.064006	1.746552	-0.612331
C	-2.747320	-0.523290	0.259351
C	2.788385	-0.658094	0.304333
H	0.067420	2.726574	-1.058667
C	-1.715365	3.681116	0.546583
C	2.533939	3.270301	-1.467003
C	-3.661606	2.181386	0.380583
C	3.700851	1.109202	-1.788320
C	-3.307571	-0.836064	1.521280
C	3.576600	-0.148568	1.362627
C	-4.619935	-1.293875	1.584255
C	4.811773	-0.752080	1.612373
C	-5.365529	-1.482036	0.424830
C	5.230722	-1.875645	0.912806
C	-3.413820	-0.903382	-0.924749
C	3.115847	-1.902025	-0.279646
C	-4.745846	-1.327389	-0.803332
C	4.356086	-2.472345	0.015482
C	-2.522900	-0.762232	2.829534
C	3.129038	0.937416	2.341773
H	-5.068022	-1.501398	2.552047
H	5.446519	-0.343916	2.394009
H	-6.401061	-1.799240	0.482103
H	6.199001	-2.320212	1.115106
C	-2.827028	-1.065106	-2.336142
C	2.172717	-2.712810	-1.164744
H	-5.300520	-1.561188	-1.708431
H	4.633745	-3.408419	-0.461135
H	-3.194543	-1.181080	3.584236
H	3.855711	0.876875	3.159312
C	-2.144535	0.639326	3.326266
C	3.169990	2.377782	1.829253
C	-1.284205	-1.704188	2.826372
C	1.762192	0.644940	2.980550
H	-1.262614	-2.361270	3.698732
H	1.627253	1.236407	3.890884
H	-0.338663	-1.140730	2.936342
H	0.940411	0.931609	2.310216
H	-1.292527	-2.401100	1.968814
H	1.686104	-0.411751	3.271673
H	-1.622538	0.572337	4.286776
H	3.079690	3.074762	2.668294
H	-3.039502	1.246344	3.480243
H	4.111898	2.590036	1.316426
H	-1.498473	1.164009	2.617620
H	2.341083	2.577954	1.147709
H	-3.604230	-0.713152	-3.026264
H	2.661044	-3.686120	-1.275215
C	-2.649101	-2.576976	-2.591138

C	0.810731	-3.019979	-0.498439
C	-1.534189	-0.354053	-2.737373
C	1.959545	-2.157371	-2.574110
H	-2.311528	-2.756668	-3.616506
H	0.409797	-3.981446	-0.828961
H	-3.575744	-3.133888	-2.434485
H	0.909537	-3.120056	0.599375
H	-1.896966	-2.993500	-1.910211
H	0.038057	-2.297695	-0.811335
H	-1.309341	-0.602547	-3.779386
H	1.341411	-2.838585	-3.167784
H	-0.680600	-0.668930	-2.132658
H	1.456576	-1.190857	-2.536102
H	-1.596052	0.729637	-2.667441
H	2.914348	-2.029278	-3.089852
H	4.260265	1.196989	-0.855171
H	3.655001	0.054561	-2.063152
H	4.275042	1.624173	-2.564363
H	2.328149	1.993121	-3.867264
H	1.410699	0.579094	-3.343467
H	0.714943	2.190867	-3.166191
H	3.141094	3.460602	-0.582354
H	3.075199	3.677991	-2.325999
H	1.606643	3.839530	-1.374921
H	-3.578263	1.871088	1.422085
H	-4.245438	3.106596	0.363896
H	-4.235496	1.429686	-0.160087
H	-3.169678	3.833618	-1.697015
H	-1.639622	3.152876	-2.257223
H	-3.112839	2.162383	-2.255838
H	-1.468141	3.386309	1.571715
H	-0.819245	4.111319	0.096183
H	-2.468567	4.472654	0.599145

73

(^{Me}BDI)Ga

Ga	0.000028	0.000365	-0.908635
N	-1.396131	0.026297	0.568182
N	1.396132	-0.026459	0.568213
C	-2.477353	0.089246	2.766567
C	-1.250153	0.040455	1.888632
C	-0.000023	-0.000539	2.519934
C	1.250122	-0.041262	1.888648
C	2.477309	-0.090644	2.766557
C	-2.711753	0.053316	0.008475
C	-3.256278	1.283084	-0.400475
C	-4.509233	1.279669	-1.016025
C	-5.195263	0.092501	-1.238799
C	-4.629843	-1.116878	-0.850135
C	-3.381154	-1.161010	-0.230138
C	-2.508142	2.592162	-0.195617
C	-3.317716	3.595301	0.635894

C	-2.084118	3.204775	-1.538262
C	-2.750172	-2.493984	0.146046
C	-3.712055	-3.409530	0.912592
C	-2.192925	-3.202650	-1.097987
C	2.711744	-0.053311	0.008476
C	3.380997	1.161103	-0.230072
C	4.629656	1.117167	-0.850158
C	5.195191	-0.092111	-1.238948
C	4.509317	-1.279381	-1.016196
C	3.256403	-1.282984	-0.400577
C	2.749966	2.493987	0.146347
C	3.711636	3.409103	0.913686
C	2.193300	3.203226	-1.097614
C	2.508365	-2.592132	-0.195820
C	2.083763	-3.204253	-1.538523
C	3.318256	-3.595589	0.634981
H	-3.105154	0.944952	2.502315
H	-2.205817	0.157744	3.820596
H	-3.088998	-0.806053	2.617129
H	-0.000035	-0.000808	3.602391
H	3.089334	0.804402	2.617189
H	3.104730	-0.946597	2.502187
H	2.205779	-0.159163	3.820586
H	-4.950620	2.219378	-1.336617
H	-6.167078	0.107912	-1.722433
H	-5.166048	-2.041507	-1.043372
H	-1.593990	2.370722	0.362893
H	-4.230891	3.903342	0.115246
H	-2.724821	4.495802	0.826844
H	-3.611532	3.170339	1.600914
H	-1.503203	2.490710	-2.130348
H	-1.466900	4.094570	-1.373224
H	-2.957431	3.501521	-2.129853
H	-1.901798	-2.287689	0.804905
H	-4.544402	-3.742108	0.283304
H	-4.135951	-2.904305	1.786403
H	-3.185155	-4.304844	1.258001
H	-1.667679	-4.120935	-0.813410
H	-1.490344	-2.561707	-1.640876
H	-3.001518	-3.469181	-1.787841
H	5.165721	2.041885	-1.043371
H	6.166967	-0.107377	-1.722664
H	4.950800	-2.219014	-1.336877
H	1.901294	2.287507	0.804763
H	4.544294	3.741819	0.284881
H	4.135100	2.903471	1.787468
H	3.184712	4.304349	1.259238
H	1.668020	4.121437	-0.812855
H	1.490871	2.562567	-1.641037
H	3.002175	3.469988	-1.787046
H	1.594431	-2.370853	0.363110
H	1.466635	-4.094126	-1.373561

H	1.502586	-2.489980	-2.130107
H	2.956840	-3.500737	-2.130592
H	2.725299	-4.496006	0.826134
H	4.231027	-3.903744	0.113691
H	3.612805	-3.170887	1.599893

73
(^{Me}BDI)Al

Al	-0.000002	-0.224876	-0.978596
N	-1.389161	-0.137133	0.451449
N	1.389159	-0.137144	0.451450
C	-0.000002	-0.204400	2.408410
H	-0.000002	-0.231891	3.490488
C	-1.249421	-0.160683	1.777638
C	-2.714730	-0.010850	-0.092265
C	1.249418	-0.160697	1.777638
C	2.714731	-0.010874	-0.092261
C	-3.217721	1.274513	-0.367307
C	-3.453079	-1.166898	-0.404298
C	-2.474853	-0.124989	2.657682
H	-3.053317	0.784734	2.472344
H	-2.201545	-0.161352	3.712738
H	-3.134642	-0.967624	2.432232
C	2.474850	-0.125011	2.657684
H	3.134667	-0.967614	2.432193
H	2.201543	-0.161431	3.712739
H	3.053284	0.784740	2.472390
C	-2.422910	2.534595	-0.053604
H	-1.526942	2.239478	0.500519
C	3.217753	1.274488	-0.367260
C	3.453053	-1.166928	-0.404337
C	-5.218530	0.249672	-1.277963
H	-6.193459	0.350735	-1.744691
C	-4.476113	1.380254	-0.961984
H	-4.879448	2.363272	-1.188299
C	-4.706280	-1.010951	-0.999188
H	-5.289216	-1.891161	-1.255028
C	2.422968	2.534575	-0.053514
H	1.527023	2.239462	0.500649
C	-3.208479	3.507596	0.835908
H	-4.089481	3.903729	0.319823
H	-2.578143	4.358626	1.113695
H	-3.551330	3.023639	1.755935
C	4.706256	-1.010990	-0.999225
H	5.289170	-1.891206	-1.255095
C	1.957148	3.223888	-1.343879
H	2.813062	3.558577	-1.940435
H	1.343165	4.100359	-1.110441
H	1.363595	2.542016	-1.961101
C	-1.957145	3.223900	-1.343993
H	-1.363629	2.542020	-1.961240
H	-1.343140	4.100364	-1.110586

H	-2.813082	3.558600	-1.940510
C	4.476143	1.380218	-0.961938
H	4.879502	2.363235	-1.188222
C	-2.914066	-2.563830	-0.133003
H	-1.995620	-2.461886	0.452765
C	5.218533	0.249630	-1.277958
H	6.193463	0.350685	-1.744686
C	2.914011	-2.563857	-0.133082
H	1.995547	-2.461910	0.452658
C	-2.545971	-3.271173	-1.445399
H	-3.432880	-3.414192	-2.072845
H	-2.112176	-4.256149	-1.241825
H	-1.819872	-2.685235	-2.017505
C	2.545952	-3.271175	-1.445501
H	1.819883	-2.685218	-2.017625
H	2.112132	-4.256147	-1.241957
H	3.432880	-3.414203	-2.072917
C	3.208582	3.507568	0.835965
H	3.551489	3.023601	1.755967
H	2.578258	4.358592	1.113797
H	4.089553	3.903713	0.319835
C	3.890284	-3.414400	0.690959
H	4.806265	-3.632509	0.131728
H	3.428462	-4.372118	0.951902
H	4.179988	-2.911801	1.619314
C	-3.890376	-3.414349	0.691019
H	-4.180120	-2.911723	1.619347
H	-3.428567	-4.372061	0.952007
H	-4.806332	-3.632473	0.131755

164

[(^{tBu}BDI)Mg-Al(^{Me}BDI)$^{+}$]

Al	1.630636	0.519384	0.449007
Mg	-0.934251	-0.149215	-0.074536
N	-2.648481	0.306969	0.879649
N	-1.608335	-1.653390	-1.245406
N	2.776702	-0.029947	1.851852
N	2.631054	2.032407	-0.093866
C	-3.534337	-0.713629	0.994060
C	-3.556955	-1.806437	0.120564
C	-2.808703	-2.165825	-1.037454
C	-4.638542	-0.764663	2.103371
C	-4.275425	0.055439	3.350471
C	-5.989644	-0.270997	1.555604
C	-4.815936	-2.220778	2.602138
C	-3.570804	-3.101889	-2.019984
C	-4.682894	-2.214916	-2.629974
C	-4.208635	-4.299870	-1.282878
C	-2.761455	-3.687497	-3.189197
C	-3.003559	1.653895	1.215642
C	-3.979775	2.310652	0.423558
C	-4.342183	3.619043	0.743179

C	-3.736901	4.303853	1.789504
C	-2.725465	3.682833	2.505426
C	-2.336265	2.369364	2.232305
C	-4.540189	1.671319	-0.842739
C	-5.964222	2.103768	-1.206862
C	-3.587688	1.986031	-2.003733
C	-1.197050	1.780263	3.047939
C	-1.429103	1.860267	4.562667
C	0.124310	2.482098	2.704623
C	-0.619138	-2.067995	-2.188578
C	-0.334898	-1.255265	-3.298255
C	0.757206	-1.582426	-4.104906
C	1.547736	-2.686912	-3.826011
C	1.240186	-3.495501	-2.735601
C	0.162342	-3.208366	-1.900239
C	-1.213764	-0.068831	-3.650644
C	-1.798593	-0.175101	-5.064931
C	-0.460891	1.254188	-3.475842
C	-0.180823	-4.105928	-0.719197
C	0.259477	-5.560727	-0.903427
C	0.386913	-3.542959	0.585379
C	4.450384	0.338225	3.618859
C	3.659961	0.805139	2.427179
C	3.921461	2.091493	1.942132
C	3.525682	2.630159	0.709810
C	4.164757	3.923613	0.280621
C	2.819209	-1.426376	2.245596
C	1.960809	-1.929368	3.239732
C	2.091885	-3.272298	3.602995
C	3.033962	-4.097293	3.008489
C	3.855703	-3.589446	2.012442
C	3.762948	-2.257214	1.608360
C	0.915962	-1.088155	3.957699
C	1.199028	-1.007933	5.466164
C	-0.501322	-1.630603	3.719276
C	4.660595	-1.774547	0.478341
C	6.150317	-1.981962	0.781553
C	4.267968	-2.466670	-0.834121
C	2.424500	2.587000	-1.418292
C	1.575464	3.693375	-1.595908
C	1.457559	4.236442	-2.876911
C	2.159759	3.707262	-3.947717
C	2.964204	2.590786	-3.760712
C	3.102105	1.996026	-2.505430
C	0.738217	4.275183	-0.468792
C	0.748866	5.808460	-0.424735
C	-0.699873	3.753390	-0.580793
C	3.966453	0.750028	-2.341739
C	4.140648	-0.028973	-3.649152
C	5.344456	1.067423	-1.741644
H	-4.387960	-2.464214	0.295268
H	-3.270656	-0.198101	3.698828

H	-4.977783	-0.186763	4.153323
H	-4.328689	1.130564	3.188065
H	-5.969213	0.803946	1.366267
H	-6.775465	-0.465039	2.293073
H	-6.260792	-0.784653	0.627619
H	-5.300696	-2.875676	1.874797
H	-5.456222	-2.213634	3.488891
H	-3.855890	-2.667788	2.878880
H	-5.356353	-1.824836	-1.862405
H	-5.272629	-2.800326	-3.342629
H	-4.249310	-1.364240	-3.168168
H	-3.463138	-4.857458	-0.706754
H	-4.638304	-4.983306	-2.020839
H	-5.018256	-4.014130	-0.608191
H	-2.300961	-2.924740	-3.815893
H	-3.451149	-4.255304	-3.821278
H	-1.980912	-4.376145	-2.859679
H	-5.104816	4.119853	0.156367
H	-4.038703	5.318048	2.029797
H	-2.231313	4.229249	3.303767
H	-4.554860	0.586948	-0.718540
H	-6.656365	1.990822	-0.368116
H	-6.333019	1.487770	-2.032807
H	-6.005460	3.145512	-1.541227
H	-3.574909	3.061835	-2.210908
H	-3.889853	1.462521	-2.917395
H	-2.563408	1.688916	-1.763187
H	-1.111229	0.724246	2.775520
H	-1.440496	2.898277	4.909143
H	-0.618430	1.351589	5.093530
H	-2.374930	1.402291	4.857951
H	0.336198	2.412026	1.632531
H	0.965925	2.056245	3.262107
H	0.078783	3.550304	2.941435
H	0.979733	-0.968344	-4.973471
H	2.393060	-2.930094	-4.461904
H	1.850878	-4.370641	-2.541066
H	-2.059107	-0.077042	-2.957545
H	-1.016413	-0.128611	-5.829552
H	-2.490311	0.653515	-5.248476
H	-2.346894	-1.111416	-5.203366
H	-0.061705	1.371545	-2.460742
H	-1.112488	2.109554	-3.679556
H	0.396298	1.313458	-4.153364
H	-1.270670	-4.116191	-0.618430
H	-0.086611	-5.970556	-1.857233
H	-0.154441	-6.176037	-0.099181
H	1.348915	-5.666036	-0.861647
H	1.479199	-3.496336	0.547999
H	0.101718	-4.163381	1.440051
H	0.017336	-2.532092	0.780912
H	3.798428	-0.107772	4.372226

H	5.005083	1.163471	4.064364
H	5.160769	-0.438228	3.320646
H	4.620268	2.684220	2.517088
H	3.421055	4.725547	0.261656
H	4.569285	3.842062	-0.731338
H	4.964564	4.206924	0.963878
H	1.444771	-3.679531	4.373872
H	3.121892	-5.134744	3.313857
H	4.581114	-4.242301	1.536735
H	0.960487	-0.071837	3.556455
H	1.141958	-1.995907	5.933105
H	0.461956	-0.368922	5.960936
H	2.191646	-0.600457	5.676296
H	-0.779868	-1.606194	2.660862
H	-1.232752	-1.035821	4.273976
H	-0.593519	-2.667440	4.056712
H	4.510909	-0.699431	0.344562
H	6.403639	-3.043827	0.858033
H	6.443441	-1.500603	1.719080
H	6.760615	-1.554938	-0.020147
H	4.914056	-2.131203	-1.650118
H	3.228711	-2.259216	-1.112494
H	4.374689	-3.553423	-0.753212
H	0.806004	5.089271	-3.036752
H	2.067822	4.151395	-4.933544
H	3.484149	2.172454	-4.614177
H	1.149903	3.926829	0.482092
H	0.211803	6.245507	-1.271950
H	1.767060	6.209476	-0.433771
H	0.252133	6.155314	0.486138
H	-1.333646	4.140723	0.220964
H	-0.726406	2.658985	-0.521873
H	-1.141656	4.034327	-1.542044
H	3.446225	0.077200	-1.645165
H	4.598132	-1.000016	-3.446012
H	3.181563	-0.209639	-4.139612
H	4.800258	0.496995	-4.346859
H	5.953050	0.158567	-1.694972
H	5.873527	1.793665	-2.367581
H	5.279808	1.470917	-0.728425

164

[(^{tBu}BDI)Mg-Ga(^{Me}BDI)$^+$]

Ga	-1.513275	0.419463	-0.451296
Mg	0.984720	-0.143402	0.115705
N	2.686427	0.245091	-0.865113
N	1.640595	-1.527256	1.415885
N	-2.656811	-0.212314	-1.876804
N	-2.558107	1.989664	-0.031682
C	3.579778	-0.772521	-0.880526
C	3.604521	-1.784679	0.087018
C	2.843519	-2.055041	1.259187

C	4.678235	-0.934607	-1.983695
C	4.464547	-0.055011	-3.224266
C	6.079942	-0.645794	-1.417691
C	4.626973	-2.395168	-2.500717
C	3.587626	-2.922413	2.314054
C	4.745219	-2.031840	2.825975
C	4.162364	-4.209869	1.685340
C	2.770183	-3.353002	3.542256
C	3.036183	1.560929	-1.307283
C	4.030629	2.271199	-0.587862
C	4.385204	3.550842	-1.014152
C	3.754835	4.156176	-2.094565
C	2.728282	3.484617	-2.739727
C	2.346769	2.195738	-2.360403
C	4.623093	1.722789	0.706288
C	6.028281	2.236266	1.034303
C	3.663606	2.046082	1.858548
C	1.197674	1.540928	-3.108375
C	1.411718	1.507712	-4.627375
C	-0.126617	2.252270	-2.803664
C	0.623391	-1.873087	2.357530
C	0.308311	-0.982955	3.396955
C	-0.825871	-1.233092	4.171535
C	-1.619709	-2.346489	3.939890
C	-1.271640	-3.241354	2.933136
C	-0.156573	-3.026338	2.125731
C	1.189813	0.215341	3.696107
C	1.631244	0.275969	5.163229
C	0.492455	1.515226	3.286168
C	0.226081	-4.012055	1.031990
C	-0.183491	-5.455807	1.335017
C	-0.328672	-3.576693	-0.325374
C	-4.315702	0.041728	-3.663072
C	-3.532184	0.585968	-2.499914
C	-3.806838	1.896597	-2.094953
C	-3.434997	2.515644	-0.893943
C	-4.092779	3.829400	-0.566068
C	-2.694233	-1.627476	-2.170439
C	-1.816411	-2.201633	-3.103846
C	-1.946098	-3.565552	-3.377168
C	-2.909956	-4.341440	-2.752323
C	-3.754810	-3.760687	-1.816388
C	-3.660952	-2.404809	-1.504464
C	-0.753676	-1.408638	-3.846387
C	-0.969973	-1.469289	-5.365769
C	0.655875	-1.901944	-3.487912
C	-4.546844	-1.837430	-0.406980
C	-6.032371	-2.156353	-0.606404
C	-4.043021	-2.354003	0.946167
C	-2.373204	2.636412	1.247704
C	-1.522989	3.749714	1.355366
C	-1.431348	4.396307	2.589319

C	-2.167291	3.964141	3.680825
C	-2.976035	2.840645	3.564876
C	-3.084702	2.143310	2.360809
C	-0.671082	4.239157	0.196634
C	-0.677421	5.764103	0.035469
C	0.764323	3.725886	0.355003
C	-3.953171	0.893834	2.271732
C	-4.204927	0.244099	3.635563
C	-5.298830	1.156868	1.579125
H	4.445841	-2.446433	-0.021437
H	3.445769	-0.162860	-3.603102
H	5.147984	-0.388760	-4.010774
H	4.663294	1.001529	-3.048791
H	6.193909	0.413362	-1.175489
H	6.836000	-0.898381	-2.168187
H	6.290280	-1.230181	-0.516988
H	4.930013	-3.133324	-1.756066
H	5.308374	-2.497591	-3.350563
H	3.618472	-2.652935	-2.840640
H	5.431073	-1.751803	2.022560
H	5.312585	-2.569309	3.592372
H	4.355521	-1.111871	3.276503
H	3.377359	-4.809350	1.213435
H	4.616119	-4.817967	2.473377
H	4.939615	-4.020528	0.942091
H	2.353322	-2.508027	4.090340
H	3.442327	-3.883772	4.223425
H	1.955122	-4.034593	3.291961
H	5.161195	4.094051	-0.486069
H	4.050654	5.149230	-2.416573
H	2.216176	3.970651	-3.565228
H	4.687928	0.635485	0.639369
H	6.715673	2.119711	0.191359
H	6.434919	1.674439	1.880640
H	6.024332	3.292638	1.322385
H	3.584988	3.129062	2.004389
H	4.004876	1.594772	2.796596
H	2.659972	1.668958	1.646701
H	1.124741	0.508518	-2.755866
H	1.438197	2.518317	-5.046072
H	0.586298	0.979400	-5.113093
H	2.346416	1.014268	-4.901639
H	-0.323072	2.272370	-1.727519
H	-0.968596	1.762853	-3.304937
H	-0.102271	3.295543	-3.135211
H	-1.077737	-0.552272	4.980007
H	-2.498412	-2.529015	4.550235
H	-1.883112	-4.123658	2.776926
H	2.096305	0.106618	3.094339
H	0.784853	0.451368	5.834902
H	2.341081	1.096915	5.306665
H	2.118724	-0.652585	5.474143

H	0.182414	1.495904	2.234597
H	1.143744	2.383136	3.426222
H	-0.419850	1.673234	3.868823
H	1.316746	-4.005292	0.950676
H	0.159180	-5.773498	2.324499
H	0.254501	-6.128288	0.591430
H	-1.269520	-5.590224	1.290683
H	-1.422242	-3.599973	-0.327743
H	0.026887	-4.232485	-1.125423
H	-0.019526	-2.558252	-0.578915
H	-3.670405	-0.498827	-4.358079
H	-4.828268	0.843325	-4.194636
H	-5.065676	-0.672026	-3.308010
H	-4.499595	2.448299	-2.716093
H	-3.357291	4.638732	-0.595567
H	-4.510653	3.817736	0.443351
H	-4.886073	4.054575	-1.277954
H	-1.280849	-4.028815	-4.099482
H	-2.997435	-5.397259	-2.986675
H	-4.493552	-4.375583	-1.311837
H	-0.835698	-0.363799	-3.534540
H	-0.882584	-2.494234	-5.738749
H	-0.219550	-0.868258	-5.886766
H	-1.958325	-1.097150	-5.650237
H	0.875713	-1.762005	-2.424487
H	1.411901	-1.357326	-4.061653
H	0.772994	-2.967564	-3.707909
H	-4.451217	-0.747685	-0.401869
H	-6.233786	-3.229185	-0.529910
H	-6.388938	-1.817640	-1.583732
H	-6.630160	-1.656760	0.162019
H	-4.642691	-1.949540	1.765567
H	-2.997020	-2.077668	1.118649
H	-4.102783	-3.446448	0.994638
H	-0.779010	5.257215	2.693487
H	-2.099919	4.489704	4.627786
H	-3.527149	2.502439	4.434212
H	-1.076949	3.819289	-0.726561
H	-0.144681	6.263804	0.850249
H	-1.694708	6.166332	0.007480
H	-0.174341	6.039550	-0.896161
H	1.404995	4.066104	-0.462006
H	0.788521	2.630209	0.356050
H	1.198062	4.059070	1.302874
H	-3.402497	0.159630	1.668330
H	-4.669585	-0.736065	3.502114
H	-3.276728	0.099045	4.192230
H	-4.890776	0.840715	4.245809
H	-5.904274	0.244803	1.581064
H	-5.858048	1.931134	2.114870
H	-5.187809	1.471230	0.539628

164

[(^{tBu}BDI)Zn-Al(^{Me}BDI)$^{+}$]

Al	1.475806	0.525359	0.522439
Zn	-0.821597	-0.155055	-0.049273
N	-2.512096	0.223374	0.861520
N	-1.398196	-1.536677	-1.338221
N	2.577729	-0.126280	1.910101
N	2.506817	2.041579	0.067965
C	-3.397949	-0.801296	0.881533
C	-3.385881	-1.830867	-0.060503
C	-2.601905	-2.076286	-1.217721
C	-4.531600	-0.944851	1.950495
C	-4.374823	-0.038570	3.179839
C	-5.912975	-0.680143	1.326628
C	-4.489917	-2.394594	2.498901
C	-3.333981	-2.915991	-2.308407
C	-4.388101	-1.950200	-2.902414
C	-4.048347	-4.139552	-1.690673
C	-2.494614	-3.454676	-3.480447
C	-2.913486	1.546089	1.248992
C	-3.898290	2.205734	0.471406
C	-4.322952	3.476258	0.862285
C	-3.773566	4.119225	1.963879
C	-2.758153	3.495296	2.671924
C	-2.311123	2.217569	2.331621
C	-4.415343	1.626214	-0.841724
C	-5.843067	2.044644	-1.209745
C	-3.451677	2.028774	-1.964709
C	-1.187560	1.610195	3.151155
C	-1.451972	1.619757	4.662555
C	0.129662	2.342303	2.870316
C	-0.398953	-1.890220	-2.299008
C	-0.121765	-1.027069	-3.370516
C	0.965126	-1.319646	-4.196211
C	1.748874	-2.442853	-3.977222
C	1.430251	-3.313305	-2.939371
C	0.355791	-3.063202	-2.086832
C	-1.022512	0.153851	-3.683556
C	-1.582022	0.097493	-5.111234
C	-0.314354	1.487517	-3.437774
C	-0.033120	-4.051496	-0.994534
C	0.421837	-5.486357	-1.274117
C	0.463530	-3.598549	0.377884
C	4.130711	0.175492	3.797153
C	3.423352	0.687186	2.572134
C	3.705323	1.993145	2.162802
C	3.371728	2.589360	0.938921
C	4.042815	3.894594	0.604331
C	2.657605	-1.543078	2.223277
C	1.800275	-2.132292	3.168495
C	1.976242	-3.487155	3.460674
C	2.972929	-4.236956	2.855701

C	3.804141	-3.639811	1.918928
C	3.660366	-2.294465	1.578177
C	0.710380	-1.370458	3.905269
C	0.892189	-1.452036	5.429032
C	-0.685316	-1.880350	3.517050
C	4.581016	-1.710663	0.516731
C	6.060949	-1.825477	0.905514
C	4.313434	-2.375328	-0.840062
C	2.371583	2.681581	-1.228473
C	1.549628	3.813221	-1.366193
C	1.504907	4.446838	-2.609357
C	2.259360	3.985908	-3.676364
C	3.046022	2.851412	-3.527100
C	3.108958	2.165093	-2.313288
C	0.674683	4.332946	-0.237479
C	0.672905	5.861974	-0.117624
C	-0.753487	3.805235	-0.414245
C	3.967018	0.910359	-2.189995
C	4.224104	0.229863	-3.538156
C	5.308710	1.190006	-1.495234
H	-4.219111	-2.502395	0.035160
H	-3.369104	-0.122767	3.596392
H	-5.080296	-0.368674	3.948160
H	-4.584540	1.010731	2.976473
H	-6.022827	0.372504	1.058129
H	-6.694779	-0.921815	2.053980
H	-6.085480	-1.284837	0.431310
H	-4.761048	-3.149865	1.759122
H	-5.202864	-2.483798	3.323907
H	-3.494228	-2.639494	2.882171
H	-5.079247	-1.586859	-2.136996
H	-4.967422	-2.467177	-3.674040
H	-3.905661	-1.083271	-3.367034
H	-3.359092	-4.748252	-1.096485
H	-4.436772	-4.767831	-2.496882
H	-4.900890	-3.873616	-1.062102
H	-1.983647	-2.674451	-4.040965
H	-3.177725	-3.957007	-4.172143
H	-1.752676	-4.190751	-3.165131
H	-5.092109	3.981204	0.288101
H	-4.123742	5.103898	2.255812
H	-2.311589	4.007836	3.519096
H	-4.405932	0.536596	-0.781850
H	-6.546530	1.893083	-0.386379
H	-6.188510	1.450784	-2.061534
H	-5.898343	3.096241	-1.509569
H	-3.452267	3.115867	-2.100860
H	-3.737772	1.563219	-2.914256
H	-2.429037	1.726408	-1.729450
H	-1.082072	0.570931	2.832776
H	-1.478532	2.639722	5.058124
H	-0.648189	1.092136	5.185395

H	-2.399792	1.142100	4.918670
H	0.357010	2.339362	1.797895
H	0.965826	1.896795	3.418807
H	0.067350	3.396249	3.161226
H	1.185345	-0.666515	-5.036294
H	2.591356	-2.657421	-4.627071
H	2.025249	-4.210009	-2.804335
H	-1.875166	0.092989	-3.004077
H	-0.792404	0.215622	-5.860332
H	-2.302377	0.908346	-5.261463
H	-2.092565	-0.849174	-5.310130
H	0.040854	1.572675	-2.405815
H	-0.985980	2.329935	-3.631111
H	0.559388	1.595813	-4.087278
H	-1.126771	-4.073662	-0.949113
H	0.118336	-5.822603	-2.270367
H	-0.022032	-6.162869	-0.537851
H	1.509222	-5.592030	-1.194485
H	1.555889	-3.569309	0.401692
H	0.124545	-4.281128	1.162686
H	0.094359	-2.601106	0.628851
H	3.409411	-0.216818	4.517890
H	4.712319	0.967099	4.268167
H	4.800589	-0.650318	3.543667
H	4.371760	2.558761	2.799812
H	3.310698	4.707309	0.602443
H	4.485336	3.864008	-0.394039
H	4.818889	4.126968	1.332557
H	1.323202	-3.962807	4.185858
H	3.097598	-5.284787	3.108212
H	4.576586	-4.232311	1.438259
H	0.780652	-0.317343	3.618569
H	0.772707	-2.477899	5.790158
H	0.140526	-0.840038	5.935616
H	1.880429	-1.105838	5.744754
H	-0.894608	-1.741939	2.452077
H	-1.456123	-1.346854	4.082009
H	-0.789042	-2.947814	3.736677
H	4.363284	-0.644585	0.410314
H	6.378125	-2.869976	0.982917
H	6.264462	-1.341862	1.865472
H	6.688813	-1.345151	0.148949
H	4.981483	-1.965848	-1.602934
H	3.280762	-2.223014	-1.172024
H	4.488268	-3.455153	-0.794091
H	0.873068	5.318786	-2.741430
H	2.224515	4.500202	-4.631247
H	3.611816	2.492053	-4.378227
H	1.061658	3.942502	0.707451
H	0.149815	6.337067	-0.953001
H	1.687602	6.270934	-0.083768
H	0.153184	6.158749	0.798067

H	-1.411939	4.160331	0.382518
H	-0.777896	2.710678	-0.399550
H	-1.166346	4.125880	-1.376057
H	3.412098	0.187674	-1.574637
H	4.659412	-0.759437	-3.378654
H	3.300301	0.097875	-4.104669
H	4.933117	0.800059	-4.147163
H	5.927813	0.287499	-1.495636
H	5.856972	1.972043	-2.030922
H	5.190177	1.504903	-0.455858

164

[(^{tBu}BDI)Zn-Ga(^{Me}BDI)$^{+}$]

Ga	-1.361276	0.447296	-0.533176
Zn	0.867535	-0.176262	0.065767
N	2.552263	0.158257	-0.843682
N	1.408199	-1.457774	1.447631
N	-2.493124	-0.274928	-1.917773
N	-2.431911	2.008668	-0.161722
C	3.440518	-0.862916	-0.783977
C	3.416013	-1.829724	0.222228
C	2.613884	-2.003795	1.379822
C	4.579578	-1.077780	-1.834412
C	4.455099	-0.226592	-3.106353
C	5.964175	-0.818736	-1.214899
C	4.502476	-2.551321	-2.311159
C	3.323969	-2.778396	2.529752
C	4.387789	-1.791670	3.069490
C	4.023764	-4.050873	2.000838
C	2.464120	-3.222623	3.725746
C	2.955286	1.454179	-1.309249
C	3.946560	2.156215	-0.579271
C	4.367346	3.401007	-1.048673
C	3.806135	3.979067	-2.180132
C	2.783420	3.316029	-2.840716
C	2.340518	2.060504	-2.421849
C	4.478178	1.651672	0.758847
C	5.898906	2.114741	1.097988
C	3.511555	2.085837	1.866834
C	1.208858	1.403662	-3.189648
C	1.469143	1.309407	-4.698295
C	-0.106571	2.152395	-2.953665
C	0.388379	-1.744277	2.409453
C	0.099851	-0.814715	3.419944
C	-1.010967	-1.041266	4.233446
C	-1.797691	-2.172375	4.071540
C	-1.460054	-3.113460	3.104401
C	-0.369059	-2.923465	2.257966
C	1.008251	0.373215	3.676275
C	1.513344	0.423186	5.123579
C	0.328640	1.688487	3.294282
C	0.037288	-3.979416	1.238906

C	-0.412509	-5.395040	1.608983
C	-0.442265	-3.625148	-0.168169
C	-4.080435	-0.072216	-3.776552
C	-3.338503	0.507961	-2.603467
C	-3.617083	1.833207	-2.261482
C	-3.285740	2.494654	-1.072184
C	-3.962410	3.815875	-0.819773
C	-2.576982	-1.700788	-2.150804
C	-1.714856	-2.341700	-3.052853
C	-1.896987	-3.707653	-3.280812
C	-2.907634	-4.417463	-2.651015
C	-3.745810	-3.767412	-1.755472
C	-3.593843	-2.408287	-1.483052
C	-0.613202	-1.613469	-3.801466
C	-0.738890	-1.795048	-5.320580
C	0.767865	-2.074937	-3.317788
C	-4.480389	-1.762368	-0.431145
C	-5.971793	-2.033262	-0.655354
C	-4.025699	-2.239790	0.952596
C	-2.307737	2.725813	1.090000
C	-1.482865	3.860361	1.161672
C	-1.457010	4.584288	2.354623
C	-2.239134	4.210500	3.435811
C	-3.032423	3.073700	3.352407
C	-3.074314	2.298781	2.192393
C	-0.590196	4.288447	0.010465
C	-0.564855	5.804206	-0.216895
C	0.827127	3.754605	0.235820
C	-3.941114	1.046821	2.139232
C	-4.254111	0.478898	3.526968
C	-5.258087	1.277972	1.382038
H	4.251029	-2.505116	0.180734
H	3.453593	-0.309119	-3.532769
H	5.163566	-0.606420	-3.848502
H	4.682854	0.827016	-2.948662
H	6.101182	0.242181	-0.995722
H	6.742300	-1.114670	-1.925801
H	6.118811	-1.384307	-0.291324
H	4.739510	-3.274572	-1.528824
H	5.223059	-2.703011	-3.120271
H	3.504921	-2.785561	-2.695663
H	5.091589	-1.489160	2.289386
H	4.951720	-2.266008	3.878883
H	3.914448	-0.889331	3.471892
H	3.329143	-4.689013	1.445132
H	4.398613	-4.628062	2.850522
H	4.883291	-3.840298	1.360937
H	1.958327	-2.396832	4.222300
H	3.131740	-3.684265	4.459526
H	1.714736	-3.968193	3.453489
H	5.142171	3.938292	-0.513001
H	4.153098	4.945084	-2.531928

H	2.326664	3.779754	-3.710177
H	4.489772	0.560668	0.752871
H	6.601112	1.936094	0.278700
H	6.259945	1.570541	1.976003
H	5.934959	3.180849	1.344883
H	3.487977	3.177704	1.952188
H	3.811946	1.671141	2.835325
H	2.496050	1.748920	1.650313
H	1.101594	0.389144	-2.801480
H	1.543679	2.301820	-5.153149
H	0.640795	0.791496	-5.190905
H	2.393220	0.773797	-4.925798
H	-0.316134	2.250942	-1.883270
H	-0.944781	1.642386	-3.438396
H	-0.061440	3.171992	-3.350365
H	-1.244777	-0.331268	5.022031
H	-2.657994	-2.336429	4.712646
H	-2.057643	-4.014027	3.014554
H	1.884032	0.246961	3.036043
H	0.697904	0.605999	5.830737
H	2.236950	1.236942	5.237633
H	2.005098	-0.509641	5.414362
H	-0.004411	1.670841	2.252547
H	1.008403	2.537205	3.417388
H	-0.556847	1.869761	3.910349
H	1.131300	-3.997781	1.209233
H	-0.115203	-5.663120	2.627495
H	0.039569	-6.116642	0.922187
H	-1.498822	-5.510699	1.528753
H	-1.533258	-3.651280	-0.222987
H	-0.048297	-4.330966	-0.905207
H	-0.115922	-2.625528	-0.464581
H	-3.404397	-0.617062	-4.438940
H	-4.583546	0.712246	-4.341455
H	-4.832539	-0.789050	-3.433604
H	-4.283822	2.364074	-2.927239
H	-3.233600	4.630238	-0.870334
H	-4.405566	3.849172	0.177803
H	-4.739301	3.998502	-1.561181
H	-1.238194	-4.224123	-3.972093
H	-3.038176	-5.475783	-2.851579
H	-4.524021	-4.330361	-1.249858
H	-0.710736	-0.546571	-3.581843
H	-0.605353	-2.841177	-5.611694
H	0.028842	-1.214265	-5.839304
H	-1.716844	-1.471547	-5.689033
H	0.925200	-1.829674	-2.263176
H	1.560429	-1.590243	-3.896236
H	0.883600	-3.157784	-3.427871
H	-4.341670	-0.678110	-0.468276
H	-6.217335	-3.092974	-0.537113
H	-6.290573	-1.724928	-1.655614

H	-6.568680	-1.477923	0.074281
H	-4.638741	-1.795517	1.740773
H	-2.980060	-1.976256	1.143916
H	-4.106873	-3.328546	1.038045
H	-0.822502	5.460746	2.433661
H	-2.222389	4.795274	4.349748
H	-3.624371	2.785355	4.212805
H	-0.975342	3.837685	-0.907119
H	-0.042350	6.330228	0.587856
H	-1.573231	6.223425	-0.289977
H	-0.032587	6.028095	-1.146117
H	1.493804	4.035899	-0.582659
H	0.829105	2.662204	0.304821
H	1.241608	4.140517	1.172408
H	-3.370737	0.278219	1.601579
H	-4.704144	-0.512535	3.431660
H	-3.353760	0.377048	4.135787
H	-4.973012	1.105511	4.064892
H	-5.877613	0.376781	1.426022
H	-5.823253	2.094631	1.843525
H	-5.107821	1.518181	0.327619
	12		
PhF			
F	0.000000	0.000000	2.277650
C	0.000000	0.000000	0.926293
C	0.000000	1.216537	0.261008
C	0.000000	1.206822	-1.132789
C	0.000000	0.000000	-1.831527
C	0.000000	-1.216537	0.261008
C	0.000000	-1.206822	-1.132789
H	0.000000	2.141115	0.827432
H	0.000000	2.148693	-1.672198
H	0.000000	0.000000	-2.916549
H	0.000000	-2.141115	0.827432
H	0.000000	-2.148693	-1.672198

	55		
L*Zn+			
Zn	-0.000009	-1.213573	0.000068
N	-1.581653	-0.280996	0.025541
N	1.581641	-0.280996	-0.025539
C	-1.322371	1.047521	0.041467
C	0.000000	1.560788	-0.000048
C	1.322383	1.047516	-0.041490
C	-2.472805	2.093779	0.050334
C	-3.024980	2.209414	-1.387632
C	-3.610432	1.716417	1.023970
C	-1.984234	3.496255	0.472285
C	2.472825	2.093758	-0.050333
C	1.984312	3.496236	-0.472352
C	3.610556	1.716387	-1.023838
C	3.024862	2.209420	1.387695

C	-2.841291	-0.972153	0.007360
C	-3.340454	-1.508752	1.194828
C	-4.516124	-2.251979	1.176419
C	-5.184033	-2.477722	-0.026561
C	-4.661872	-1.971609	-1.214008
C	-3.482466	-1.229320	-1.202688
C	2.841285	-0.972162	-0.007387
C	3.482437	-1.229425	1.202658
C	4.661832	-1.971723	1.213936
C	5.184010	-2.477741	0.026451
C	4.516132	-2.251893	-1.176521
C	3.340458	-1.508661	-1.194890
H	-0.000007	2.629006	-0.000071
H	-2.228669	2.449950	-2.098959
H	-3.762154	3.017182	-1.420328
H	-3.524503	1.295662	-1.711072
H	-4.232162	0.899608	0.662792
H	-4.263239	2.584251	1.146397
H	-3.224136	1.452110	2.013319
H	-1.492247	3.486310	1.449712
H	-2.855085	4.151555	0.547744
H	-1.316385	3.956628	-0.261017
H	1.492426	3.486299	-1.449831
H	2.855175	4.151528	-0.547727
H	1.316393	3.956611	0.260885
H	4.232300	0.899627	-0.662575
H	4.263334	2.584248	-1.146229
H	3.224364	1.452036	-2.013213
H	2.228497	2.450070	2.098924
H	3.762110	3.017118	1.420430
H	3.524261	1.295636	1.711247
H	-4.912012	-2.652842	2.103446
H	-6.101833	-3.055321	-0.038699
H	-5.166577	-2.159931	-2.155640
H	5.166520	-2.160142	2.155558
H	6.101802	-3.055353	0.038560
H	4.912038	-2.652676	-2.103574
H	-3.060847	-0.851037	-2.128614
H	-2.825872	-1.311045	2.130684
H	2.825883	-1.310888	-2.130734
H	3.060807	-0.851191	2.128600

37

LAl(I)

Al	-0.000002	-1.170230	-0.001210
N	-1.395176	0.270087	-0.000693
N	1.395176	0.270086	-0.000570
C	-2.474190	2.485592	-0.000480
C	-1.251462	1.598006	-0.000727
C	0.000000	2.225907	-0.000909
C	1.251462	1.598005	-0.000686
C	2.474191	2.485591	-0.000538

C	-2.724882	-0.263659	-0.000096
C	-3.362096	-0.559406	-1.206012
C	-4.638191	-1.116678	-1.203756
C	-5.281601	-1.391853	0.001128
C	-4.638234	-1.113970	1.205420
C	-3.362140	-0.556705	1.206457
C	2.724883	-0.263659	0.000000
C	3.362340	-0.558810	-1.205932
C	4.638435	-1.116080	-1.203696
C	5.281602	-1.391852	0.001183
C	4.637991	-1.114566	1.205481
C	3.361896	-0.557301	1.206538
H	-3.094232	2.287307	0.878474
H	-3.096468	2.284979	-0.877300
H	-2.191502	3.538780	-0.002162
H	0.000001	3.308305	-0.001101
H	3.095904	2.285563	-0.877899
H	3.094798	2.286717	0.877877
H	2.191502	3.538780	-0.001374
H	-5.126370	-1.344049	-2.146285
H	-6.274046	-1.830861	0.001603
H	-5.126442	-1.339233	2.148440
H	5.126804	-1.342988	-2.146238
H	6.274047	-1.830860	0.001641
H	5.126010	-1.340293	2.148488
H	-2.847962	-0.348061	2.139869
H	-2.847911	-0.352803	-2.139870
H	2.847535	-0.349103	2.139947
H	2.848338	-0.351763	-2.139793

	67		
C1			
Zn	-0.000213	-0.265165	0.851113
N	1.535194	0.683646	0.389153
N	-1.529042	0.692871	0.386722
C	1.315220	1.963625	0.032640
C	0.008758	2.500938	-0.046680
C	-1.300894	1.971275	0.029156
C	2.481567	2.932172	-0.311054
C	3.771826	2.620038	0.474413
C	2.760794	2.853360	-1.827064
C	2.111053	4.395477	0.025420
C	-2.461681	2.944788	-0.319171
C	-2.751597	2.849901	-1.832365
C	-2.079534	4.409587	-0.004028
C	-3.748410	2.650394	0.478959
C	2.723625	-0.098507	0.242203
C	3.112825	-0.554849	-1.017818
C	4.200595	-1.416366	-1.139580
C	4.883922	-1.850713	-0.005681
C	4.470189	-1.422043	1.255717
C	3.389569	-0.553972	1.381742

C	-2.723011	-0.081555	0.242954
C	-3.114529	-0.542396	-1.014677
C	-4.208251	-1.396976	-1.132386
C	-4.895203	-1.820354	0.003412
C	-4.479083	-1.387793	1.262720
C	-3.392654	-0.526455	1.384587
H	0.012361	3.547006	-0.273093
H	3.576831	2.518319	1.546621
H	4.469174	3.451317	0.342881
H	4.281645	1.722607	0.127838
H	3.161280	1.880781	-2.119009
H	3.504323	3.610587	-2.094436
H	1.853431	3.049382	-2.406683
H	1.357552	4.813736	-0.646692
H	3.004246	5.013850	-0.092137
H	1.761821	4.502830	1.056634
H	-1.845920	3.029051	-2.420080
H	-3.488579	3.611845	-2.104404
H	-3.164625	1.878366	-2.109552
H	-1.725822	4.528931	1.024352
H	-2.968846	5.032516	-0.126845
H	-1.325747	4.812873	-0.685012
H	-4.266041	1.752009	0.146677
H	-4.441565	3.484403	0.342618
H	-3.546215	2.559994	1.550876
H	4.506447	-1.756004	-2.123859
H	5.730665	-2.521721	-0.102116
H	4.993345	-1.759437	2.144371
H	-4.515771	-1.739978	-2.114978
H	-5.746532	-2.485990	-0.089845
H	-5.004907	-1.716849	2.152920
F	-0.005391	-2.148224	1.615128
C	-0.010215	-3.016877	0.485399
C	-1.241886	-3.377500	-0.017544
C	-1.228530	-4.190885	-1.150425
C	-0.019393	-4.597177	-1.713833
C	1.217265	-3.384352	-0.022896
C	1.194446	-4.197619	-1.155713
H	-2.164082	-3.042155	0.446058
H	-2.170422	-4.505357	-1.585910
H	-0.023071	-5.232361	-2.592658
H	2.143326	-3.054410	0.436874
H	2.132655	-4.517391	-1.595263
H	3.073057	-0.204217	2.360127
H	2.554139	-0.245814	-1.895211
H	-3.074232	-0.173574	2.361219
H	-2.552784	-0.242808	-1.893377

	67		
C2			
Zn	0.028380	-0.586869	0.258504
N	-1.139637	0.878022	0.076594

N	1.792433	0.091098	0.114520
C	-0.582913	2.096179	-0.070872
C	0.816309	2.278065	-0.081885
C	1.936350	1.420487	-0.007800
C	-1.445715	3.389913	-0.169401
C	-2.657728	3.239245	-1.114784
C	-1.930501	3.752578	1.251306
C	-0.637340	4.595536	-0.696875
C	3.333968	2.095188	-0.116942
C	4.375510	1.466273	0.832908
C	3.281197	3.599035	0.231732
C	3.818812	1.977258	-1.578136
C	-2.532954	0.553707	0.126948
C	-3.211623	0.543223	1.345428
C	-4.542139	0.130984	1.400128
C	-5.192931	-0.296221	0.244798
C	-4.501496	-0.322718	-0.966182
C	-3.174168	0.089217	-1.023320
C	2.803447	-0.917285	0.081956
C	3.199698	-1.533570	1.272085
C	4.078389	-2.612430	1.243487
C	4.556912	-3.096419	0.026330
C	4.142685	-2.499390	-1.162196
C	3.258018	-1.422480	-1.137747
H	1.095124	3.304842	-0.178154
H	-2.368073	2.801773	-2.075377
H	-3.068292	4.232223	-1.315613
H	-3.463179	2.643447	-0.689779
H	-2.632900	3.018110	1.646998
H	-2.446785	4.716962	1.218382
H	-1.087186	3.843973	1.943118
H	0.139179	4.927340	-0.002311
H	-1.321105	5.438946	-0.820218
H	-0.182015	4.392709	-1.671117
H	3.989053	1.379074	1.853392
H	5.254171	2.115616	0.867323
H	4.719255	0.486338	0.506411
H	2.734961	4.190917	-0.507617
H	4.303096	3.986078	0.242264
H	2.847508	3.777245	1.220492
H	4.027303	0.944118	-1.859602
H	4.747732	2.544067	-1.694583
H	3.080745	2.390761	-2.272563
H	-5.069174	0.145465	2.348824
H	-6.230519	-0.609898	0.286984
H	-4.996175	-0.665681	-1.868755
H	4.394892	-3.071942	2.174213
H	5.244380	-3.934919	0.005123
H	4.501891	-2.875648	-2.114567
F	-3.244286	-3.412708	-1.600958
C	-2.266431	-3.158899	-0.734460
C	-0.954248	-3.196084	-1.188245

C	0.070831	-2.951506	-0.272005
C	-0.237385	-2.632564	1.071450
C	-2.604263	-2.859589	0.582553
C	-1.586090	-2.591148	1.484062
H	-0.744863	-3.440526	-2.223010
H	1.109957	-3.048178	-0.576703
H	0.566283	-2.572412	1.802718
H	-3.647567	-2.816680	0.871875
H	-1.828557	-2.359703	2.515433
H	-2.633939	0.081804	-1.965556
H	-2.696252	0.860829	2.246684
H	2.910889	-0.973646	-2.063255
H	2.842538	-1.136296	2.218305

	104		
C3			
Al	-3.260740	-1.419563	0.149389
Zn	1.517477	0.036948	-0.714162
N	3.306583	0.135800	-0.077819
N	0.839129	1.808378	-0.462092
N	-2.740870	-1.282428	2.054294
N	-5.074218	-0.816236	0.622728
C	3.619684	1.163907	0.728810
C	2.714243	2.218647	0.977849
C	1.483563	2.605514	0.397104
C	4.954915	1.190775	1.532600
C	4.865515	0.083709	2.605524
C	6.207876	0.971445	0.655316
C	5.180132	2.524575	2.274041
C	0.864551	3.949050	0.876514
C	0.476458	4.881209	-0.292148
C	1.812862	4.758144	1.782957
C	-0.386144	3.605754	1.714239
C	4.208971	-0.843553	-0.598524
C	4.893885	-0.571573	-1.786133
C	5.717662	-1.535489	-2.356341
C	5.848214	-2.789686	-1.758761
C	5.133029	-3.075739	-0.598965
C	4.305398	-2.110959	-0.027573
C	-0.234511	2.149589	-1.340769
C	0.064426	2.681196	-2.600277
C	-0.948582	2.894796	-3.528737
C	-2.265740	2.550207	-3.223097
C	-2.559669	1.989756	-1.984024
C	-1.547994	1.789023	-1.046527
C	-2.910617	-1.085931	4.507008
C	-3.519655	-1.072770	3.126786
C	-4.891059	-0.835651	3.019212
C	-5.624110	-0.677678	1.833753
C	-7.086650	-0.329713	1.962302
C	-1.336920	-1.456995	2.253132
C	-0.797178	-2.728702	2.461040

C	0.578354	-2.892087	2.616931
C	1.425640	-1.787781	2.559039
C	0.893231	-0.518849	2.333150
C	-0.481107	-0.355794	2.170250
C	-5.865936	-0.494885	-0.530324
C	-6.358431	-1.510465	-1.349335
C	-7.076083	-1.189996	-2.500014
C	-7.300813	0.141538	-2.840601
C	-6.801483	1.156840	-2.026145
C	-6.081305	0.840709	-0.877789
H	3.073197	2.916605	1.702629
H	3.987334	0.226255	3.244704
H	5.753954	0.123800	3.243227
H	4.822154	-0.912380	2.163271
H	6.329284	-0.059173	0.327347
H	7.093224	1.227570	1.243882
H	6.198544	1.619253	-0.226589
H	5.210216	3.377856	1.589670
H	6.146569	2.475862	2.782246
H	4.430671	2.714947	3.047391
H	1.302372	4.998269	-1.000722
H	0.244475	5.870551	0.112360
H	-0.402158	4.541647	-0.837830
H	2.052248	4.240469	2.716353
H	1.312990	5.690137	2.059610
H	2.744670	5.023375	1.274253
H	-1.159519	3.120247	1.117158
H	-0.813037	4.526321	2.124115
H	-0.125231	2.953489	2.555040
H	6.263952	-1.305959	-3.265707
H	6.497856	-3.539113	-2.198423
H	5.208415	-4.055387	-0.138604
H	-0.708177	3.324295	-4.496064
H	-3.053966	2.704010	-3.952159
H	-3.574737	1.683796	-1.750311
H	-2.439869	-2.051357	4.714319
H	-3.666023	-0.895539	5.269129
H	-2.129071	-0.324923	4.591229
H	-5.437473	-0.714070	3.945388
H	-7.692988	-0.936018	1.284695
H	-7.259240	0.717058	1.695510
H	-7.431061	-0.483104	2.985241
H	0.985084	-3.883693	2.787142
H	2.493621	-1.907066	2.709265
H	1.541663	0.353180	2.310234
H	-7.459616	-1.985309	-3.130740
H	-7.861508	0.387673	-3.736145
H	-6.971982	2.197172	-2.285147
F	1.885020	-4.306191	-0.795497
C	1.555242	-3.202982	-1.475802
C	0.480299	-2.445562	-1.035111
C	0.128773	-1.296221	-1.766181

C	0.897413	-0.906549	-2.884994
C	2.300159	-2.859809	-2.597956
C	1.978696	-1.698199	-3.290544
H	-0.114767	-2.756609	-0.182191
H	-0.810206	-0.795335	-1.522330
H	0.601844	-0.030866	-3.455659
H	3.138260	-3.480084	-2.893296
H	2.567259	-1.411731	-4.154682
H	-0.900547	0.632338	2.006126
H	-1.463983	-3.584569	2.505730
H	-5.685776	1.626174	-0.239080
H	-6.180654	-2.546938	-1.079420
H	3.723654	-2.345590	0.857090
H	4.794111	0.410333	-2.239160
H	-1.774580	1.326077	-0.091187
H	1.093050	2.942430	-2.831728

	104		
TS1			
Al	3.503175	-0.014171	-0.745577
Zn	-1.785167	0.049907	-0.099044
N	-3.226119	-1.164951	0.209995
N	-2.625373	1.758849	-0.248146
N	4.891799	1.005585	0.145155
N	4.455437	-1.689344	-0.431182
C	-4.474515	-0.679438	0.290107
C	-4.743967	0.700750	0.162081
C	-3.956300	1.841901	-0.101224
C	-5.697858	-1.601321	0.577394
C	-5.708676	-2.865752	-0.309695
C	-5.664693	-2.003198	2.067558
C	-7.046193	-0.892154	0.329752
C	-4.704520	3.201501	-0.223200
C	-4.198469	4.190223	0.848853
C	-6.226535	3.070582	-0.014201
C	-4.504633	3.791597	-1.635808
C	-2.808840	-2.529519	0.273818
C	-2.460266	-3.110353	1.492681
C	-1.913889	-4.391642	1.527581
C	-1.688724	-5.091930	0.345231
C	-2.008912	-4.500498	-0.876692
C	-2.557858	-3.223505	-0.913462
C	-1.691679	2.794972	-0.562275
C	-1.008403	3.456785	0.459880
C	0.057859	4.300326	0.160489
C	0.458348	4.484774	-1.160952
C	-0.220541	3.825134	-2.183995
C	-1.284223	2.977432	-1.886438
C	7.104709	1.569689	1.066052
C	6.118456	0.586961	0.488935
C	6.528934	-0.741505	0.330750
C	5.734394	-1.828706	-0.056009

C	6.371410	-3.196410	-0.039752
C	4.510404	2.361201	0.420870
C	4.546563	3.320664	-0.591277
C	4.132574	4.624928	-0.329289
C	3.672040	4.975063	0.937739
C	3.620757	4.012443	1.944814
C	4.033331	2.707861	1.686702
C	3.643142	-2.852485	-0.628724
C	3.128225	-3.135957	-1.894214
C	2.252051	-4.205935	-2.068657
C	1.886340	-4.998336	-0.983889
C	2.396521	-4.712804	0.282270
C	3.263467	-3.639693	0.461287
H	-5.781625	0.929096	0.266956
H	-5.547832	-2.617176	-1.363578
H	-6.689369	-3.342632	-0.228271
H	-4.968603	-3.605987	-0.011466
H	-4.816273	-2.646440	2.302046
H	-6.575945	-2.558608	2.310901
H	-5.625611	-1.118628	2.711343
H	-7.229153	-0.067929	1.024676
H	-7.848889	-1.617000	0.488189
H	-7.134568	-0.519666	-0.695408
H	-4.233527	3.740008	1.846532
H	-4.847766	5.070754	0.857033
H	-3.184746	4.537418	0.655842
H	-6.699059	2.425310	-0.760693
H	-6.671790	4.063530	-0.119226
H	-6.482170	2.707701	0.986031
H	-3.482539	4.121905	-1.815115
H	-5.154609	4.663918	-1.752849
H	-4.779035	3.064159	-2.406880
H	-1.657699	-4.838819	2.482997
H	-1.263960	-6.089880	0.374827
H	-1.828289	-5.032832	-1.805015
H	0.590407	4.800324	0.963214
H	1.300311	5.129009	-1.389032
H	0.086803	3.955984	-3.216341
H	7.187374	2.455572	0.430453
H	8.089635	1.115663	1.173399
H	6.772322	1.913484	2.050073
H	7.556306	-0.963681	0.588629
H	6.049909	-3.790017	-0.898698
H	6.085486	-3.747361	0.860995
H	7.458398	-3.110001	-0.050192
H	4.177761	5.369578	-1.117760
H	3.359063	5.994193	1.141370
H	3.265811	4.278776	2.935710
H	1.858629	-4.418561	-3.057331
H	1.207857	-5.833486	-1.122537
H	2.111352	-5.321995	1.134458
F	0.153371	-0.738900	-1.999880

C	0.236323	-0.565024	-0.651099
C	0.770651	0.664070	-0.188607
C	0.925540	0.810502	1.213199
C	0.871685	-0.297366	2.035837
C	0.349911	-1.725137	0.152759
C	0.650555	-1.579562	1.493247
H	0.805784	1.519190	-0.850835
H	1.186645	1.786764	1.605121
H	1.043691	-0.186866	3.101475
H	0.168294	-2.700184	-0.287923
H	0.685995	-2.458843	2.125612
H	3.997865	1.950040	2.464539
H	4.908109	3.041795	-1.576363
H	3.638171	-3.393801	1.450543
H	3.413459	-2.516774	-2.738908
H	-2.803874	-2.755356	-1.861707
H	-2.599820	-2.546929	2.409811
H	-1.797713	2.439192	-2.677407
H	-1.304774	3.285478	1.490206

	104		
C4			
Al	3.005257	0.027636	-0.074239
Zn	-1.593759	-0.096464	-0.286162
N	-2.897548	-1.341137	0.414572
N	-2.717650	1.442274	-0.603489
N	4.330541	1.194766	0.570842
N	4.152482	-1.355985	-0.625284
C	-4.199236	-1.036699	0.470961
C	-4.674452	0.226944	0.061321
C	-4.040710	1.393879	-0.420551
C	-5.258526	-2.060615	0.974297
C	-5.201959	-3.346978	0.123551
C	-5.016037	-2.392406	2.462212
C	-6.702956	-1.531342	0.872166
C	-4.961469	2.621119	-0.692701
C	-4.853471	3.592504	0.501838
C	-6.447453	2.223325	-0.824499
C	-4.594659	3.355824	-1.999720
C	-2.236151	-2.535701	0.822523
C	-1.742494	-2.661014	2.124294
C	-0.940300	-3.743959	2.474131
C	-0.598997	-4.703630	1.523397
C	-1.068339	-4.570345	0.217555
C	-1.874510	-3.491723	-0.132922
C	-1.919679	2.551940	-1.002455
C	-1.449087	3.467095	-0.058249
C	-0.547439	4.461645	-0.430799
C	-0.089320	4.540454	-1.743413
C	-0.534843	3.609860	-2.682606
C	-1.434549	2.616349	-2.313184
C	6.609117	2.011439	1.003842

C	5.653247	0.959678	0.509673
C	6.190556	-0.221358	-0.012740
C	5.494865	-1.314388	-0.539141
C	6.299029	-2.487119	-1.032541
C	3.872413	2.467249	1.067314
C	3.494883	3.456295	0.161843
C	2.975446	4.660815	0.630883
C	2.832759	4.875854	1.999135
C	3.215221	3.883615	2.902065
C	3.731520	2.678131	2.437975
C	3.522224	-2.564168	-1.092122
C	2.964221	-2.607759	-2.365620
C	2.279161	-3.748638	-2.779853
C	2.153480	-4.839268	-1.924964
C	2.712906	-4.791078	-0.647695
C	3.393648	-3.653863	-0.228479
H	-5.735242	0.326577	0.139502
H	-5.286995	-3.114571	-0.943174
H	-6.042916	-3.992990	0.393752
H	-4.289274	-3.919586	0.283169
H	-4.114882	-2.983914	2.618363
H	-5.859902	-2.977043	2.841613
H	-4.945531	-1.478224	3.061072
H	-6.873249	-0.654918	1.504777
H	-7.380907	-2.315380	1.220813
H	-6.987705	-1.289444	-0.156188
H	-5.073663	3.080136	1.443878
H	-5.580796	4.401434	0.377387
H	-3.864520	4.047282	0.572319
H	-6.603199	1.469108	-1.601908
H	-7.019727	3.112254	-1.103310
H	-6.875895	1.857862	0.112688
H	-3.676118	3.935251	-1.922949
H	-5.396079	4.057989	-2.246708
H	-4.495099	2.657246	-2.836701
H	-0.578089	-3.833881	3.493812
H	0.024832	-5.548170	1.797635
H	-0.803000	-5.305464	-0.535531
H	-0.196591	5.169954	0.313581
H	0.607747	5.319411	-2.035463
H	-0.180261	3.656829	-3.707420
H	6.411533	2.971096	0.518448
H	7.640460	1.720366	0.808831
H	6.486672	2.168554	2.079215
H	7.269554	-0.295837	-0.015039
H	5.955428	-2.806893	-2.019470
H	6.182496	-3.342886	-0.361295
H	7.357168	-2.233123	-1.085306
H	2.680576	5.428612	-0.076575
H	2.428498	5.814427	2.363506
H	3.106292	4.047425	3.969127
H	1.837246	-3.776798	-3.769916

H	1.618996	-5.725584	-2.250425
H	2.611659	-5.636426	0.024908
F	0.469915	-0.832370	-2.047400
C	0.360705	-0.387591	-0.706968
C	1.228444	0.846179	-0.505926
C	1.019208	1.300957	0.907163
C	1.102779	0.360375	1.879473
C	0.904192	-1.407606	0.204422
C	1.330492	-1.023082	1.451937
H	1.072098	1.634752	-1.239520
H	0.953522	2.358509	1.140015
H	1.080563	0.607913	2.934446
H	0.911380	-2.453782	-0.091161
H	1.643662	-1.774772	2.169170
H	4.011863	1.890934	3.131646
H	3.597493	3.276238	-0.904142
H	3.813212	-3.596742	0.771902
H	3.049316	-1.747470	-3.021349
H	-2.228984	-3.376551	-1.152711
H	-1.987928	-1.897409	2.856024
H	-1.779381	1.885663	-3.038322
H	-1.779204	3.381398	0.972140

104

C5

Al	2.888456	0.075984	-0.063869
Zn	-1.402234	-0.082210	-0.147037
N	-2.497628	-1.406910	0.746733
N	-2.712329	1.204888	-0.739614
N	4.165244	1.035925	0.927466
N	4.098767	-0.995618	-1.026327
C	-3.832895	-1.317824	0.681126
C	-4.469383	-0.263408	-0.005045
C	-4.015037	0.919689	-0.628533
C	-4.759105	-2.381819	1.343584
C	-4.488098	-3.775329	0.737819
C	-4.541340	-2.409627	2.871807
C	-6.258281	-2.094345	1.123974
C	-5.133730	1.878364	-1.143165
C	-5.497305	2.860027	-0.009392
C	-6.417067	1.100025	-1.518530
C	-4.736084	2.672248	-2.405259
C	-1.707844	-2.372976	1.434645
C	-1.273887	-2.123285	2.739775
C	-0.364042	-2.976746	3.357151
C	0.141143	-4.080377	2.671599
C	-0.274369	-4.323322	1.363622
C	-1.187333	-3.472628	0.746875
C	-2.142088	2.454353	-1.117177
C	-2.061472	3.497698	-0.192343
C	-1.404118	4.680063	-0.523812
C	-0.800380	4.821658	-1.771787

C	-0.855291	3.768705	-2.684652
C	-1.514103	2.588792	-2.356983
C	6.399785	1.799139	1.594097
C	5.494356	0.924554	0.769279
C	6.083397	0.022719	-0.125875
C	5.433811	-0.903466	-0.953668
C	6.275346	-1.837885	-1.779433
C	3.631906	1.988770	1.862774
C	3.388022	3.300069	1.456414
C	2.785179	4.196331	2.334867
C	2.420035	3.783355	3.615061
C	2.665226	2.471782	4.017527
C	3.269008	1.572410	3.142345
C	3.485286	-2.027740	-1.817893
C	3.162232	-1.791969	-3.152660
C	2.474263	-2.760455	-3.879431
C	2.105557	-3.960005	-3.274227
C	2.431472	-4.193013	-1.938727
C	3.118493	-3.227947	-1.208185
H	-5.533622	-0.353589	-0.020633
H	-4.550363	-3.745723	-0.355080
H	-5.247619	-4.476305	1.097290
H	-3.516567	-4.176758	1.022244
H	-3.578222	-2.832169	3.153539
H	-5.316143	-3.030517	3.331750
H	-4.622525	-1.404023	3.297891
H	-6.570754	-1.143577	1.566398
H	-6.833073	-2.883437	1.616444
H	-6.537381	-2.105636	0.066059
H	-5.774761	2.321722	0.902253
H	-6.352411	3.469929	-0.318699
H	-4.673214	3.538146	0.220963
H	-6.209772	0.287739	-2.221723
H	-7.114203	1.791188	-1.999257
H	-6.940889	0.687951	-0.652445
H	-4.031726	3.477624	-2.204650
H	-5.634566	3.133798	-2.824011
H	-4.309350	2.020596	-3.174070
H	-0.047856	-2.777082	4.376191
H	0.849554	-4.746012	3.153395
H	0.111189	-5.179770	0.819382
H	-1.360213	5.489663	0.198087
H	-0.292454	5.744477	-2.031726
H	-0.387142	3.867559	-3.659072
H	6.228955	2.854489	1.362619
H	7.446696	1.564418	1.405918
H	6.192288	1.670566	2.660161
H	7.164516	0.020003	-0.160366
H	6.053622	-1.715924	-2.843471
H	6.051402	-2.878203	-1.525924
H	7.336988	-1.656174	-1.616693
H	2.598809	5.217265	2.017602

H	1.950107	4.482696	4.298453
H	2.387330	2.147588	5.015201
H	2.220184	-2.572798	-4.917306
H	1.568365	-4.712202	-3.842249
H	2.149951	-5.127340	-1.463813
F	0.538112	0.108848	-2.913229
C	0.766229	0.106374	-1.597320
C	1.380269	1.274164	-0.976859
C	0.729411	1.545411	0.339709
C	0.576670	0.463316	1.151041
C	0.532555	-0.988987	-0.803680
C	1.172523	-0.800882	0.570314
H	1.514948	2.130165	-1.630901
H	0.473585	2.560623	0.621003
H	0.230810	0.533618	2.178132
H	0.302242	-1.965201	-1.219327
H	1.088782	-1.679370	1.205066
H	3.456726	0.545420	3.442160
H	3.662613	3.606906	0.451264
H	3.360783	-3.390802	-0.162173
H	3.437065	-0.846619	-3.611036
H	-1.506963	-3.654224	-0.274999
H	-1.661936	-1.256052	3.265680
H	-1.566037	1.770042	-3.067448
H	-2.505575	3.370321	0.789965

104

TS2

Al	-2.974364	-0.070887	-0.002330
Zn	1.524617	0.125543	-0.248028
N	2.779622	1.387188	0.501233
N	2.678169	-1.373177	-0.635996
N	-4.224601	-1.268932	0.733598
N	-4.210859	1.214628	-0.605040
C	4.082699	1.090398	0.593895
C	4.587588	-0.146536	0.144435
C	3.996723	-1.294118	-0.430843
C	5.109802	2.093605	1.196803
C	5.116376	3.411024	0.392479
C	4.768048	2.371329	2.676190
C	6.556366	1.560512	1.173834
C	4.968438	-2.464286	-0.774597
C	4.949349	-3.477311	0.389455
C	6.426130	-1.978401	-0.938565
C	4.605686	-3.178460	-2.094204
C	2.111791	2.595808	0.855486
C	1.518808	2.736402	2.112672
C	0.713295	3.836958	2.393930
C	0.469897	4.799539	1.416593
C	1.041860	4.653142	0.153495
C	1.850879	3.556628	-0.127712
C	1.932008	-2.491944	-1.104606

C	1.531983	-3.499242	-0.224463
C	0.693717	-4.522243	-0.661535
C	0.228529	-4.538024	-1.974194
C	0.601835	-3.515582	-2.846820
C	1.438635	-2.493345	-2.413012
C	-6.444249	-2.167845	1.281070
C	-5.557266	-1.096679	0.706069
C	-6.168086	0.032977	0.148837
C	-5.543290	1.125559	-0.463713
C	-6.413197	2.236598	-0.987219
C	-3.683489	-2.497967	1.252772
C	-3.307246	-3.503564	0.364525
C	-2.699831	-4.660857	0.846089
C	-2.466924	-4.812759	2.210609
C	-2.847384	-3.804728	3.096462
C	-3.451907	-2.645837	2.619301
C	-3.626757	2.405214	-1.159931
C	-3.074934	2.371736	-2.437725
C	-2.404656	3.489755	-2.931277
C	-2.286689	4.636126	-2.151457
C	-2.841661	4.667172	-0.871418
C	-3.507065	3.553032	-0.372676
H	5.645932	-0.237006	0.258222
H	5.257305	3.217107	-0.676002
H	5.949856	4.033391	0.732177
H	4.204183	3.991342	0.523654
H	3.854091	2.951920	2.793463
H	5.580307	2.947055	3.130536
H	4.665755	1.436030	3.236587
H	6.677808	0.654069	1.774597
H	7.209335	2.323184	1.607091
H	6.914822	1.366596	0.158381
H	5.177247	-2.985692	1.340577
H	5.709418	-4.245503	0.213340
H	3.985571	-3.981141	0.477044
H	6.508876	-1.174253	-1.675977
H	7.033389	-2.817449	-1.288422
H	6.874430	-1.642150	0.000085
H	3.725123	-3.813390	-2.012929
H	5.439105	-3.824666	-2.383711
H	4.443100	-2.462675	-2.906247
H	0.271544	3.938535	3.380612
H	-0.156974	5.657201	1.637629
H	0.855382	5.392282	-0.619175
H	0.400710	-5.304572	0.031754
H	-0.418970	-5.338770	-2.316673
H	0.240162	-3.513402	-3.870193
H	-6.231731	-3.134050	0.814962
H	-7.495331	-1.925144	1.130132
H	-6.261042	-2.285600	2.352839
H	-7.248795	0.059560	0.186016
H	-6.115454	2.518461	-2.000402

H	-6.311675	3.129682	-0.363634
H	-7.461022	1.937997	-0.990811
H	-2.407864	-5.441121	0.151066
H	-1.994370	-5.714612	2.585492
H	-2.669146	-3.919752	4.160630
H	-1.966719	3.455750	-3.922901
H	-1.762219	5.504036	-2.537087
H	-2.746991	5.556724	-0.257339
F	-0.372755	0.801128	-2.241676
C	-0.421366	0.425685	-0.899035
C	-1.230900	-0.835395	-0.642952
C	-0.838660	-1.251841	0.756337
C	-0.979154	-0.290503	1.699847
C	-0.878577	1.453745	-0.013848
C	-1.375061	1.052813	1.230085
H	-1.082345	-1.618359	-1.382983
H	-0.619115	-2.285621	1.000007
H	-0.855597	-0.476852	2.760991
H	-0.856864	2.501901	-0.303782
H	-1.632959	1.816072	1.956066
H	-3.733814	-1.846726	3.298681
H	-3.479931	-3.370959	-0.699354
H	-3.917930	3.557284	0.632751
H	-3.153342	1.468320	-3.034133
H	2.286110	3.430764	-1.114435
H	1.688023	1.970469	2.863435
H	1.728046	-1.692533	-3.086581
H	1.868722	-3.466309	0.806808

66

C6 L*ZnPh

Zn	-0.015726	-0.678900	-0.010632
N	-1.468807	0.647324	0.001982
N	1.476641	0.604131	-0.046012
C	-1.267788	1.965957	0.016211
C	0.032262	2.511724	-0.012432
C	1.316721	1.926253	-0.047818
C	-2.449286	2.985668	0.053748
C	-3.275552	2.887293	-1.245902
C	-3.351812	2.733718	1.279842
C	-1.978388	4.450804	0.160854
C	2.522973	2.918449	-0.032715
C	2.110090	4.357267	-0.412652
C	3.639171	2.521696	-1.022711
C	3.093577	2.974376	1.399799
C	-2.713837	-0.045007	-0.009660
C	-3.252070	-0.539300	1.182594
C	-4.384588	-1.346787	1.165216
C	-4.986225	-1.689434	-0.044495
C	-4.435845	-1.225938	-1.237472
C	-3.301719	-0.418582	-1.221575
C	2.705150	-0.114530	-0.040366

C	3.330214	-0.447427	1.163385
C	4.462946	-1.258397	1.167823
C	4.969270	-1.766177	-0.025963
C	4.323599	-1.468374	-1.225910
C	3.194560	-0.658042	-1.232583
H	0.050772	3.579239	-0.005369
H	-2.629426	2.980230	-2.125324
H	-4.003684	3.705034	-1.273942
H	-3.828920	1.951971	-1.318256
H	-3.923150	1.810553	1.200463
H	-4.068285	3.556424	1.374016
H	-2.758909	2.697149	2.200000
H	-1.394529	4.634257	1.068188
H	-2.863362	5.092683	0.206160
H	-1.394049	4.769054	-0.707773
H	1.620211	4.398253	-1.390536
H	3.012360	4.973768	-0.464224
H	1.454592	4.822515	0.328730
H	4.218796	1.661789	-0.692213
H	4.334951	3.360376	-1.125292
H	3.232551	2.301572	-2.015030
H	2.309678	3.227173	2.121180
H	3.868902	3.746666	1.453426
H	3.547761	2.027808	1.695016
H	-4.788736	-1.721169	2.100479
H	-5.864634	-2.326300	-0.057912
H	-4.879738	-1.505641	-2.187822
H	4.938823	-1.507106	2.111310
H	5.846904	-2.404210	-0.020617
H	4.693584	-1.879019	-2.160076
C	-0.035385	-2.628356	0.016829
C	-1.233386	-3.357359	-0.095756
C	-1.253895	-4.752109	-0.073511
C	-0.065723	-5.466076	0.065473
C	1.146874	-3.378477	0.155197
C	1.137477	-4.773309	0.180556
H	-2.180122	-2.833849	-0.202801
H	-2.199517	-5.280204	-0.164004
H	-0.077311	-6.552306	0.083996
H	2.104852	-2.872195	0.244831
H	2.071572	-5.318131	0.289610
H	-2.852419	-0.080758	-2.150345
H	-2.767965	-0.287493	2.121078
H	2.680229	-0.432895	-2.161657
H	2.908858	-0.085548	2.095878

38

C6 LAl-F+

Al	-0.000001	-0.691424	0.000026
N	1.428568	0.417873	0.000010
N	-1.428567	0.417876	0.000010
C	2.483882	2.631906	0.000009

C	1.262265	1.762188	0.000012
C	0.000002	2.369804	0.000016
C	-1.262263	1.762190	0.000012
C	-2.483879	2.631909	0.000010
C	2.758713	-0.159517	-0.000003
C	3.377990	-0.454921	1.212157
C	4.641259	-1.040585	1.207402
C	5.272462	-1.332105	-0.000031
C	4.641242	-1.040564	-1.207450
C	3.377973	-0.454900	-1.212176
C	-2.758713	-0.159514	-0.000003
C	-3.377987	-0.454923	1.212157
C	-4.641256	-1.040587	1.207403
C	-5.272460	-1.332104	-0.000030
C	-4.641243	-1.040559	-1.207450
C	-3.377974	-0.454894	-1.212176
H	3.101519	2.421445	0.877888
H	2.212648	3.686355	0.000029
H	3.101493	2.421473	-0.877895
H	0.000003	3.451303	0.000019
H	-3.101510	2.421454	0.877894
H	-3.101496	2.421472	-0.877889
H	-2.212643	3.686359	0.000022
H	5.129040	-1.272057	2.148008
H	6.255014	-1.791096	-0.000042
H	5.129010	-1.272020	-2.148067
H	-5.129034	-1.272063	2.148009
H	-6.255012	-1.791096	-0.000041
H	-5.129011	-1.272013	-2.148066
F	-0.000013	-2.329476	0.000057
H	2.878913	-0.223206	-2.148849
H	2.878942	-0.223243	2.148841
H	-2.878914	-0.223197	-2.148849
H	-2.878938	-0.223249	2.148841

	104		
C7			
Al	2.181373	-0.044918	-1.144676
Zn	-0.699772	0.161976	0.149262
N	-2.172271	-0.948908	0.739739
N	-1.622999	1.787257	-0.307559
N	3.988147	-0.469030	-0.878163
N	1.710646	-1.527365	-2.195500
C	-3.406554	-0.633302	0.332806
C	-3.665080	0.534980	-0.421608
C	-2.925967	1.716918	-0.616879
C	-4.655813	-1.496831	0.685005
C	-4.359347	-3.008913	0.755774
C	-5.215614	-1.015651	2.040692
C	-5.779267	-1.327296	-0.364207
C	-3.663931	2.920358	-1.272662
C	-3.700829	4.156288	-0.346624

C	-5.128459	2.600464	-1.630201
C	-2.946346	3.271723	-2.592339
C	-1.846333	-1.874934	1.775777
C	-1.958018	-1.483554	3.110561
C	-1.560769	-2.344444	4.131452
C	-1.027915	-3.594797	3.827541
C	-0.881483	-3.972238	2.492748
C	-1.277663	-3.113638	1.473340
C	-0.879744	2.976066	-0.035365
C	-0.961317	3.540237	1.242983
C	-0.139072	4.604054	1.596012
C	0.791518	5.102413	0.683375
C	0.888623	4.527308	-0.581178
C	0.059382	3.466135	-0.940101
C	6.136776	-1.496459	-1.468481
C	4.651279	-1.335441	-1.645432
C	4.009742	-2.112232	-2.629003
C	2.637542	-2.236965	-2.869113
C	2.213407	-3.204538	-3.945668
C	4.630945	0.239404	0.190578
C	4.822333	1.616868	0.077513
C	5.352115	2.330271	1.150350
C	5.685157	1.674025	2.332853
C	5.485511	0.298659	2.443373
C	4.952564	-0.419065	1.377484
C	0.314845	-1.774219	-2.354288
C	-0.524305	-0.710299	-2.718412
C	-1.898037	-0.915798	-2.843375
C	-2.443791	-2.172885	-2.604631
C	-1.613303	-3.223830	-2.224650
C	-0.242760	-3.025509	-2.083014
H	-4.664337	0.585433	-0.797685
H	-3.775514	-3.344131	-0.107351
H	-5.306516	-3.555096	0.751091
H	-3.830775	-3.298272	1.662237
H	-4.526722	-1.224028	2.861511
H	-6.153367	-1.539857	2.252018
H	-5.422998	0.058754	2.018994
H	-6.240584	-0.337078	-0.344290
H	-6.573941	-2.043368	-0.140143
H	-5.426611	-1.528390	-1.380777
H	-4.057576	3.890806	0.653837
H	-4.397406	4.889218	-0.764855
H	-2.732706	4.645221	-0.250876
H	-5.218542	1.768749	-2.335906
H	-5.563963	3.478793	-2.114283
H	-5.734926	2.384141	-0.745203
H	-1.930844	3.630600	-2.429179
H	-3.498288	4.064471	-3.106625
H	-2.906767	2.405025	-3.261286
H	-1.666914	-2.033954	5.166050
H	-0.726356	-4.268653	4.622678

H	-0.463665	-4.943276	2.245059
H	-0.220923	5.041488	2.586066
H	1.436834	5.930064	0.958604
H	1.618300	4.895526	-1.294946
H	6.622904	-0.524939	-1.350112
H	6.573554	-2.017652	-2.320095
H	6.351304	-2.077260	-0.566308
H	4.655962	-2.719699	-3.249171
H	1.321708	-2.854792	-4.469598
H	1.987396	-4.187997	-3.523633
H	3.021278	-3.333149	-4.666576
H	5.505140	3.400566	1.058727
H	6.099820	2.231519	3.166145
H	5.738246	-0.216087	3.364757
H	-2.537774	-0.089289	-3.131186
H	-3.511795	-2.331694	-2.707032
H	-2.034631	-4.204636	-2.026329
F	1.977174	1.415205	-1.961298
C	1.310765	-0.029095	0.665037
C	1.545100	1.153427	1.422396
C	1.804591	1.104386	2.787232
C	1.841908	-0.126900	3.438234
C	1.380748	-1.260759	1.370987
C	1.632741	-1.312819	2.733746
H	1.536515	2.118944	0.923159
H	1.984642	2.022196	3.336313
H	2.041496	-0.165178	4.504843
H	1.234214	-2.191566	0.829017
H	1.652887	-2.267546	3.247738
H	4.767669	-1.485525	1.464641
H	4.551805	2.120212	-0.845958
H	0.398461	-3.840517	-1.762085
H	-0.091769	0.257810	-2.958161
H	-1.178430	-3.402096	0.432886
H	-2.355567	-0.500029	3.342244
H	0.170006	2.994209	-1.908808
H	-1.679096	3.136716	1.951104

	104		
C8			
Al	-1.141663	-1.026513	-1.034725
Zn	0.411927	0.316068	0.222285
N	1.988781	1.337589	-0.225080
N	1.018800	-0.580679	1.853662
N	-0.449662	-2.492219	-1.985870
N	-2.933650	-1.524102	-1.102947
C	3.133126	1.168909	0.432446
C	3.234908	0.275130	1.527843
C	2.307655	-0.534351	2.213014
C	4.452861	1.882132	0.009736
C	5.090197	1.067210	-1.134873
C	4.235597	3.337819	-0.454448

C	5.477536	1.948413	1.163724
C	2.861463	-1.408536	3.376584
C	4.371291	-1.210982	3.616237
C	2.661949	-2.898222	3.025594
C	2.159840	-1.074464	4.709923
C	1.751110	2.099156	-1.405087
C	1.145211	3.355013	-1.317548
C	0.791594	4.043365	-2.473584
C	1.017322	3.477070	-3.727737
C	1.588162	2.208745	-3.814973
C	1.943387	1.516360	-2.659998
C	-0.064481	-1.244049	2.489189
C	-0.820127	-0.579459	3.462065
C	-1.963329	-1.166264	3.995935
C	-2.396105	-2.413270	3.545221
C	-1.676073	-3.063503	2.546284
C	-0.525503	-2.480667	2.017145
C	-0.467510	-4.731725	-3.013717
C	-1.160522	-3.573154	-2.348931
C	-2.538918	-3.672152	-2.116057
C	-3.384938	-2.708682	-1.550546
C	-4.849348	-3.029838	-1.429262
C	0.951676	-2.384240	-2.297381
C	1.373789	-2.185441	-3.612624
C	2.726241	-2.001217	-3.886481
C	3.658165	-1.982145	-2.849246
C	3.232961	-2.157211	-1.535425
C	1.884224	-2.364377	-1.258553
C	-3.837767	-0.551234	-0.544186
C	-4.681038	0.191998	-1.370367
C	-5.502613	1.166980	-0.814152
C	-5.467158	1.416580	0.556875
C	-4.606893	0.688145	1.372338
C	-3.790375	-0.298883	0.825523
H	4.223285	0.223317	1.931141
H	5.289306	0.039026	-0.816116
H	6.043298	1.525097	-1.419559
H	4.452297	1.039537	-2.020504
H	3.776806	3.410320	-1.439003
H	5.208070	3.834217	-0.517217
H	3.621681	3.899196	0.256614
H	5.055120	2.418642	2.056869
H	6.329346	2.551607	0.838264
H	5.879271	0.969965	1.439961
H	4.973441	-1.486588	2.745074
H	4.677394	-1.864005	4.438290
H	4.616697	-0.185440	3.908077
H	1.612026	-3.189671	3.017630
H	3.166771	-3.518669	3.772532
H	3.100055	-3.130761	2.048177
H	2.181975	0.002733	4.904808
H	2.688649	-1.572858	5.528099

H	1.125078	-1.412308	4.740452
H	0.336161	5.025373	-2.392564
H	0.744443	4.017234	-4.628181
H	1.754864	1.751398	-4.785507
H	-2.521376	-0.644349	4.767112
H	-3.287527	-2.868310	3.963487
H	-2.002783	-4.031334	2.178551
H	-0.259376	-4.503612	-4.063190
H	-1.091755	-5.624397	-2.978596
H	0.491296	-4.941867	-2.533431
H	-3.008968	-4.597266	-2.423134
H	-5.431765	-2.446569	-2.148279
H	-5.219504	-2.772105	-0.433490
H	-5.030138	-4.087472	-1.619014
H	3.050185	-1.854517	-4.911643
H	4.708905	-1.819780	-3.064687
H	3.943120	-2.124579	-0.715751
H	-6.161927	1.743075	-1.455034
H	-6.098270	2.188069	0.984164
H	-4.563961	0.885963	2.438232
F	-0.827252	2.067839	1.375944
C	-1.855110	2.891559	0.994026
C	-2.518643	3.591536	1.984533
C	-3.547537	4.447716	1.596632
C	-3.884242	4.580816	0.250666
C	-2.161196	2.992447	-0.350156
C	-3.193403	3.853626	-0.716276
H	-2.229401	3.474167	3.022624
H	-4.080754	5.016044	2.351431
H	-4.682171	5.253813	-0.044223
H	-1.595391	2.434566	-1.087483
H	-3.452188	3.955315	-1.764942
H	1.561089	-2.499248	-0.231234
H	0.642184	-2.163604	-4.414688
H	-3.124073	-0.876493	1.459735
H	-4.687083	0.007450	-2.440683
H	2.363909	0.517346	-2.720360
H	0.972160	3.791218	-0.338646
H	0.042318	-3.006588	1.254684
H	-0.488212	0.395716	3.802613

	104		
C9			
Al	1.539160	0.181600	-0.810734
Zn	-0.748328	0.266248	-0.030329
N	-1.987256	-1.150767	0.465054
N	-1.987930	1.773909	-0.008605
N	1.941929	-0.366995	-2.564770
N	3.038402	1.298672	-0.579255
C	-3.309741	-0.959750	0.494436
C	-3.869740	0.315626	0.264873
C	-3.313418	1.593064	0.060903

C	-4.325258	-2.107306	0.794158
C	-3.931270	-3.451936	0.144458
C	-4.439753	-2.280251	2.323368
C	-5.740333	-1.779562	0.265552
C	-4.341123	2.761441	-0.077545
C	-5.687245	2.259384	-0.652646
C	-3.872302	3.884151	-1.027280
C	-4.625732	3.349681	1.319895
C	-1.299079	-2.361377	0.763288
C	-1.036448	-2.735385	2.082423
C	-0.325374	-3.901786	2.352542
C	0.166374	-4.682643	1.310050
C	-0.035120	-4.273344	-0.007447
C	-0.753704	-3.115035	-0.280106
C	-1.308603	3.020966	-0.000187
C	-1.173340	3.756378	1.180809
C	-0.458342	4.952214	1.187937
C	0.168700	5.405914	0.029411
C	0.085821	4.644440	-1.136533
C	-0.640341	3.457726	-1.149745
C	3.110296	-0.365288	-4.727446
C	2.950999	0.115222	-3.310122
C	3.859548	1.064155	-2.828486
C	3.922894	1.606735	-1.536447
C	5.031068	2.580739	-1.235711
C	1.049050	-1.351566	-3.112542
C	1.423059	-2.694342	-3.159806
C	0.533747	-3.644658	-3.653815
C	-0.737893	-3.262371	-4.078110
C	-1.117715	-1.923448	-4.010637
C	-0.226125	-0.967347	-3.529387
C	3.219842	1.763204	0.767413
C	4.251808	1.253897	1.557867
C	4.354187	1.634936	2.891595
C	3.417649	2.507288	3.445302
C	2.383310	3.005038	2.656296
C	2.282639	2.636995	1.316350
H	-4.938566	0.319270	0.273676
H	-3.663433	-3.325085	-0.909502
H	-4.789547	-4.128246	0.190540
H	-3.106377	-3.950586	0.650569
H	-3.517098	-2.662294	2.762042
H	-5.235248	-2.998403	2.548090
H	-4.692619	-1.331139	2.806673
H	-6.222794	-0.964708	0.811655
H	-6.373566	-2.660156	0.402290
H	-5.734144	-1.533538	-0.800768
H	-5.549966	1.702622	-1.584514
H	-6.319247	3.125266	-0.867094
H	-6.248395	1.634726	0.046624
H	-3.092725	4.514763	-0.603165
H	-4.723172	4.535888	-1.244399

H	-3.512417	3.481316	-1.979403
H	-4.943144	2.567957	2.017022
H	-5.431318	4.087724	1.246166
H	-3.752458	3.855251	1.735828
H	-0.142194	-4.190562	3.382495
H	0.717454	-5.592780	1.523093
H	0.358573	-4.861866	-0.830522
H	-0.385445	5.527349	2.105962
H	0.725378	6.336816	0.036864
H	0.579300	4.981782	-2.042628
H	3.421454	-1.413935	-4.743402
H	3.858432	0.224895	-5.255603
H	2.160912	-0.306499	-5.266544
H	4.611881	1.405453	-3.527183
H	5.834151	2.090665	-0.677537
H	4.668208	3.408692	-0.622081
H	5.453944	2.974731	-2.159859
H	0.831691	-4.687181	-3.700237
H	-1.431278	-4.006063	-4.455974
H	-2.107644	-1.619959	-4.334780
H	5.156439	1.236314	3.503617
H	3.495909	2.797570	4.487749
H	1.651465	3.688399	3.074568
F	2.510604	-1.431529	0.197315
C	2.547491	-1.865317	1.532055
C	2.019214	-1.053440	2.512883
C	2.102378	-1.514425	3.825563
C	2.700064	-2.740401	4.108617
C	3.147536	-3.084576	1.757947
C	3.221947	-3.521337	3.078887
H	1.578178	-0.090740	2.280456
H	1.702736	-0.901103	4.625766
H	2.763570	-3.086602	5.134472
H	3.533866	-3.671006	0.933075
H	3.687663	-4.475950	3.298177
H	-0.511173	0.079896	-3.483521
H	2.407901	-2.986426	-2.807746
H	1.486934	3.032549	0.693808
H	4.955412	0.544863	1.131811
H	-0.935629	-2.805901	-1.304720
H	-1.393683	-2.109024	2.893384
H	-0.732843	2.877573	-2.063574
H	-1.635740	3.385393	2.089624

104

TS3

Al	-1.902074	0.523068	-1.168180
Zn	0.320561	-0.024118	-0.265637
N	2.262382	-0.101231	-0.255004
N	0.172629	-0.768836	1.586582
N	-3.399398	-0.461519	-0.624706
N	-2.468365	2.197093	-0.569536

C	2.780479	-1.256996	0.197163
C	2.041595	-2.131781	1.015650
C	0.983145	-1.778858	1.890441
C	4.207576	-1.726818	-0.196642
C	4.877619	-0.849130	-1.267438
C	5.120159	-1.800379	1.040711
C	4.075659	-3.139224	-0.818254
C	0.832162	-2.625657	3.180317
C	0.959095	-4.124915	2.820823
C	-0.531744	-2.454656	3.871597
C	1.954471	-2.254278	4.168642
C	2.996950	1.109581	-0.282419
C	3.660963	1.539013	0.872575
C	4.341242	2.753248	0.886388
C	4.347947	3.567186	-0.244588
C	3.658951	3.157075	-1.384888
C	2.981707	1.943286	-1.402600
C	-0.444027	0.115645	2.505247
C	0.352470	0.923883	3.324919
C	-0.226545	1.897106	4.134540
C	-1.606604	2.094304	4.121196
C	-2.400856	1.306535	3.289429
C	-1.825600	0.329195	2.483279
C	-5.618629	-0.843803	0.345819
C	-4.483651	0.066368	-0.036225
C	-4.588353	1.429847	0.269093
C	-3.619558	2.427576	0.072623
C	-3.872941	3.795267	0.640627
C	-3.294707	-1.874855	-0.862466
C	-3.879313	-2.444120	-1.994121
C	-3.693348	-3.797450	-2.263303
C	-2.914381	-4.582359	-1.413535
C	-2.318912	-4.008237	-0.292457
C	-2.503720	-2.654940	-0.015970
C	-1.418687	3.181316	-0.603983
C	-1.198771	3.922738	-1.762855
C	-0.110953	4.793169	-1.826311
C	0.763068	4.902947	-0.748035
C	0.548052	4.147537	0.403178
C	-0.543743	3.289026	0.480885
H	2.514630	-3.077550	1.217847
H	4.213431	-0.686107	-2.121985
H	5.773316	-1.363164	-1.628899
H	5.193071	0.122836	-0.886967
H	5.271823	-0.809448	1.478223
H	6.100912	-2.193033	0.753149
H	4.706130	-2.457328	1.810884
H	3.724317	-3.891556	-0.109094
H	5.056823	-3.465004	-1.176407
H	3.388918	-3.123941	-1.670580
H	0.265848	-4.402889	2.020114
H	0.714629	-4.724138	3.702264

H	1.967842	-4.409258	2.514460
H	-0.652616	-1.479626	4.343336
H	-0.623993	-3.207760	4.659416
H	-1.361542	-2.603110	3.172331
H	2.940894	-2.371853	3.710864
H	1.904088	-2.906126	5.046943
H	1.853600	-1.221189	4.512884
H	4.864761	3.064910	1.784953
H	4.882752	4.511139	-0.236870
H	3.652999	3.785130	-2.270451
H	0.404748	2.511311	4.768824
H	-2.056875	2.854407	4.750686
H	-3.477075	1.448439	3.265608
H	-5.254854	-1.671239	0.962313
H	-6.072757	-1.286427	-0.545400
H	-6.387403	-0.302050	0.895519
H	-5.502420	1.741876	0.756911
H	-3.632072	4.574898	-0.086243
H	-3.226075	3.953100	1.510450
H	-4.910467	3.903058	0.956045
H	-4.149966	-4.236829	-3.143866
H	-2.769861	-5.636016	-1.627517
H	-1.705376	-4.610854	0.369426
H	0.053986	5.380551	-2.723821
H	1.618443	5.567148	-0.806193
H	1.240139	4.213123	1.236344
F	-1.761758	0.216687	-2.919389
C	-0.133450	-0.572241	-2.805945
C	0.738277	0.191111	-3.547929
C	1.790240	-0.501938	-4.147649
C	1.886726	-1.887732	-4.033442
C	-0.158563	-1.946937	-2.722228
C	0.915218	-2.600239	-3.334927
H	0.624810	1.263694	-3.637840
H	2.526742	0.056039	-4.716780
H	2.708245	-2.413551	-4.506775
H	-0.920175	-2.493944	-2.180957
H	0.972645	-3.680965	-3.256816
H	-2.017351	-2.201218	0.842049
H	-4.461712	-1.820216	-2.664932
H	-0.723853	2.701683	1.376901
H	-1.880378	3.820268	-2.602330
H	2.465186	1.614777	-2.295923
H	3.638493	0.908893	1.757252
H	-2.452630	-0.282639	1.845143
H	1.430150	0.794185	3.302496

49

D1

Al	-0.118920	-1.282969	0.954377
N	1.277246	-0.796416	-0.390897
N	-1.519033	-0.721158	-0.371690

C	2.359929	0.269268	-2.331182
C	1.135765	-0.167638	-1.561363
C	-0.111866	0.109143	-2.129250
C	-1.367751	-0.193585	-1.588474
C	-2.576812	0.104689	-2.443059
C	2.594358	-1.188255	0.010083
C	3.275005	-0.479645	0.999662
C	4.536915	-0.897404	1.416900
C	5.124649	-2.028248	0.855639
C	4.437717	-2.747992	-0.120944
C	3.175688	-2.334800	-0.537056
C	-2.846951	-0.942222	0.114579
C	-3.623919	0.125052	0.571513
C	-4.893134	-0.106241	1.093054
C	-5.394439	-1.404499	1.173497
C	-4.615880	-2.470722	0.730587
C	-3.345686	-2.242680	0.205632
H	2.914962	-0.592262	-2.712950
H	3.038808	0.823289	-1.676362
H	2.080040	0.903665	-3.173367
H	-0.107097	0.594166	-3.097285
H	-3.124465	0.963487	-2.046046
H	-3.265696	-0.744194	-2.450151
H	-2.277607	0.330314	-3.467344
H	5.058303	-0.339172	2.188472
H	6.106166	-2.354336	1.184519
H	4.880642	-3.640130	-0.552779
H	-5.489709	0.730787	1.442643
H	-6.382386	-1.583572	1.585406
H	-4.993473	-3.486225	0.797528
F	-1.623851	2.728289	-0.598076
C	-0.440691	2.754474	0.059160
C	0.678162	3.221921	-0.612237
C	1.884671	3.278921	0.080103
C	1.953139	2.874314	1.413209
C	-0.401535	2.322379	1.374213
C	0.814648	2.389826	2.052851
H	0.594635	3.531030	-1.647733
H	2.773106	3.645403	-0.424293
H	2.895366	2.930054	1.948672
H	-1.298752	1.946160	1.852903
H	0.865934	2.051500	3.082460
H	2.626062	-2.900052	-1.284190
H	2.806365	0.397128	1.431842
H	-2.731469	-3.069955	-0.136120
H	-3.228513	1.133592	0.505367

49

DTS1*

N	1.384628	-1.429352	0.261480
C	1.226438	-2.723065	-0.014140
C	-0.032774	-3.338734	-0.080242

C	-1.281285	-2.701249	-0.018052
N	-1.418160	-1.405063	0.257661
H	-0.041710	-4.404465	-0.269840
Al	-0.006489	-0.140886	0.761696
C	2.440451	-3.581920	-0.275935
H	3.220927	-3.389699	0.465053
H	2.177199	-4.640131	-0.253916
H	2.866546	-3.353717	-1.257509
C	-2.509264	-3.538430	-0.284539
H	-2.933823	-3.294283	-1.262953
H	-2.263212	-4.600898	-0.272371
H	-3.285001	-3.340194	0.459880
C	2.676304	-0.825803	0.165276
C	3.358260	-0.450845	1.324096
C	3.206722	-0.501141	-1.085525
C	4.572986	0.224879	1.229716
C	4.423918	0.167463	-1.175333
C	5.110531	0.532818	-0.018111
H	5.095518	0.516652	2.135173
H	4.827423	0.419174	-2.151098
H	6.054350	1.063785	-0.089116
C	-2.700591	-0.781520	0.162183
C	-3.218839	-0.433511	-1.087384
C	-3.385185	-0.413850	1.321745
C	-4.427268	0.251128	-1.175607
C	-4.591285	0.277329	1.229047
C	-5.117051	0.608597	-0.017845
H	-4.821603	0.520747	-2.150349
H	-5.116278	0.562841	2.135086
H	-6.054171	1.151479	-0.087499
F	0.007217	0.771957	-1.047569
C	0.071238	4.834760	0.061734
C	-1.143296	4.151539	-0.027889
C	-1.181751	2.770578	-0.177407
C	0.027559	2.065452	-0.162295
C	1.258676	2.731830	-0.175940
C	1.263548	4.113406	-0.026121
H	2.185990	2.172855	-0.239579
H	2.217189	4.631832	0.014631
H	0.088253	5.913126	0.176986
H	-2.080074	4.699932	0.011540
H	-2.125931	2.240870	-0.242032
H	2.925074	-0.682867	2.291948
H	2.644372	-0.754008	-1.978946
H	-2.960919	-0.664360	2.288965
H	-2.654229	-0.682132	-1.980576

49

D2

Al	-0.000015	-0.101104	-0.656474
N	1.410103	-1.135780	0.101535
N	-1.410144	-1.135764	0.101545

C	2.481163	-2.991843	1.291558
C	1.256816	-2.257485	0.805812
C	-0.000025	-2.793222	1.127251
C	-1.256864	-2.257474	0.805818
C	-2.481210	-2.991830	1.291574
C	2.725187	-0.630123	-0.164676
C	3.366680	-0.958331	-1.358990
C	4.624912	-0.429916	-1.636915
C	5.238302	0.434036	-0.732137
C	4.585085	0.777227	0.450235
C	3.327881	0.251487	0.733212
C	-2.725222	-0.630085	-0.164656
C	-3.366750	-0.958313	-1.358946
C	-4.624971	-0.429867	-1.636865
C	-5.238316	0.434137	-0.732107
C	-4.585067	0.777344	0.450243
C	-3.327875	0.251572	0.733214
H	3.071587	-2.354784	1.956477
H	3.127888	-3.256905	0.450202
H	2.204597	-3.900059	1.827022
H	-0.000028	-3.712892	1.697440
H	-3.127958	-3.256871	0.450231
H	-3.071608	-2.354782	1.956526
H	-2.204637	-3.900059	1.827011
H	5.121102	-0.686841	-2.567433
H	6.216127	0.849492	-0.953708
H	5.048510	1.465952	1.149414
H	-5.121187	-0.686809	-2.567365
H	-6.216132	0.849617	-0.953674
H	-5.048458	1.466104	1.149410
F	-0.000025	-0.255070	-2.343297
C	0.000023	1.734808	0.021288
C	0.000078	1.960731	1.409072
C	0.000117	3.245371	1.950339
C	0.000106	4.352157	1.102494
C	0.000006	2.870031	-0.804497
C	0.000049	4.161975	-0.277710
H	0.000090	1.111084	2.093062
H	0.000158	3.385268	3.028113
H	0.000138	5.356694	1.515863
H	-0.000047	2.744777	-1.884931
H	0.000038	5.020598	-0.943464
H	2.796714	0.532165	1.637382
H	2.866670	-1.613754	-2.065195
H	-2.796692	0.532258	1.637372
H	-2.866782	-1.613779	-2.065139

6. References

[S1] A. Friedrich, J. Pahl, H. Elsen, S. Harder, *Dalton Trans.* **2019**, *48*, 5560-5568.

[S2] P. Budzelaar, A. B. van Oort, A. G. Orpen, *Eur. J. Inorg. Chem.* **1998**, 1485-1494.

[S3] A. Friedrich, J. Eyselein, J. Langer, S. Harder, *Organometallics* **2021**, *40*, 448-457.

[S4] V. Balasanthiran, M. H. Chisholm, K. Choojun, C. B. Durr, P. M. Wambua, *Polyhedron* **2016**, *103*, 235-240.

[S5] P. Romanato, S. Duttwyler, A. Linden, K. K. Baldridge, J. S. Siegel, *JACS* **2010**, *132*, 7828-7829.

[S6] C. Cui, H. W. Roesky, H.-G. Schmidt, M. Noltemeyer, H. Hao, F. Cimpoesu, *Angew. Chem. – Int. Ed.* **2000**, *39*, 4274-4276.

[S7] N. J. Hardman, B. E. Eichler, P. P. Power, *Chem. Comm.* **2000**, 53, 1991-1992.

[S8] F. Rekhroukh, W. Chen, R. K. Brown, A. J. P. White, M. R. Crimmin, *Chem. Sci.* **2020**, *11*, 7842-7849.

[S9] P. Losch, J.F. Kolb, A. Astafan, T.J. Daou, P. Pale, B. Luis, *Green Chem.* **2016**, *18*, 4714

[S10] Rigaku Oxford Diffraction, **2019**, CrysAlisPro Software system, version 1.171.40.53, Rigaku Corporation, Oxford, UK.

[S11] O. V. Dolomanov, L. J. Bourhis, R.J. Gildea, J. A. K. Howard and H. Puschmann, *J. Appl. Cryst.*, **2009**, *42*, 339–341.

[S12] G. M. Sheldrick, *Acta Cryst. A,* 2015, **71**, 3–8.

[S13] G. M. Sheldrick, *Acta Cryst. C,* 2015, **71**, 3–8.

[S14] A. Thorn, B. Dittrich and G. M. Sheldrick, *Acta Cryst. A,* 2012, **68**, 448–451.

[S15] Rigaku Oxford Diffraction, **2019**, CrysAlisPro Software system, version 1.171.40.67a, Rigaku Corporation, Oxford, UK.

[S16] https://checkcif.iucr.org/, 2021.

[S17] M. J. Frisch, G. W. Trucks, H. B. Schlegel, G. E. Scuseria, M. A. Robb, J. R. Cheeseman, G. Scalmani, V. Barone, B. Mennucci, G. A. Petersson, H. Nakatsuji, M. Caricato, X. Li, P. H. Hratchian, A. F. Izmaylof, J. Bloino, G. Zheng, J. L. Sonnenberg, M. Hada, M. Ehara, K. Toyota, R. Fukuda, J. Hasegawa, M. Ishida, T. Makajima, Y. Honda, O. Kitao, H. Nakai, T. Vreven, J. A. Montgomery, J. E. Peralta, F. Ogilaro, M. Bearpark, J. J. Heyd, E. Brothers, K. N. Kudin, V. N. Staroverov, T. Keith, R. Kobayashi, J. Normand, K. Raghavachari, A. Rendell, J. C. Burant, S. S. Iyengar, J. Tomasi, M. Cossi, N. Rega, J. M. Millam, M. Klene, J. E. Knox, J. E. Cross, V. Bakken, C. Adamo, J. Jaramillo, R. Gomperts, R. E. Stratmann, O. Yazyev, A. J. Austin, R. Cammi, C. Pomelli, J. W. Ochterski, R. L. Martin, K. Morokuma, V. G. Zakrezewski, G. A. Voth, P. Salvador, J. J. Dannenberg, S. Dapprich, A. D. Daniels, O. Farkas, J. B. Foresman, J. V. Ortiz, J. Cioslowski, D. J. Fox, Gaussian, Gaussian, Inc., Wallingford CT, **2013**.

[S18] J. Chai, M. Head-Gordon, *Phys. Chem. Chem. Phys.* **2008**, 6615–6620.

[S19] W. J. Hehre, L. Radom, P. v. R. Schleyer, J. A. Pople, Ab Initio Molecular Orbital Theory by W. J. Hehre, L. Radom, P. v. R. Schleyer, J. A. Pople, John Wiley, New York, **1986**.

[S20] T. Clark, J. Chandrasekhar, G. W. Spitznagel, P. v. R. Schleyer, *J. Comp. Chem*. **1983**, *4*, 294–301.

[S21] E. F. Silva, H. F. Svendsen, K. M. Merz, *J. Phys. Chem. A* 2009, 6404–6409.

[S22] A. E. Reed, R. B. Weinstock, F. Weinhold, *J. Chem. Phys.* 1985, **83**, 735–746.

[S23] N. van Eikema Hommes, Molecule, Erlangen, **2016.**

[S24] T. A. Keith, *AIMAll (Version 17.11.14),* TK Gristmill Software, Overland Park KS USA, **2017**.

[S25] R. F. W. Bader, *Chem. Rev*. 1991, **91**, 893–928.

Author Contributions

A. F. and J.E. conducted the experimental work. DFT calculations were done by J.E.; crystal structure determination was done by J. L. and C. F. evaluated NMR data. The results were reported by S.H., A.F., J.E. and C. F whereas S.H. was additionally responsible for supervision.
